# Internationally educated nurses’ experiences of recruitment - An ethical perspective

**DOI:** 10.1177/09697330251350391

**Published:** 2025-06-24

**Authors:** Pauliina Oja-Lipasti, Ashlee Oikarinen, Suleiman Kamau, Sepideh Petäistö, Kristina Mikkonen, Heli-Maria Kuivila

**Affiliations:** Seinäjoki University of Applied Sciences; 6370 University of Oulu; 6370 University of Oulu; Oulu University Hospital and University of Oulu; 6370 University of Oulu

**Keywords:** Diversity, ethics, internationally educated nurses, recruitment

## Abstract

**Background:** International migration among nurses in the healthcare workforce has increased significantly, with the number of internationally educated nurses in higher-income OECD countries doubling since 2000. These nurses frequently encounter challenges with competence recognition and obtaining local licences, which can hinder their ability to work effectively. Additionally, they often face misleading job information, discrimination, and exploitation, underscoring the urgent need for ethical recruitment and employment practices.

**Aim:** The aim is to describe internationally educated nurses’ experiences of recruitment to Finland, focussing on ethical considerations.

**Methods:** A qualitative study design was employed to explore nurses’ experiences. Data were collected in spring and summer 2024 from 22 internationally educated registered nurses with degrees from outside the EU, who were either working in healthcare or completing a top-up nursing degree in Finland. Content analysis was employed to examine the data, revealing ten key categories associated with ethical recruitment.

**Ethical considerations:** A research permit was obtained from the university overseeing the study. The researchers ensured that all procedures adhered to ethical research standards, including obtaining participants’ informed consent and maintaining the confidentiality of the data.

**Results:** The findings revealed a spectrum of experiences, from supportive practices to notable challenges. Key themes emerged related to the recruitment process, linguistic and cultural adaptation, and workplace integration. Positive encounters included supportive recruitment companies and structured orientation programs, while significant difficulties involved unmet contractual promises, inadequate language support, and cultural barriers.

**Conclusions:** Difficulties with learning Finnish and cultural adaptation impede integration, underscoring the need for employers to provide robust language training and adopt ethical practices. Enhanced support systems are crucial to improving the integration and job satisfaction of internationally educated nurses in Finland.

## Introduction

The global nursing shortage was 5.9 million in 2020 (World Health Organisation [WHO], 2020).^1^ The International Council of Nurses (ICN) March 2023 report highlighted the pandemic’s impact on nurses and the global workforce, showing 40–80% experienced work-related stress and over 20% considered leaving the field (ICN, 2023, p. 15; 20–26).^2^ Almost half of Finnish nurses wanted to leave during and after the pandemic (Sihvola et al., 2023).^3^ WHO predicts a global need for 4.8 million more nurses and midwives by 2030 (WHO, 2024),^4^ with Finland needing an estimated 30,000 (Ensio et al., 2019).^5^ Ageing populations and increasing chronic diseases exacerbate the shortage. ICN’s plan proposes training more nurses, supporting vaccination programs, and ethical recruitment, with higher-income countries often recruiting from lower-income ones (ICN, 2023, p. 4–9).^2^

Cultural diversity globally has increased, with international migration rising significantly in the past 50 years. By the 2020s, there were nearly 300 million international migrants, up from less than 100 million in the 1970s. International migrants are those who have permanently moved from their country of birth (McAuliffe and Triandafyllidou, 2021).^6^ According to a report published in 2021 by the Organisation for Economic Co-operation and Development (OECD), the number of internationally educated nurses in higher-income OECD countries has doubled compared to 2000 (Socha-Dietrich and Dumont, 2021).^7^

Nurses from other countries should have the right to develop their own professional career and personal skills as a nurse, as well as the right to free movement for work (ICN, 2019a).^8^ Ethical actions in the recruitment of international nurses should be introduced and developed in WHO member countries based on WHO’s global guidelines. Currently, monitoring of these activities is insufficient due to the lack of official regulations in the member countries (ICN, 2023, p. 8).^2^ WHO’s global guidelines (WHO, 2010)^9^ and International Recruitment Integrity Systems (IRIS)^10^ recommend certain practices for recruiting international nurses, and in Finland, the Ministry of Social Affairs and Health (STM) has published its own responsibility recommendation for the recruitment of social and health personnel in 2023.^9–11^

The purpose of the research was to study the recruitment of internationally educated nurses to Finland from an ethical perspective as told by the nurses. The aim was to produce knowledge about international recruiting that can be used nationally and internationally in developing internationally educated nurses ethical recruiting.

## Background

Internationally educated nurses (IENs) are those educated outside the country where they practice (ICN, 2019a).^8^ One-third of them working in OECD countries come from lower-middle-income nations and another third from lower-income nations, with the Philippines, India, and Poland being the top origins (Socha-Dietrich and Dumont, 2021).^7^ Nurses move to more developed countries for better career prospects and salaries and to support their families, sometimes moving with their families for better opportunities (Hughes, 2022; Konlan et al., 2023).^12,13^ In Finland, reasons for immigration include further studies, permanent relocation, or earning money to send back home (Isakov et al., 2023).^14^ These nurses bring linguistic and cultural benefits to Finnish healthcare, helping alleviate the nursing shortage and promoting a culturally diverse society (Calenda et al., 2019; Kamau et al., 2023).^15,16^

Challenges faced by internationally educated nurses include discrimination, career advancement barriers, bullying, and racism, both from colleagues and patients. Inadequate support for learning the language and culture is a common issue (Al-Yateem et al., 2022; Kamau et al., 2023; Thomas et al., 2021).^16–18^ Many nurses report insufficient orientation and mentorship in their workplaces, though those introduced to the culture beforehand found the immigration process easier (Kamau et al., 2023; Thomas and Lee, 2023).^16,19^ Issues with recruitment companies involve contract changes without consent and a lack of contract transparency (Shaffer et al., 2020; Zulfiqar et al., 2023).^20,21^ Also, ethical problems arise, for example, in the form of high fees and breach of contract payments and with some nurses receiving lower salaries than locals (Shaffer et al., 2020; Thomas and Lee, 2023).^19,20^ Legalisation and recognition of skills are challenging, impacting job roles and integration (Cubelo, 2020; Isakov et al., 2023; Kamau et al., 2023; Sheehy et al., 2024),^14,16,22,23^ misleading information about jobs, discrimination, and high exploitation risk due to language barriers (ICN, 2019a).^8^ Positive experiences include clear and fast job search processes, employer-paid visas, travel, knowing what to expect upon arrival, receiving organised transportation with a welcome, and helping nurses feel at home in the new country (Pressley et al., 2023).^24^

Ethical recruitment, recommended by ICN and IRIS, ensures legal, fair, and transparent hiring, respecting nurses’ rights and promoting professional development (ICN, 2019b; IRIS, n. d.).^10,25^ Ethical recruitment follows international labour and human rights, respecting laws and professional conduct, prohibiting recruitment fees, and ensuring confidentiality and fair employment terms (IRIS, n.d.).^10^ Around 50 foreign-educated nurses are recruited to Finland annually (OECD, n. d.).^26^ Ethical recruitment guidelines by the European hospital and healthcare employers’ association (HOSPEEM) and the European public service union (EPSU, 2008), WHO (2010), and Finland’s STM (2023) emphasise honesty, fairness, and transparency. International workers should have the same rights as locals, with recruitment preventing human trafficking and exploitation (EPSU, 2008; STM, 2023; WHO, 2010).^9,11,27^

More research is needed on the process of skills recognition and legalisation for nurses from outside the European Union/European Economic Area (EU/EEA) (Isakov et al., 2023).^14^ Clear guidelines and recruitment strategies are essential (Gillin and Smith, 2019).^28^ International studies have highlighted issues in the recruitment process of internationally trained nurses to the target country (Al-Yateem et al., 2022; Pressley et al., 2023).^17,24^ Common complaints include the issues mentioned earlier (Shaffer et al., 2020; Thomas and Lee, 2023),^19,20^ particularly among nurses from developed countries (Zulfiqar et al., 2023).^21^ However, the experiences of IENs regarding ethical recruitment have received less interest previously (Isakov et al., 2023; Kamau et al., 2023).^14,16^ Hence, studying the experiences of internationally educated nurses’ recruitment to high-income countries from an ethical perspective is important for ensuring fair recruitment practices, improving the integration process, and ultimately enhancing patient care and healthcare outcomes. For this reason, this study focused on IENs who had their background nursing degree from their home country, more specifically from outside of the EU, and who were recruited to Finland to either work in the healthcare sector or to study top-up nursing degree. Top-up nursing degree programmes are designed for registered nurses educated outside of Finland to upskill their degree in order to get a qualification as a registered nurse in Finland (Arcada University of Applied Sciences, n. d.).^29^

## Methods

### Research design

The qualitative research design of this study aims to explain and understand people and various phenomena. A key feature was studying IENs’ lived experiences in their natural environments (Mays and Pope, 2020, pp. 1–4; Polit and Beck, 2022, pp. 161–174).^30,31^ This study used a qualitative method from an inductive starting point to produce new data-based information on an insufficiently studied topic in Finland (Kyngäs, 2019a, pp. 3–11).^32^

### Aim and research question

The purpose of this study was to examine internationally educated nurses’ experiences of recruitment to Finland, focussing on ethical considerations. The study aimed to answer the research question: What kind of experiences do internationally educated nurses have about recruitment to Finland from an ethical perspective?

### Participants

A total of 22 internationally educated registered nurses participated in the study. The inclusion criteria were being a registered nurse born abroad and recruited to Finland, either to work within the Finnish primary or tertiary healthcare system or to study a top nursing degree and be educated as a registered nurse outside of the EU countries. The exclusion criteria were whether the participant was educated originally as a nurse in Finland or in other EU countries or is originally a Finnish citizen. A total of 22 registered nurses participated in the study, including nine males and 13 females, representing five nationalities: the Philippines, Nepal, Egypt, Vietnam, and Japan (Table 1). All held nursing degrees from outside the EU and were either working in healthcare or pursuing a top-up nursing degree in Finland. Participants were recruited to Finland for these purposes and were located in various regions. The researchers had no prior relationships with the participants.

**Table 1.** Participant’s demographics.

Demographics	*n*
Gender	
Male	9
Female	13
Country of origin	
Philippines	16
Nepal	2
Egypt	2
Vietnam	1
Japan	1
Recruitment purpose	
Recruited to work in Finland	12
Recruited to study top-up nursing degree in Finland	10
Current job title	
Caregiver	4
Practical nurse	7
Nursing student	2
Registered nurse	9

### Data collection

The participants were recruited through snowball sampling, that is, eligible participants were first contacted through email, and those who were successfully recruited suggested further potential participants. More specifically, the researcher (PO-L) advertised the research on different social media channels meant for international professionals in Finland. The IENs who were eligible for the study and willing to participate gave their consent through Webropol, where they also answered questions related to their backgrounds, such as country of origin and country of study. After that, the researcher (PO-L) contacted them electronically to agree on interview times. Data were collected in the spring and summer of 2024. Online, semi-structured individual interviews were recorded in Microsoft Teams as audio. The researcher (PO-L) and each participant were at home during the interviews, with no other persons in the same room. A semi-structured thematic interview allowed the researcher (PO-L) to ask about essential topics and gain more extensive information, as participants could answer more openly (Hinton and Ryan, 2020, pp. 43–45).^33^ The semi-structured interviews were built on five general themes based on previous studies (Al-Yateem et al., 2022; Isakov et al., 2023; Kamau et al., 2023; Pressley et al., 2023; Shaffer et al., 2020; Thomas and Lee, 2023),^14,16,17,19,20,24^ including recruitment, legalisation as nurse, recognition of previous competence, and reasons of applying and staying in Finland. A total of 21 interviews were conducted in the English language, with the remaining one conducted in Finnish due to the participant’s request. Collected data were transcribed verbatim. Data saturation was reached.

### Data analysis

Interview data were transcribed into 221 pages of data, which were analysed using inductive content analysis (Kyngäs, 2019b).^34^ Participants and their original expressions were numbered to protect their anonymity and to help the researcher track original expressions during the data analysis process. Critical realism is described as being able to understand the world through other ways than observing, such as from people’s experienced events (Al-Sharif, 2021; Mikkonen and Kyngäs, 2019).^35,36^ This philosophy acknowledges that each person’s experiences are unique, and those experiences are captured through their senses, emotions, and linguistic and cultural aspects (Lauzier-Jobin and Houle, 2021).^37^ The inductive analysis process started with the researcher reading and familiarising the transcribed text. During this process, the meaning units were formed from original expressions connected to the research question. The meaning units were then arranged through data coding (*n* = 801). The codes were analysed and categorised with similar meanings, and the sub-categories (*n* = 178) were established during categorisation. The sub-categories were labelled according to the research question and then further arranged into categories (*n* = 33). The categories were arranged into main categories (*n* = 10) that described IEN’s experiences of recruitment to Finland from an ethical perspective. The example of the content analysis is shown in Tables 2 and 3.

**Table 2.** Creating the subcategory ‘Needing to pass requirements before coming to Finland’.

Meaning unit	Code	Subcategory
‘Because I applied when I was in Philippines, so we took first we passed these requirements like our transcript of records, diploma, and then after that, maybe after 3 weeks they have scheduled for interview so (13)’	First we need to pass the requirements like the transcript of records and diploma (13)	Needing to pass requirements before coming to Finland
‘All the proceedings that I have made I passed, I have been accepted in one of the prestigious university here in Finland, which is the *** university. They yeah, they gave me an opportunity to study there (12)’	After I passed all the proceedings, I was accepted to a university in Finland (12)	
‘So I’ve submitted the initial requirements and then like diplomas and so on and so forth and then after a while they contacted me that I have to attend an online orientation and after that (15)’	After submitting the requirements, I attended online orientation (15)

**Table 3.** Creating the main category ‘Preparations for recruitment’.

Subcategory	Category	Main category
Finding out about the recruitment company from a friend	Discovering the recruitment company	Preparations for recruitment
Finding out about the recruitment company by myself		
Hearing about recruitment company from a relative		
Needing to pass requirements before coming to Finland	Initial steps in recruitment preparation	
Recruitment process required taking Finnish language exam		
Recruitment company requiring lot of documents		
Language should be studied first before coming to Finland		
Finding the information for coming to Finland by myself		
Having an interview with recruitment company for coming to Finland		

### Ethical considerations

The study did not require prior ethical evaluation by the University of Oulu Human Sciences Ethics Committee,^38^ as specified by the Medical Research Act (488/1999, 2010)^39^ and the guidelines of Research Ethics Advisory Board [TENK] (Keiski et al., 2023).^40^ The study did not concern minors and did not concern physical integrity. A research permit was secured from the University of {blinded} and recruitment of participants commenced following approval. All data were securely stored during the research process. Participants or their organisations cannot be identified at any stage of research or reporting according to the European Union’s Data Protection Act (1050/2018, 2018)^41^ and the General Data Protection Regulation (GDPR) (European Parliament, 2016).^42^ Participants received a comprehensive information letter in both Finnish and English, detailing the study’s purpose, goals, and pseudonymity measures. Informed consent was obtained electronically via the Webropol survey tool, ensuring fairness, equality, voluntary participation, and participant autonomy (Stadnick et al., 2021).^43^ The study adhered to the ethical standards outlined by TENK and the Declaration of Helsinki, ensuring participants were informed of their right to withdraw consent without consequences (Kohonen et al., 2019; WMA, 2022).^44,45^ Special coding was employed to pseudonymise data and protect participant identities. Participants had opportunities to ask questions before and after interviews, ensuring transparency and addressing any concerns related to the research.

## Results

In this study, the recruitment process was understood to encompass not only initial selection and relocation but also orientation, language support, and early-phase integration into the workplace. The ten main categories that emerged from the IEN’s descriptions of their experiences were as follows: 1) preparations for recruitment; 2) experience with the recruitment company; 3) experience of the recruitment process; 4) costs and fees associated with recruitment; 5) recognition and validation of prior nursing competence; 6) language learning journey; 7) cultural and workplace integration; 8) employer-provided support and resources; 9) colleagues and workplace environment; and 10) overall experience as an international nurse in Finland (see Tables 2–4 and Supplemental File 1).

**Table 4.** Experiences of recruitment.

Category	Main category
Discovering the recruitment company	Preparations for recruitment
Initial steps in recruitment preparation	
Satisfaction level with the recruitment company	Experience with the recruitment company
Fulfilment of recruitment company’s promise	
Informative nature of recruitment company	
Challenges encountered with the recruitment company	
Promises made by recruitment company	
Positive experience with the recruitment process	Experience of the recruitment process
Challenges during recruitment process	
Time consumption in the recruitment process	
Tuition fees	Costs and fees associated with recruitment
Costs associated with the recruitment process	
Challenges related to recruitment process fees	
Recruitment company sponsored fees	
Arrangements related to recruitment process	
Recognition of prior nursing experience	Recognition and validation of prior nursing competence
Lack of recognition for previous nursing experience	
Recognition of previous nursing degree	
Non-recognition of previous nursing degree	
Studying and learning the language	Language learning journey
Challenges with the Finnish language	
Negative experiences as a foreigner in Finland	Cultural and workplace integration
Challenges with cultural adaption	
Employer-organised training and orientation at work	Employer-provided support and resources
Negative experiences with workplace orientation	
Positive interactions with colleagues	Colleagues and workplace environment
Challenges with colleagues at work	
Limitations at workplace	
Receiving support in educational and work settings	
Positive experiences as an international nurse in Finland	Overall experience as an international nurse in Finland
Challenges as an international nurse in Finland	
Challenges and problems of working life	
Professional successes and achievements	

*Preparations for Recruitment* involved both discovering recruitment companies and taking the initial steps required for the process. Many internationally educated nurses (IENs) learnt about recruitment companies through friends, relatives, or independent searches via advertisements and online resources. The initial phase often required meeting specific criteria, such as passing a Finnish language exam and completing interviews. A significant challenge during this stage was navigating the extensive documentation process, which many nurses had to manage independently. Participants raised ethical concerns about the sufficiency of guidance and transparency provided by recruitment companies in the early stages, such as structured language training before arrival in Finland. As one nurse reflected: ‘*Learning in school, doing the training in the hospital, and eventually looking for a job – sending out your resume and going through interviews – it could have been much better structured. If they are recruiting nurses, then nursing-specific language training should start even before we leave our home country’. (IEN nr 5)*

IENs’ *experiences with recruitment companies* varied, encompassing their satisfaction levels, the fulfilment of promises, the clarity of information provided, and the challenges encountered. Nurses described their recruitment companies positively, highlighting their responsibility, ethical conduct, and approachability. Some companies were praised for their proactive support, such as meeting nurses at the airport and arranging housing without additional fees. These positive experiences highlight the ethical importance of transparency and fairness in fulfilling recruitment-related commitments. As one nurse shared:

‘In my experience with the agent, everything was handled legally *–* papers, documents, and everything. When it came to doing what was right, I personally didn’t have any major issues as far as I remember’. (IEN nr 21)

A key measure of satisfaction was the fulfilment of promises. Many IENs received the agreed-upon salary, and in some cases, even more than expected. Recruitment companies generally provided useful information about housing, work conditions, cultural adaptation, and financial matters in Finland. However, delays and issues with documentation were occasionally reported. Despite these positive aspects, challenges were also noted. Some IENs found recruitment companies to be untruthful, lacking in transparency, or failing to uphold their commitments. One of the most common complaints was the high cost of housing arranged by recruitment companies, and participants raised ethical concerns regarding fairness and informed consent. As one nurse described:

‘The only thing I’m unsure about *–* whether it’s unethical or not *–* is how high our rent is. We live in the countryside, so we expected it to be much lower than in the city. But it’s doubled. I think they take a percentage, which is fine, but maybe 10% would be reasonable. Doubling it is just too much’. (IEN nr 19)

The promises made by recruitment companies varied significantly. While some companies clearly outlined employment terms and support services, others made vague or unfulfilled commitments. Some nurses were promised assistance with bringing their families to Finland or help with documentation, while others received little clarity about their future employment or additional support.

*The recruitment process* brought both positive and negative experiences for IENs, with time consumption being a significant factor. Positive experiences were characterised by smooth, well-organised procedures and adequate support. As one nurse described: ‘*It was really good because at that time, there was no pandemic yet, so I was able to have my language training face-to-face. The process was very smooth, even with the visa application and everything. Everything went well’. (IEN nr 9)*

However, many nurses faced challenges during the recruitment process. Some described it as rough, emotionally draining, and difficult to navigate. A lack of clear guidance from recruitment agencies left some IENs feeling isolated and forced to manage much of the process on their own, relating to experiences of insufficient transparency and support during the recruitment process. One nurse explained*: ‘Talking about the process… yeah, it was a little bit hard, I found it difficult. The agency wasn’t entirely clear about everything. The tailor-made nursing program was the first of its kind from our country, and we were like the test batch – an experiment’. (IEN nr 18)*

Delays were a common issue, with long waiting times for visas and bureaucratic procedures. For many, the entire recruitment journey stretched over several months, adding to the stress and uncertainty. The combination of prolonged timelines, inconsistent support, and administrative hurdles made the process particularly challenging for some nurses.

*Costs and fees associated with recruitment* caused IENs to encounter various financial challenges during the recruitment process, including tuition fees, recruitment costs, and logistical expenses. Many nurses faced high costs and, in some cases, had to borrow money to cover these expenses. These financial pressures were experienced as ethical questions about cost transparency and equal opportunity for applicants. While some recruitment companies provided financial support *–* covering certain fees and supplying study materials, medical tests, and household essentials *–* others offered little assistance. One nurse highlighted the burden of travel expenses, stating: ‘*I hope that the next batch of nurses coming here won’t have to pay for their tickets anymore because it’s a significant amount of money, and not all nurses in the Philippines can afford it’. (IEN nr 16)*.

For some nurses, employer-provided support helped ease the financial strain. This assistance included covering the cost of flight tickets, schooling, language training, housing arrangements in Finland, and visa processing. However, the level of financial aid varied, leaving some IENs with considerable out-of-pocket expenses.

*Recognition and validation of previous nursing experience* varied among IENs. Some participants felt their prior expertise was acknowledged, positively impacting their employment contracts, salaries, and responsibilities. However, others faced difficulties when their previous nursing experience was not recognised, leading to mismatched job roles, underutilisation of their skills, and frustration. These disparities raised ethical concerns about fairness, respect for professional competence, and equal opportunities in employment. One nurse expressed this frustration: ‘*It’s not my area of experience. It’s a different setting, and I feel like I’m not in the right position. But I can’t say anything because otherwise, I’d be at home without work’. (IEN nr 10)*

*Language learning* was a central aspect of IENs’ experiences, encompassing both language acquisition and the challenges associated with learning Finnish. Many nurses received training, with some benefiting from free language courses. However, the learning process was often difficult, leading to frustration. While some nurses adapted well, others struggled, particularly with online learning formats. Difficulties with the Finnish language extended beyond the classroom, affecting workplace interactions, teamwork, and communication with patients. These challenges sometimes led to feelings of insecurity and exclusion. As one nurse shared: ‘*Maybe it’s on me, but sometimes I feel out of place. When they talk together and I don’t understand, I just tend to stay quiet and avoid mingling with them in the coffee room because I don’t know what they’re talking about’. (IEN nr 1)*

For many, learning Finnish was particularly demanding, and some nurses emphasised the need for greater language support from employers. These actions could be seen as ethical responsibilities of the employer and recruitment companies. Participants suggested that additional resources and structured workplace language training could help ease communication barriers and improve their overall integration.

*Cultural and workplace integration* presented significant challenges for many IENs, both in their daily lives and professional environments. These social barriers were raised up as ethical concerns about equal treatment and inclusion. The transition to Finland was not always smooth, with some nurses describing negative experiences upon arrival, including difficulties adapting to a new culture, social isolation, and unwelcoming attitudes from locals.

One of the most difficult aspects was encountering racism. Some IENs felt that Finnish society needed to adopt a more inclusive and accepting attitude towards immigrants. As one nurse expressed*: ‘Others, but especially people, are very racist in Finland. I’m so sorry’. (IEN nr 8)*

Cultural differences also contributed to workplace challenges. Some nurses reported feeling excluded or struggling to integrate into Finnish work culture, which impacted their confidence and sense of belonging. Miscommunication, differing workplace expectations, and a lack of cultural awareness among colleagues sometimes made it difficult for IENs to fully participate in their teams. To improve the integration process, many nurses emphasised the need for better employer support. They suggested that workplaces should provide more comprehensive orientation programs, including cultural training and clearer guidance on Finnish workplace norms. A more structured introduction to the work environment would help international nurses feel more prepared and supported as they transition into their roles.

*Employer-provided support* played a crucial role in helping IENs adjust to their new work environment. This support included employer-organised training and workplace orientation, covering essential areas such as nursing practices, fire safety, medication administration, ergonomics, first aid, and documentation. The length and quality of orientation varied, with some nurses receiving structured guidance in English, which was particularly appreciated. As one nurse shared:

‘When we started working, my instructor guided me in English, and my boss reassured me to take things one step at a time. That made it quite manageable for me’. (IEN nr 4)

However, not all experiences were positive. Disparities in support were seen as inconsistencies in ethical recruitment standards across different employers. Some nurses told that they have faced inadequate orientation, language barriers, and a lack of direct support from supervisors. The absence of clear guidance made it difficult for them to integrate smoothly into the workplace, highlighting the need for more structured and inclusive onboarding processes.

*The work environment and interactions with colleagues* played a significant role in shaping IENs’ experiences. Positive interactions included supportive colleagues who were patient, helped with translation, and assisted with learning Finnish. Many nurses appreciated that their coworkers were aware of their language learning process and made efforts to simplify communication. As one nurse shared: ‘*When we arrived, I think all my workmates were well-informed that we were still learning the language. They were very patient, teaching us and trying to use simpler words and language that suited us’. (IEN nr 2)*

However, not all experiences were positive. Some IENs faced challenges such as colleagues refusing to speak English, reporting them to supervisors instead of offering help, or being generally unhelpful. Workplace limitations also restricted some nurses from performing certain nursing skills, including medication administration, due to language barriers or lack of recognition of their prior training. Despite these challenges, workplace support—through feedback from supervisors, colleagues, and educational institutions *–* helped some IENs adapt and grow in their roles.

The licensing process for obtaining a registered nursing license in Finland also varied. Some IENs obtained their license quickly, sometimes within a month, while others had to complete additional studies *–* though often for a shorter period compared to starting a nursing degree from scratch. However, for some, the process was much more challenging, with certain nurses required to redo their entire nursing education before obtaining licensure in Finland.

*IENs’ overall experiences* were shaped by a mix of positive moments, challenges, work-life struggles, and professional achievements. Positive experiences included meaningful interactions with patients and colleagues, feeling respected in their roles, and gradually gaining responsibilities. However, challenges were also prevalent. Some nurses faced contractual restrictions, negative attitudes from colleagues or patients, and even discrimination. One nurse described how their situation required additional effort and support: ‘*I feel like I’m given more work because I need special guidance’. (IEN nr 3)*

Balancing work and study was another common struggle, as was the lack of institutional support and the differences in nursing practices and protocols compared to their home countries. Despite these obstacles, many IENs also celebrated professional achievements. They highlighted skill development, access to employment benefits, career progression opportunities, and the experience of working in diverse, international teams as key positives of their journey.

## Discussion

The research aimed to understand internationally educated nurses’ experiences of ethical recruitment to Finland, develop ethical recruitment practices, prevent unethical behaviour, and raise awareness among stakeholders about poor practices. Findings showed that IENs faced extensive documentation and language requirements, with mixed support from recruitment companies. The recruitment process had both smooth and difficult experiences, with significant costs and fees. Language learning was challenging, leading to frustration and exclusion, while cultural and work integration included racism and the need for more inclusive support. Employer-provided support varied, with some inadequate orientations and language barriers. IENs had both positive interactions and challenges with colleagues and the work environment. Recognition of prior competence varied, affecting employment contracts and skills utilisation. Overall, the experience as an international nurse included both positive interactions and significant challenges, along with professional achievements.

The experiences of IENs with recruitment companies in Finland present a mix of fulfilment and frustration. In this study, informants described that although many companies keep their promises about salary and initial support and provide important information about housing, work conditions, and culture, some nurses still face significant challenges, such as high housing costs, as it has been mentioned previously by Pressley et al. (2023).^24^ Positive aspects highlight a commitment to financial transparency and support, which is crucial for building trust, as it has been shown in previous research by Rajpoot et al. (2024).^46^ Recruitment companies play an important role in making the transition easier for newcomers by being informative and supportive despite occasional delays or issues with documentation. In this study, IENs recruited to Finland did not experience issues related to their contracts, such as not seeing the contract before signing or discovering changes made without consent, which have been reported in other countries. In some countries, there were also instances of breach of contract penalties (Shaffer et al., 2020).^20^ However, informants also reported dissatisfaction with certain recruitment companies due to untruthfulness and lack of information. This concern has also been noted by Rajpoot et al. (2024).^46^ The variation in promises made by recruitment companies was seen to lead to confusion and dissatisfaction among IENs. Addressing these ethical concerns not only improves the experiences of IENs but also enhances the reputation and credibility of recruitment companies. Accordingly, ethical recruitment practices should make all the promises realistic and achievable, not overpromising and under-delivering. Setting high standards for ethical recruitment will provide a means for recruitment companies to earn trust and ensure fairness, making positive changes in the lives of their recruits.

In this study, IENs indicated that the recruitment process involved substantial costs, placing a heavy financial burden on nurses, often requiring them to borrow money, leading to debt and financial stress. According to Kurup et al. (2024),^47^ similar financial challenges have been identified elsewhere. Ethically, it is crucial to consider the impact of these costs on the lives of the nurses. According to the International Labour Organization (2020),^48^ charging high fees and failing to provide comprehensive support can be seen as exploitative. As suggested by their ethical recruitment practises, no high fees should be charged for recruitment to prevent debt bondage for migrant workers (International Labour Organization, 2020).^48^ Recruitment companies and employers must strive to minimise the financial burden on international nurses by providing clear communication about costs, comprehensive financial support, and assistance with logistical arrangements (Thomas and Lee, 2023).^19^ Failing to provide such support can lead to feelings of exploitation and distrust among the nurses.

Results from this study highlight that language support is a critical component of the integration process for IENs. Language training and ongoing support can help them overcome communication barriers and improve their confidence and effectiveness at work, as it has been shown in previous research by Theron et al. (2024).^49^ Recruitment companies are ethically obligated to ensure that IENs receive adequate language support, facilitating better integration and professional development (STM, 2023).^11^ Many nurses described experiencing communication difficulties and feelings of marginalisation in the workplace. One of the enablers for the integration of IENs has been a successful mentorship program (Shiju et al., 2024).^50^ Learning Finnish is challenging yet significantly important during integration as stated by the participants. Many nurses emphasised the importance of studying the language before immigration. When language difficulties lead to challenges in the work community, it can also cause challenges in cultural adaptation. Experiencing racism and feeling unaccepted by locals leads to negative experiences as a foreigner, as also noted by Kamau et al. (2023) and Thomas et al. (2021).^16,19^ This highlights the need for society to adopt a more inclusive attitude towards immigrants (Almutairi et al., 2017).^51^ Some IENs indicated that frustration towards learning Finnish might influence decisions to leave Finland for English-speaking countries. For that, it is crucial for recruitment companies to ensure proper language training for IENs before immigration happens.

This research stated that employer-provided support is critical in the successful recruitment and integration of IENs. Workplace training was generally well-received, and receiving orientation in English facilitated a smoother transition. However, inadequate orientation and supervisory involvement pose challenges, highlighting the need for standardised and comprehensive programs as it has been noted by Martikainen et al. (2024).^52^ Recognition of prior nursing experience was seen to vary among informants, leading to feelings of underutilisation and mismatched employment contracts. Similar challenges have been identified by Sheehy et al. (2024).^23^ As it is validated by Kamau (2024),^53^ employer-provided support by nurse leaders and managers helps in the integration of IENs. The licensing process of different countries’ recruiting and study requirements also vary, creating disparities in how international nurses’ qualifications are recognised. From an ethical standpoint, comprehensive career planning and orientation are crucial for successfully integrating international nurses. Ethical recruitment practices should ensure that internationally educated nurses are fully informed about their opportunities in the host country. This includes whether they may need to work in lower-level positions, such as practical nurses, and the possibilities for further education and training as a registered nurse (Isakov et al., 2023).^14^ Both recruitment companies and prospective employers must address language barriers and ensure that orientation programs are thorough and effective. Training and support for native nurses in dealing with foreign colleagues are necessary to create a more understanding work community. Employer-provided support and training can minimise negative experiences for IENs (Kamau 2024; Theron et al., 2024).^49,53^ A more streamlined and transparent licensing process that fairly recognises the qualifications and experience of IENs is also needed to ensure consistency and equity in employment practices (Kurup et al., 2024).^54^ Ethical recruitment practices should be uniformed to support the fair treatment and integration of IENs.

### Trustworthiness of the study

This study ensured reliability by maintaining a curious yet critical stance throughout. Qualitative research reliability was evaluated using four main criteria: transferability, credibility, dependability, and confirmability, with six additional criteria available at the researcher’s discretion (Lincoln and Cuba, 1985; Kyngäs et al., 2019).^54,55^ Transferability involved transparently reporting participant criteria, backgrounds, and results, enabling assessment of the study’s applicability to similar situations without generalising findings (Kyngäs et al., 2019).^55^ The participants were from a wide variety of countries, both low- and high-income countries, so the results of the study reflect a wide range of experiences. The results could potentially aid in the ethical recruitment of healthcare professionals beyond nurses, though the study’s limitation is that it might not fully apply to nurses educated within the EU due to the free movement of healthcare workers (European Commission, n. d.).^56^ Credibility was ensured by systematically integrating credibility into the data through rigorous data collection methods and detailed documentation of the analytical process. Participants were nurses educated outside the EU, now in Finland. Semi-structured thematic interviews allowed participants to respond freely and reached data saturation (Kyngäs et al., 2019).^55^ To enhance reliability, the entire research process was described transparently. Results are published based on collected data without researcher bias. Due to the good English of the research interviewer (PO-L), the interviews conducted in English did not cause any issues. Content analysis was meticulously conducted using rigour methodology by recording and coding the data and organising it into categories. Content analysis was supported by research diaries and notes and with discussions between research group members so that the results could be verified. Confirmability was demonstrated by linking findings to the data with authentic quotes, ensuring that other individuals would reach the same conclusions from the same data (Kyngäs et al., 2019).^55^ Standards for reporting qualitative research (SRQR) checklist (O’Brien et al., 2014)^57^ was used to enhance the study’s trustworthiness.

### Strengths and limitations

This study had inherent strengths and limitations. The main strength was that the participants represented both nurses, who were recruited to work in Finland and nurses, who were recruited to study in top-up nursing programme in Finland. Participants also represented a variety of different nationalities. However, the participants were recruited from various regions in Finland, which may introduce regional biases in the experiences reported. Additionally, the inclusion criteria focused on nurses recruited to Finland from non-EU countries, excluding the experiences of nurses from EU countries. This limitation may affect the comprehensiveness of the study in capturing the full spectrum of internationally educated nurses’ experiences. However, the exclusion criteria were chosen because nurses educated within the EU have qualifications that meet the same EU standards, making their recruitment likely to be more ethical, for example, making it easier to obtain a nursing license in Finland and due to the free movement inside the EU.

## Conclusions

This study underscores the critical need for ethical recruitment practices, comprehensive linguistic and cultural training, and robust support systems to improve the integration and job satisfaction of internationally educated nurses (IENs) in Finland. Recruitment companies must ensure transparency, ethical compliance, and enhanced pre-arrival guidance, including structured language training and clear information about employment conditions. Addressing high recruitment fees and unmet promises requires stronger supervision, such as implementing certification systems and regular inspections to uphold ethical standards.

To foster a smoother transition, employers should provide structured workplace orientation, mentorship programs, and ongoing support for language learning. Ensuring fair recognition of prior nursing experience would enhance job satisfaction and career progression, while tackling racism and promoting inclusivity is critical for cultural adaptation. Digital tools and automated systems could further streamline the recruitment process, reducing delays and administrative burdens.

These findings have significant implications for nursing practice, education, and management. Nursing education must emphasise holistic language and cultural preparation before and during employment, while nursing management should prioritise ethical recruitment and transparent support to enhance both the recruitment experience and nurse well-being. Future research should explore the long-term impact of ethical recruitment certifications, the effectiveness of mentorship programs in professional integration, and the potential of digital solutions to optimise the recruitment process. Strengthening these areas would contribute to a more supportive work environment, ultimately improving healthcare delivery and the overall efficiency of the system in Finland.

## Supplemental Material

Supplemental Material - Internationally educated nurses’ experiences of recruitment to Finland from an ethical perspective: Qualitative study Supplemental Material for Internationally educated nurses’ experiences of recruitment to Finland from an ethical perspective: Qualitative study by Pauliina Oja-Lipasti, Ashlee Oikarinen, Suleiman Kamau, Sepideh Petäistö, Kristina Mikkonen, and Heli-Maria Kuivila in Nursing Ethics.

## Data Availability

All data generated during this study are included in this published article.*
