# Supplementary material for: Internationally educated nurses’ experiences of recruitment - An ethical perspective
Source: Nurs Ethics. 2025 Jun 24;33(1):188–204. doi: 10.1177/09697330251350391 (PMC12907457; doi:10.1177/09697330251350391)
Supplement: Supplemental Material - Internationally educated nurses’ experiences of recruitment to Finland from an ethical perspective: Qualitative study [file sj-pdf-1-nej-10.1177_09697330251350391.pdf]

Supplementary file 1. Progress of complete analysis

Meaning unit	Code	Sub-Category	Category	Main Category
“Then my friend recommended me this agency (1)”	Friend recommended agency (1)	Finding out about the recruitment company from a friend	Discovering the recruitment company	Preparations for recruitment
“A reference coming from a from my father's friend gave the agency in the Philippines who recruit nurses going to Finland. (15)”	I got a reference from my friend for the recruitment company (15)			
“No, actually I can't tell through, but this through somebody he was my friend in Cairo, in Egypt. He told me that he just opened some company here like agency. And he will contact with the university like that and we can put this in the we can work like that. (20)”	Hearing about the recruitment company from a friends who opened up the company (20)			
“It was given by my friend. She was also working abroad she works in Saudi and she invited me to to apply for this company. (4)”	Friend recommended agency (4)			
“The agency is actually we have a friend and our friend worked at that agency and then my wife also previously worked in that agency, so. Yeah, that's how we know about everything. (7)”	Friend working in the agency (7)			
“It was from my friend. From a friend, he was actually applied from a different agency. And then ** *** because from theres they when they applied, they weren't like nurses. They were just caregivers. And then she mentioned to me about this agency who are hiring nurses. So yeah, I tried there. And then, yeah, I proceed with that agent coming here to Finland. (21)”	I heard about the job opportunity in finland from my friends (21)			
“I saw this advertisement in Facebook. I saw an agency. And then I read there that they are hiring assistant nurses in Finland and then we are going to undergo an apprenticeship course for nursing. But before that we need to study for almost one year of Finnish language before we will become accepted. So far I passed that exam for me to come qualified, that's why I'm here now. (17)”	Finding agency trough Facebook advertisement (17)	Finding out about the recruitment company by myself		
“To be honest, I just saw it in the Facebook. Facebook advertisement before. (19)”	I found the agency from Facebook ad (19)			
“It was also in the Internet. And then I think it was on Facebook? It	I found the agency from			

was on Facebook, yeah. (14)”	Facebook (14)	
“I found out through social media, so I was already researching a couple of opportunities. Other countries as well. (5)”	I found recruitment company from social media (5)	
“I saw it in actually in Facebook. It's this ***. So I thought it was just as commerce, but it was it's true. I tried to apply it and they make interview. They made us a free language training but it takes a time before we got in here. But yeah, we have exams for language Finnish language also. So that's why we that's how we came here. (13)”	I found the agency in Facebook (13)	
“It just cross along the Facebook when I was done screening the Facebook I've seen the advertisement that there is an offer in going to Finland in a student pathway. So before I applied in agencies but. (11)”	I saw the agencys ad in Facebook (11)	
“Yeah, well, at first I've got I have read advertisement in the Facebook, such kind of agency in the Philippines that they're that they are helping Filipino people who wanted to go abroad. And one is Finland and I tried to contact them and we talked on how they can help me. So even I am a professional nursing the Philippines, I cannot. Work instantly as a professional nurse here in Finland. Not unless that I will go into study. (12)”	I saw advertisement in Facebook about working in Finland (12)	
“The agencies I found in Nepal like I was just searching to study of Finland and I was trying to apply by the self, but it's little difficult to applying the self like I'm came through the tailor made programme right? And the tailor-made programme is a little bit difficult to applying through the sales like the tailor made programme we it's connected with the some agency with college. So yeah and yeah I was searching for the some agency and I found and then I applied through this, yeah. (18)”	I found the agency by searching for it myself (18)	
“Uh, yeah, actually what happened was I was connected by my relative to one agency. But I applied, I did all the paperwork and everything, all by my own, but they were the connecting factor for me to Finland, yeah. (8)”	My relative suggested me this agency (8)	Hearing about recruitment company from a relative
“Actually umm it was my cousin who, she’s also a nurse, and our	My cousin told me about	

common friend, they applied to Sweden, but they stopped hiring. So then Finland was the country that was available to apply. Also when they opened up their requirements in the Philippines, it was quite easy to apply cause they don't need experience to apply here, also the language, even though you still need to study for it but still. (2)"	the opportunity to come to Finland (2)		
"Because I applied when I was in Philippines, so we took first we passed our these are requirements like our transcript of records, diploma and then after that, maybe after three weeks they have scheduled for interview so. (13)"	First we need to pass the requirements like the transcript of records and diploma (13)	Needing to pass requirements before coming to Finland	Initial steps in recruitment preparation
"All the proceedings that I have made I passed, I have been accepted in one of the prestigious university here in Finland, which is the *** university. They yeah, they gave me an opportunity to study there. (12)"	After I passed all the proceedings I was accepted to a university in Finland (12)		
"So I've submitted the initial requirements and then like diplomas and so on and so forth and then after a while they contacted me that I have to attend an online orientation and after that. (15)"	After submitting the requirements I attended online orientation (15)		
"I saw it in actually in Facebook. It's this ***. So I thought it was just as commerce, but it was it's true. I tried to apply it and they make interview. They made us a free language training but it takes a time before we got in here. But yeah, we have exams for language Finnish language also. So that's why we that's how we came here. (13)"	Agency has exams for Finnish language we need to take (13)	Recruitment process required taking Finnish language exam	
"Oh OK, so the recruitment it was the first thing we studied the language and then it was for 8, 8 months and then we need to take up the A2 exam. (14)"	During the recruitment process after studying the language we need to take A2 exam (14)		
"They are hiring assistant nurses in Finland and then we are going to undergo an apprenticeship course for nursing, which is lähihoitaja. But before that we need to study for almost one year of Finnish language before we will become accepted. So far I passed that exam for me to come qualified, that's why I'm here now. (17)"	I passed the language exam (17)		
"The offer before is we have to take the Finnish language for seven	Taking three exams while		

months in the Philippines and it has a 3 exams and every time the prefast an exam we were paid. (4)"	studying (4)	
"Also we have to pass the language exam, which is really difficult. I didn't know it's how the it's really difficult. It's a difficult language, honestly, and sometimes it gets frustrated because you don't. I can't easily learn fast. I can't. I don't know if I am the problem or just the language is really difficult. (15)"	We have to pass the language exam (15)	
"According to their because they have the rules and regulations also there. So I think that everything is just ethically done like there is a rule, there is a rule like if you pass the A2 then you will have the chance to apply for the visa. So everything is on process. So I think that it is done very ethically. (9)"	Need to pass a2 level of language to get a visa (9)	
"I am undergoing my process while I am working in the Philippines, working going there and here I'm getting some documents more and more documents that I need to provide for them. (12)"	Need to provide lot of documents to agency (12)	Recruitment company requiring lot of documents
"And other documents are like educational our educational backgrounds so diploma something like that. So we're able to. It's quite the processing and the submission of requirements. (15)"	Recruitment process is mostly about submission of requirements (15)	
"So there's really a lot of documentation that you need to process, especially if you are bringing family members with you. (5)"	Lot of documentation s to process (5)	
"Learning in school and doing the training in the hospital and eventually looking for a job and you know, sending out your resume and going through interviews. So it was I think that could have been improved a lot and like focused more on if they're recruiting nurses, then there should be nursing language even before you leave your home country. (5)"	Agency should offer nursing language training before coming to Finland (5)	Language should be studied first before coming to Finland
"If they gave us more professional language training like, you know, words that you really use in the work setting or in the school setting. (5)"	Professional language training should be given (5)	
"Like, universities should give classes before I came here so I will have like B1 level or something then I can I come to Finland? I study, I can work even from the first day like first time like to two	With knowing enough of language I can work from the first day (10)	

days per week or something. Then it's good for the students, good for the country, good for the university. Good for everyone. Yeah, yeah. There's a very big gap. Like for me, turn down suffering. I will start studying the language on September because the school start in. (10)"		
"Like my opinion to have professional nurses in Finland since the Finland shortage in nursing, we should learn the language before we come. Like if the if England have shortage really and Finland want to hire more experienced nurse. (10)"	Agency should require Finnish language first before coming to Finland (10)	
"From all over the world. It should be. More organised, I mean I should learn the language first. Language is everything. Like I know the job I cannot work, I cannot. (10)"	I should learn the language first before coming (10)	
"But yeah, with the language it can speed up actually your application and all. If you were able to pass with a A2 exam and I think right now they are trying to increase the level of language that Filipinos can come here. They should at least have B1 level of language. But you know, you know that we cannot have it like that very easily because. (9)"	Nurses should have B1 level of language before coming (9)	
"And what I think is like if you if you are bringing nurses like that, first of all, make them capable of speaking in Finnish and everybody should cooperate. Cooperate with the immigrants because I don't know about. (8)"	Finland should require language skills (8)	
"I can Google it yeah but it's not obligatory. But the university make it obligatory. Not in all programme, but I mean for the top up programme. So I asked, you know, persons are asking me. So I asked *** University also. They told me you have to apply through the companies. ***, they don't have the programme now, but the programme only on *** now and ***. I'm not about ***, but I think it's. I think the university is doing this also for financial perspective. (10)"	I can google all the information what the agency gave to me (10)	Finding the information for coming to Finland by myself
"Without, without any help from them, because I think only the as I keep on repeating. The only thing that I got from them is the information on where to apply and then the rest I did everything so.	Finding information without the consultancy (3)	

(3)”				
“We have interviewed just for like a basic if you have known for like numbers in Finnish and then after we passed it we they we have this language. (13)”	The interview with agency is about basic Finnish language like numbers (13)	Having an interview with recruitment company for coming to Finland		
“Which is that tie up of the agency here in the Finland, which is ***, and then they have a tie agency tie up agency in the Philippines, which is stop make international manpower services. And then I applied through top make and then I waited for an interview and after the interview I passed the interview. (19)”	After applying to the agency I did and interview which I passed (19)			
“I think in my experience with. The agent they everything just came with papers and. Yeah, documents legally and. When it comes to like thing doing what is right? I don't know. Personally, I don't really have a big issue about it, as far as I remember. (21)”	Personally I didn’t have big issues with the agency (21)	No issues with the recruitment company	Satisfaction level with the recruitment company	Experience with the recruitment company
“The recruitment process in my experience it just goes well. It's been like very smooth process and. Yeah the communication was good. It was smooth and yeah, it's been like really, it just goes like. Follows all the steps well and I have like nothing really negative to comment about it. And yeah, I was, like, very satisfied and I am really grateful by the way it runs. And yeah, that's it. (21)”	I don’t have negative comment about recruitment process (21)			
“And then when we communicate with them they were also like very understanding and flexible. (21)”	Agency was flexible with us (21)	Positive experience about operation of the recruitment company		
“And then when we communicate with them they were also like very understanding and flexible. (21)”	Agency was understanding when we communicate with them (21)			
“It was very systematic and the people who manage the. The agent was really like, very supportive and approachable, and it isn't really like they didn't require a lot of. Like. A lot of money. (21)”	People who manage the agency were supportive (21)			
“It is the agency here in Finland which is basically the ***. Two days after we arrive here for the first thing that we need to do we need to register first our self in DVV. Uh opening uh bank account, Vero and then Kela. Yeah, they teach us all of these things. Then they provide us a book. If we have	Agency provided us with a book where is answers to our questions (17)			

the question, we can read on it all in there. (17)”	
“Taught us like how to what are the rules when it comes to like renting a house, like the steps that we need to do? What are the first steps, second steps like? Doing the move from moving to Finland like, yeah, they have assisted us and they have made themselves like available. (21)”	Agency was available for us when we moved to Finland (21)
“And then they gave us the chance to. Pay. Yeah, because when we arrive here like they provide our basic materials like beds and everything and kitchen like tools and beddings and everything and they paid for it ahead of time. And then. For them, like they have like given us time to pay it slowly and cut it in portion until we can able to pay them back. When we are, when we were, when we will be. Financially stable. So yeah, I think for me that helps us a lot. Yeah. And then they didn't really like force us to like pay for this period. They gave us a due date. They gave us a due date and we tried to, like, settle it within that period. (21)”	Agency didn’t force us to pay for the household items they provided (21)
“They make it like easy for us to like, do the study in a systematic way and in in our own pace like we can choose whether we go fast or slow. It depends on how we can manage our time and study because we were also working when we do the study and then the thing that. (21)”	Agency made studying easy for us (21)
“Yes. And because also they have experience. Like they have, like, credible experience and they have been doing the recruitment for more than, I don't know, like a lot of years already. So I do feel that they are doing it in a responsible way. I do know I do know that the fee the service fee is relatively a big amount and I know that my. (5)”	Agency has multiple years of experience (5)
“Well, it's OK. It's they're based in one of the provinces in the northern part of the Philippines. I lived in the capital for so long, ever since I was born. So I was it was. I felt weird because I was sending my papers in a provincial country in the provincial part of, you know, of the Philippines and like. But it was legit, actually. So, yeah, I'm here. (16)”	Rcruitment company was ok (16)

"The instruction is very clear like you have to be you have to legalise this one you have to have this one. (3)"	Instructions from consultancy are clear (3)
"Yes, to use and then when I came here, they offered a loan so that we could start up and then we have our apartment. (4)"	Company offered a loan to startup with apartment (4)
"It was a professional. Yeah, very good. But this was really professional and they were straight to the point. (14)"	Recruitment company were straight to the point during the process (14)
"It was a professional. Yeah, very good. But this was really professional and they were straight to the point. (14)"	Recruitment process was professional (14)
"In my opinion, Finland's recruitment process excels in ethical and responsible practices. It ensures fairness, transparency, and respect, managing costs efficiently. (22)"	Finland recruitment process ensured fairness (22)
"OK well for one, what with well is that they deployed us in less than a year, so I never expected that I expected it for maybe a year or more than a year, so that went well so. (19)"	Agency arranged a job for us in less than a year (19)
"Yeah. Did they experience is it is great. It's great. The experience is great. (4)"	Experience with recruitment agency is great (4)
"They said that we can look for work on our own and you know, they showed us, like Internet portals of like, where you can find or apply for jobs and stuff like that. And they help us prepare our CV, like our resume. And like, what is the standard format in Finland because it's a different from our own, like standard stuff like that. But so it's more really like preparation that they help us with with regards to work. (5)"	Agency guiding from where we can look for work (5)
"They send us e-mail and they let us know what we need to produce, what kind of document and how to process it. And after that they're going to I am going to send them through e-mail. I will scan all the documents and send them through email and they will be the one responsible to forward it here in Finland. All we need to do is to provide all the documents needed and they are the one going to send	Agency is the one responsible of sending documents to Finland (12)

here and can and communicate here, yeah. (12)”		
“Taught us like how to what are the rules when it comes to like renting a house, like the steps that we need to do? What are the first steps, second steps like? Doing the move from moving to Finland like, yeah, they have assisted us and they have made themselves like available. (21)”	Agency assisted us in the beginning in Finland (21)	
“So yeah, everyone that I work with was professional, you know, very transparent about their business and they are transparent about. (5)”	Everyone in the agency was professional (5)	
“So yeah, everyone that I work with was professional, you know, very transparent about their business and they are transparent about. (5)”	Agency was transparent (5)	
“There's no like a middle person who will be taking the money and who will be paying the school in behalf of us. So it's directly from us to the school. So it was perfect. (7)”	No middle persons taking the money (7)	
“Send the money directly to *** account and there is no middleman when it comes to finances, so I think that's the. Yeah, I think that's the best part, because then it's very transparent where your money goes because it's really a big amount. (5)”	No middle man when it comes to finances (5)	
“You know all the payments and I think one thing that really stood out. For that agency that we used in the Philippines is that they don't accept payment. For other processing so the pay the money that you hand to the agency is only for their service fee. So like when the when the time comes that you need to pay for migri or you need to pay for the school, then you have to pay it directly. So that is one big difference because other agencies they are, yeah, other agencies they accept the money in bulk like all the money that you need to pay. And they say that they are the ones who are going to pay migri and. (5)”	Agency was transparent about payments (5)	
“Well yes because yeah, because from time from time to time. (12)”	Agency was operating responsibly (12)	Recruitment company was operating responsibly
“This company. Yes, maybe they're. Yeah, they're responsible because when we come here, yeah, we signed the contract. It's the same	I think the recruitment company was operating	

when we come here so. I think it's. Yeah, it's good. It's OK. Yeah. Nothing changed, I think yeah. (13)”	responsibly (13)
“Just have one thing that because at first I was applying a solo and then I heard that *** is on family recruitment and my other Filipino nurses who applied for the family recruitment or the final recruitment they were able to bring their family. They were able to bring their children with them. But for us because they came from I think they have different agency they are under *** but for us who came from the Philippines I we had a difficult time, so I was not able to bring my child with. I was supposed to bring him with me. Bring him with me a long hour here in Finland. But unfortunately he was not able to bring him. But he they said that in three months time we can apply for their visa. (15)”	My agency arranged only solo recruitment not family recruitment even it would have been possible (15)
“Well, it's OK. It's they're based in one of the provinces in the northern part of the Philippines. I was. I lived in the capital for so long, ever since I was born. So I was it was. I felt weird because I was sending my papers in a provincial country in the provincial part of, you know, of the Philippines and like. But it was legit, actually. So, yeah, I'm here. (16)”	Recruitment company was legit (16)
“In my opinion, Finland’s recruitment process excels in ethical and responsible practices. It ensures fairness, transparency, and respect, managing costs efficiently. Promises about salary, housing, benefits, and work environment are clearly documented and honored. Contracts align with candidates' education and qualifications, boosting job satisfaction, and include reasonable restrictions like non-compete clauses. (22)”	In my opinion finlands recruitment process excels in ethical and responsible practises (22)
“Yes. And because also they have experience. Like they have, like, credible experience and they have been doing the recruitment for more than, I don't know, like a lot of years already. So I do feel that they are doing it in a responsible way. I do know I do know that the fee the service fee is relatively a big amount and I know that my. (5)”	Recruitment company operating responsibly (5)
“I think in my experience with the	Agency

agent they everything just came with papers and. Yeah, documents legally and when it comes to like thing doing what is right? I don't know. Personally, I don't really have a big issue about it, as far as I remember. (21)"	arranged the documents legally (21)	
"I think from an ethical point of view. I think it was done ethically for for me personally I didn't encounter any unethical practises or any unethical problems. (5)"	I didn't encounter any unethical practises (5)	Recruitment company was operating ethically
"For me, everything was done ethically. Besides the family ties. (2)"	Recruitment was done ethically (2)	
"Yeah, of course. Of course. Actually. Yeah. I think everything is according to their because they have the rules and regulations also there. So I think that everything is just ethically done like there is a rule, there is a rule like if you pass the A2 then you will have the chance to apply for the visa. So everything is on process. So I think that it is done very ethically. (9)"	Recruitment company operating ethically (9)	
"Yeah, I think, yeah, I see it was ethically right there, especially that we are just alone in the process so we don't have anyone else, but we don't know. (7)"	Recruitment company working ethically because of being alone in the process (7)	
"No, no, no, we didn't pay. No, no, we didn't pay only for the ticket. That's the only thing that we pay. (1)"	No need to pay for agency fee (1)	No fee for recruitment company
"No, nothing I told just. Yeah, I had to gas myself. I have to drive this the airport of going here and not to get to get the papers to. Yeah, that's most of it. (16)"	No need to pay for the agency (16)	
"No. We pay for our own visa application. I mean, the visa application is covered by the by the employers, so we only pay for our plane tickets and our medical. (9)"	No need to pay for agency fee (9)	
"Oh OK, so the recruitment it was the first thing we studied the language. And then it was for 8 months and then we need to take up the A2 exam. And then when we arrive here, they already I mean, we already have the apartment. And then we had a contract from *** just three years. So and then the agency explained to us about like the culture here and then the housing and and and us. Yeah, basically. That's it. Yeah. OK. (14)"	Agency arranged apartment for us ready in Finland (14)	Recruitment company helped with housing in Finland

"I think it was fulfilled. I don't have really any disputes with regards to what they did to me because I was given an apartment. (16)"	I was given an apartment (16)	
"Then for the housing, I think it just. They have like made the contract initially for us and find the the most possible closest possible area that is nearby our work. And for us who like lived in that house. They have given us like one year contract (21)"	Agency arrange house for us near from our work (21)	
"Yes. So we had to pay for our own flight tickets. We had to pay for our apartment. So they partnered us the the Finnish agency was the one who introduced us to the local company here in ***. Who will, you know, where we can search apartments, even though we're we are still in the Philippines. So that was ***. So once we found an apartment and, you know, we work directly with the with the apartment agencies. So when we found the apartment, we paid directly to ***. (5)"	Agency introduces us to the housing company (5)	
"I think yes, because we have this free visa, but only our ticket and our medical we pay for it. But for me it's OK because when we arrive here in Finland they feature us in the airport. They give us free card and then they took us to our city and also in our apartment. So it's I think it's good. Yeah. Our equipment agency, yeah. (13)"	Agency was waiting us at the airport (13)	Recruitment company waiting at the airport when arriving to Finland
"To be honest, I think the one unethical or unprofessional thing that I encountered with them, although they are professional with the when we arrive here in Finland they met us in the airport and then they assist us in our temporary shelter and after that they also assist us in our apartment, but. (19)"	The agency in Finland met us in the airport after arriving (19)	
"I think yes, because we have this free visa, but only our ticket and our medical we pay for it. But for me it's OK because when we arrive here in Finland they feature us in the airport. They give us free card and then they took us to our city and also in our apartment. So it's I think it's good. (13)"	Agency took us to our city and to our apartment from the airport (13)	Recruitment company arranged transport from the airport when arriving to Finland
"Thing that I encountered with them, although they are professional with the when we arrive here in Finland they met us in the airport and then they assist us in our temporary shelter and after	The agency in Finland assisted us to our apartment after arriving (19)	

that they also assist us in our apartment. (19)”		
“It was really good because that time there's no pandemic yet, so I was able to have my language training face to face, so it's really good and the process is very smooth even with a visa and all. So everything went well. (9)”	My experience with recruitment company is good (9)	Experience with recruitment company is good
“Experiences are not bad. Not so far. (14)”	Experience with recruitment process is not bad (14)	
“Possibly recruit the student that assist with bringing us from Philippines to Finland so they assist us with the process. So I feel like I overall I had a good experience because the agency in the Philippines, they were very well versed with the documentation that was required. (5)”	Overall good experience from recruitment company (5)	
“Uh, we give regards to the updates and with regards to the information because. In my own opinion, I have realised that in order for a company to have games or to have earnings is you have to please a client or you have to please me. So what I have heard from the agency are all. (7)”	Agency is pleasing client (7)	
“Yeah, so the consultancy or agency is not aware much more knowledge about the process thing like the whole how much time to take the process like this and yeah things like we have to wait almost four month to go to this season. Yeah. It's a long and later on my many friend came through the agency and they only took only a two weeks to get a resident for me yeah and we are we need to wait four months yeah. And that's the things I think the knowledge, less knowledge maybe due to cost base and the overall is good. (18)”	Overall agency was good (18)	
“So with our I can compare it with my agency where even though I had a lot of documents it was really smooth sailing and there was nothing lacking, nothing missing. Yeah. (5)”	Nothing lacking from documents (5)	
“So everything in terms of our personal information and documents are handled pretty good. (7)”	Recruitment company working ethically due to handling personal	

	information and documents in a good way (7)	
"You know, without the help of the agency, without them, I can't. I couldn't. I couldn't be here. (12)"	Without the help from the agency I couldn't be in Finland (12)	Using recruitment company to come to Finland was helpful
"Yeah, like they did a lot of work for us through the applying. So we have to pay some amount to the agency. Yeah. Like, this is the fees are applying fees are. Yeah, we have pay some amount that's OK like that not much amount that we have paid that's. Little amount, but nowadays many of train and some of them talking about nowadays agency taking a more amount to applying the Finland. Yeah, but I'm not paid that much high. I think that's enough to pay because they help us a lot. So yeah. Yeah. (18)"	Agency did lot of work for us (18)	
"The agencies I found in Nepal like I was just searching to study of Finland and I was trying to apply by the self, but it's little difficult to applying the self like I'm came through the tailor made programme right? And the tailor-made programme is a little bit difficult to applying through the sales like the tailor made programme we it's connected with the some agency with college. So yeah and yeah I was searching for the some agency and I found and then I applied through this, yeah. (18)"	I think it's difficult to apply by myself without the help of agency (18)	
"While the Finnish agency also was very helpful to give us information about Finland, because the Finnish agency is the one who knows more about what how life is in Finland and the reality of living and working and studying. So it's also that finnish agency who is knowledgeable about it. So I feel like the teamwork between both agencies are like helpful for us. (5)"	Finnish agency was helpful (5)	
"The time that we have been departed in the Philippines they just told us that we can contact them anytime we want if we have a question, if we need help, we can contact them. But I don't think I need to contact them because we can. If there's a problem arises here. We are the one responsible to, you	After departing from Philippines agency told us we can contact them anytime if we need help (12)	Recruitment company promised to give support after arriving to Finland

know, to arrange all the solution. Yeah. So I don't think that they can mingle anymore because they are far away from here. Yeah. So I need myself to, you know, to do the solution. (12)”			
“Promises. Well, they promise that when we arrived here. We will get like we will get support from them like when we like during the. Like our early period here, they will assist us with our documents like for applying for DVV like that and they will, they have they have. (21)”	Agency promised us that we will get support from them during our early period in Finland (21)		
“It was very systematic and the people who manage the. The agent was really like, very supportive and approachable, and it isn't really like they didn't require a lot of. Like. A lot of money. (21)”	People who manage the agency were approachable (21)	Recruitment company was approachable	
“Whenever like we have questions, we can approach them and. Yeah, it's like they were. How you call this line like they it may it. (21)”	We were able to approach the agency always when we had questions (21)		
“Promises about salary, housing, benefits, and work environment are clearly documented and honored. Contracts align with candidates' education and qualifications, boosting job satisfaction, and include reasonable restrictions like non-compete clauses. (22)”	Promises about salary are clearly documented and honored (22)	Getting the promised salary	Fulfillment of Recruitment company’s promises
“Oh, OK. For the promises I think they just show us the life, how life or how is life here in Finland? How? What will be the? Benefits if we are working here, we are living here in Finland and what will be the expected salary so they do not promise that. The salary will be given to us, but they just give us an expected salary so. Well, with the salary itself they give us what is expected. And what else? Yeah, they give us what expected. They also share about some of the experiences of our of their first batch or the. Yeah. The first batch that came here in the Finland. And what else? I think, yeah, they certainly met what they have told us. (19)”	Agency met the salary they told us (19)		
“Then the salary. The salary, there's no any problem. They're paying according what is wrote into the contract. (17)”	They are paying the salary what is written in the contract (17)		

"So far they they've met the things that they have said to us. (4)"	They've met all the things they said (4)	Recruitment company met all the things they promised
"During that period and it means really a lot for us who were new here and they have kept that promise (21)"	Agency kept the promise about helping us in Finland (21)	
"Promises about salary, housing, benefits, and work environment are clearly documented and honored. Contracts align with candidates' education and qualifications, boosting job satisfaction, and include reasonable restrictions like non-compete clauses. (22)"	Promises about housing is clearly documented and honored (22)	
"Promises about salary, housing, benefits, and work environment are clearly documented and honored. Contracts align with candidates' education and qualifications, boosting job satisfaction, and include reasonable restrictions like non-compete clauses. (22)"	Promises about work environment are clearly documented and honored (22)	
"Promises about salary, housing, benefits, and work environment are clearly documented and honored. Contracts align with candidates' education and qualifications, boosting job satisfaction, and include reasonable restrictions like non-compete clauses. (22)"	Promises about benefits are clearly honored (22)	
"This company. Yes, maybe they're. Yeah, they're responsible because when we come here, yeah, we took. What? We signed the contract. It's the same when we come here so. I think it's. Yeah, it's good. It's OK. Yeah. Nothing changed, I think yeah, the one is inside there. (13)"	After I got here its all the same is written in the contract (13)	
"In our time, there is a promise for the salary and we got it the same, but actually it's higher here. The one that have been promised to us is like 1800 because the work is like hoiva-avustaja for the first time and then for the housing. No, but I've heard from the newcomers that there they were, promised of houses and blah, blah blah, they got it. But then with an extra charge. But it's not. (9)"	Recruitment company promised for salary but it was higher here than what they said (9)	Recruitment company's promised salary was higher in reality
"Oh, we actually with regards to the salary, when we came here it was much higher than the than the salary that we were expecting then the salary and I mean the then the our contract in the Philippines. So it was a little bit higher. (14)"	The salary in Finland was higher than we expected it to be (14)	
"Initial offer was low but then when	Getting a	

we got the employr when we got our employer they sent us a new contract again and then the salary is high (1)”	higher salary offer (1)		
“It in case the little here because they have considered our experiences in the Philippines also, so it's a lot it's a little higher than what I expected. (15)”	The salary is a little higher than we expected from the agency (15)		
“Yeah, yeah. Actually they are because that time it's like winter. And there is something delayed and then they inform us also through emails that just be patient, wait for the approval like this and like that. OK, go. It's OK. It was OK on my part also because I was getting married on December before coming here. So I still have time to spend with my family. Yes, that's fine. (1)”	They were informing us about delays (1)	Recruitment company was giving information about other things	Informative nature of recruitment company
“It is the agency here in Finland which is basically the ***. Two days after we arrive here for the first thing that we need to do we need to register first our self in DVV. Uh opening uh bank account, Vero and then Kela. Yeah, they teach us all of these things. Then they provide us a book. If we have the question, we can read on it all in there. (17)”	Agency was giving orientation regarding Kela (17)		
“While the Finnish agency also was very helpful to give us information about Finland, because the Finnish agency is the one who knows more about what how life is in Finland and the reality of living and working and studying. So it's also that Finnish agency who is knowledgeable about it. So I feel like the teamwork between both agencies are like helpful for us. (5)”	Finnish agency giving information about studying in Finland (5)		
“Basically, basically they just gave information about how can we find schools in Finland, how do we process visa. (3)”	Consultancy giving guidance for visa application (3)	Recruitment company was giving information for applications	
“Basically, basically they just gave information about how can we find schools in Finland, how do we process visa. (3)”	Consultancy giving guidance for applying to school (3)		
“So and then the agency explained to us about like the culture here and then the housing and us. Yeah, basically. That's it. Yeah. OK. (14)”	Agency explained us about the housing in finland (14)	Recruitment company was giving information about housing in Finland	
“How much you might be spending	Agency was		

for the like housing and then the food. And so we're expecting it. But we were not able we didn't expect that it's going to be hard for us to go to work because we don't have bike and you have to walk like 30 minutes time and we were I was shocked because in the Philippines. (15)"	transparent on how much we will spend for the housing (15)	
"Taught us like how to what are the rules when it comes to like renting a house, like the steps that we need to do? What are the first steps, second steps like? Doing the move from moving to Finland like, yeah, they have assisted us and they have made themselves like available. (21)"	Agency taught us how to rent a house in Finland (21)	
"So and then the agency explained to us about like the culture here and then the housing and us. Yeah, basically. That's it. Yeah. OK. (14)"	Agency explained us about the culture in Finland (14)	Recruitment company was giving information about culture in Finland
"While the Finnish agency also was very helpful to give us information about Finland, because the Finnish agency is the one who knows more about what how life is in Finland and the reality of living and working and studying. So it's also that Finnish agency who is knowledgeable about it. So I feel like the teamwork between both agencies are like helpful for us. (5)"	Finnish agency giving information about life in Finland (5)	
"Oh, OK. For the promises I think they just show us the life, how life or how is life here in Finland? How? What will be the? Benefits if we are working here, we are living here in Finland and what will be the expected salary so they do not promise that. The salary will be given to us, but they just give us an expected salary so. Well, with the salary itself they give us what is expected. And what else? Yeah, they give us what expected. They also share about some of the experiences of our of their first batch or the. Yeah. The first batch that came here in the Finland. And what else? I think, yeah, they certainly met what they have told us. (19)"	Agency told us how is life in Finland (19)	
"Maybe days where just two hours of school maybe we have a rest day in the middle of the week and stuff like that. So they informed us that during these short breaks we are allowed to apply for work under our	Agency informed that during short school days we can work under student	Recruitment company was giving information about working in Finland

student permit visa that there's a maximum of, I think 30 or 20 hours. I forgot it, but it's like a maximum hours that you can work under your student visa. (5)"	permit (5)	
"While the Finnish agency also was very helpful to give us information about Finland, because the Finnish agency is the one who knows more about what how life is in Finland and the reality of living and working and studying. So it's also that Finnish agency who is knowledgeable about it. So I feel like the teamwork between both agencies are like helpful for us. (5)"	Finnish agency giving information about working in Finland (5)	
"They send us e-mail and they let us know what we need to produce, what kind of document and how to process it. And after that they're going to, I am going to send them through e-mail I will scan all the documents and send them through emails and they will be the one responsible to forward it here in Finland. All we need to do is to provide, provide, provide all the documents needed and they are the one going to send here and can and communicate here, yeah. (12)"	Agency informing us by email how to process documents (12)	Recruitment company was giving information to us about the documents for the recruitment process
"They send us e-mail and they let us know what we need to produce, what kind of document and how to process it. And after that they're going to, I am going to send them through e-mail I will scan all the documents and send them through emails and they will be the one responsible to forward it here in Finland. All we need to do is to provide, provide, provide all the documents needed and they are the one going to send here and can and communicate here, yeah. (12)"	Agency informing by email what documents we need to provide for them (12)	
"Oh, OK. For the promises I think they just show us the life, how life or how is life here in Finland? How? What will be the? Benefits if we are working here, we are living here in Finland and what will be the expected salary so they do not promise that. The seller will be given to us, but they just give us an expected salary so. Well, with the salary itself they give us what is expected. And what else? Yeah, they give us what expected. They also share about some of the experiences of our of their first batch or the. Yeah. The first batch	Agency told us the expected salary in Finland (19)	Recruitment company was giving information about salary in Finland

that came here in the Finland. And what else? I think, yeah, they certainly met what they have told us. (19)”		
“Before we start the classes, they send us already the contract, the yeah. And then the all the details there is was included the salary like this and like that (1)”	Recruitment company included salary in the contract (1)	
“And like they also inform us, like roughly the amount, like what to expect if you are working as a part time worker like you know, what are the, what are the ranges in the salary and stuff like that. (5)”	Agency informing about ranges of salary (5)	
“None. I just remember that have any discussion regarding the salary? I can't remember but they just told me that, of course, compared to the salary that you have earned in here in the Philippines, well, Finland is giving you much more. Yeah, salary. Yeah. But range of the salary I don't remember that. They give me a range of salary. Yeah. (12)”	Only thing what agency promised was that the salary in Finland is higher than Philippines (12)	
“How much? You might be spending for the. Like housing, housing, and then the food. And so we're expecting it. But we were not able we we didn't expect that it's going to be hard for us to go to work because we don't have bike and you have to walk like 30 minutes time and we were I was shocked because in the Philippines. (15)”	Agency was transparent on how much we will spend on food (15)	Recruitment company giving information about finances in Finland
“Yes. But were told to bring our own pocket money to sustain our first three months without salary. (2)”	Agency told us to bring pocket money to sustain first three months without salary (2)	
“Agency explained to us first on how much should I pay for the tuition fee and that is the right the rock time that we misunderstood each other because. Before we are leaving Philippines, we need to pay but we shouldn't be. (12)”	Agency explained to me how much I need to pay for tuition fee (12)	
“It is the agency here in Finland which is basically the ***. Two days after we arrive here for the first thing that we need to do we need to register first our self in DVV. Uh opening uh bank account, Vero and then Kela. Yeah, they teach us all of these things. Then they provide us a book. If we have the question, we can read on it all in	Agency was giving orientation regarding opening bank account (17)	

there. (17)"			
"It is the agency here in Finland which is basically the ***. Two days after we arrive here for the first thing that we need to do we need to register first our self in DVV. Uh opening uh bank account, Vero and then Kela. Yeah, they teach us all of these things. Then they provide us a book. If we have the question, we can read on it all in there. (17)"	Agency was giving orientation regarding Vero (17)		
"Yeah, even if you are up a very patient person, you really will lose control because I kept asking them because there is already a deadline and going here and deadline in going to school and yet they are there, doesn't know how to do and what to do. (11)"	The consultancy didn't have knowledge about the process (11)	Recruitment company didn't have knowledge	Challenges encountered with the recruitment company
"Yeah, so the consultancy or agency is not aware much more knowledge about the process thing like the whole how much time to take the process like this and yeah things like we have to wait almost four month to go to this season. Yeah. It's a long and later on my many friend came through the agency and they only took only a two weeks to get a resident for me yeah and we are we need to wait four months yeah. And that's the things I think the knowledge, less knowledge maybe due to cost base and the overall is good. (18)"	I think the agency didn't have enough knowledge because of cost base (18)		
"Like ethical, say in the point of ethical's view like only thing is like they did not give us proper like knowledge like we are searching for the knowledge and searching a lot of searching through the tailor-made programme and the consultancy also did not give exact knowledge right like they are hiding something. Us and this is also ethical point. Yeah. Yeah. So that's the one thing I. (18)"	We are searching for the knowledge what the agency should have (18)		
"Yeah, so the consultancy or agency is not aware much more knowledge about the process thing like the whole how much time to take the process like this and yeah things like we have to wait almost four month to go to this season. Yeah. It's a long and later on my many friend came through the agency and they only took only a two weeks to get a resident for me yeah and we are we need to wait four months	Agency was not aware how long the process will take (18)		

yeah and that's the things I think the knowledge, less knowledge maybe due to cost base and the overall is good. (18)”		
“So he doesn't know that time anything. He didn't know anything that time. Where to go in Cairo. Where to apply this paper. What to do when we will come here like that. He didn't know anything. But just we try to do everything and we did it and we reached here to study. I like that only, but it was little bit difficult but it passed already. (20)”	Recruitment company didn’t know where to apply papers (20)	
“So he doesn't know that time anything. He didn't know anything that time. Where to go in Cairo. Where to apply this paper. What to do when we will come here like that. He didn't know anything. But just we try to do everything and we did it and we reached here to study. I like that only, but it was little bit difficult but it passed already. (20)”	Recruitment company didn’t know what we do when we come to Finland (20)	
“But and then like when we came to school. They were always like everything was quite OK. And then we came here. I came here on October 28th and our school was starting on March 15 or something, I guess because they didn't even know. Like there was like, vacation over here and they, like, pressured us and sent us here. (8)”	We came here 5 months before school started cause agency was pressuring to send us here (8)	Negative experience about the recruitment company
“Yeah, as I told you, it was not. It was not clear. It was not. They promised something, and they do another thing. So Misleading. (10)”	Agency was misleading (10)	
“Yeah, but I am not really sure what is the exact scope of what I have signed. (7)”	Not really knowing what I signed (7)	
“Like they are not doing any promises, but they are talking about if we when you are landing in Finland, we can help you to find the accommodations also to sourcing the job while we are applying through the agency but later when we landed there and they did not like response our message too and that's yeah. Reply the response but that what they are talking the before we are processing and yeah, that's the difference. Yeah, that they provide the response by differently, yeah. (18)”	Agency said they will help us finding housing after landing to Finland but they didn’t response to our messages (18)	
“Just took, you know, more money but. You know, to study like we pay €9500 per year. And it should be like it should be for the normal	Using agency doesn’t add value to me (10)	

people like 8500. And also can be less if you pay early or something like this. Do do you understand? Yeah, I'm in the process I did everything by myself, like in all the process, you know, for application of the visa and everything. It's say useless for me, like it doesn't add value. (10)"	
"The time that we have been departed in the Philippines they just told us that we can contact them anytime we want if we have a question, if we need help, we can contact them. But I don't think I need to contact them because we can if there's a problem arises here we are the one responsible to, you know, to arrange all the solution. Yeah. So I don't think that they can mingle anymore because they are far away from here. Yeah. So I need myself to, you know, to do the solution. (12)"	I think agency can't solve any issues for us anymore once we are already in Finland (12)
"Yeah, as I told you, it was not. It was not clear. It was not. They promised something, and they do another thing. So Misleading. (10)"	Agency was not operating clearly (10)
"Yeah. For me, I think it's useless. Yeah, like, but this was the way. Because this is, I think here. Because the universities should allow the students to apply directly. I think they build things, but for the three years and have programme but for the top up programme. We have like someone like in between the university and the students is not good with this one. He doesn't do anything for us. (10)"	Agency doesn't do anything for us (10)
"I miss you know, I have the certification and quality like certified professional healthcare quality. To and from the quality point of view, this should be. This company should be optional, not obligatory. For the student. (10)"	Using agency for coming to Finland should be optional (10)
"She used to pressure us to pay the fees because we paid the fees in I think around November and we came here in March. (8)"	The agency was pressuring us to pay the fees (8)
"But the issue is these companies, they take too much money from the students. (10)"	Agencies taking too much money from people (10)
"The ethical problem we what we have is that this guy or this friend just he promised us with everything and but unfortunately there was no	Recruitment company promised many things

written documents that time and he will promise us with so many things but when just we receive where we came Finland he just did. He told us bye bye, I don't know anybody of you study and you work everywhere. This is the basically. (20)”	but when we came to Finland they were acting like they don’t know us (20)
“Just took, you know, more money but you know, to study like we pay €9500 per year. And it should be like it should be for the normal people like 8500. And also can be less if you pay early or something like this. (10)”	Tuition fee was higher through agency (10)
“Like they are not doing any promises, but they are talking about if we when you are landing in Finland, we can help you to find the accommodations also to sourcing the job while we are applying through the agency but later when we landed there and they did not like response our message too and that's yeah. Reply the response but that what they are talking the before we are processing and yeah, that's the difference. Yeah, that they provide the response by differently, yeah. (18)”	Agency didn’t response to us anymore after arriving to Finland (18)
“The ethical problem we what we have is that this guy or this friend just he promised us with everything and but unfortunately there was no written documents that time and he will promise us with so many things but when just we receive where we came Finland he just did. He told us bye bye, I don't know anybody of you study and you work everywhere. This is the basically. (20)”	Recruitment company promised many things but there is no written promises (20)
“I think the only confusing part was that we paid extra because we didn't know that what was written in the contract or yeah, in the booklet that the agent have given us have confuses us with our end date. Yeah. But it has been settled. (21)”	Paying the rent was confusing cause we didn’t know what was written to our contract (21)
“No. Even I didn't sign any contract to them. They cater for me service and I pay for them money. So it's when. (10)”	I didn’t sign any contract with agency (10)
“Yeah for me, I think it's useless. Yeah, like, but this was the way. Because this is, I think here because the universities should allow the students to apply directly. I think they build things, but for the three	I think the agency was useless (10)

years and half programme but for the top up programme. We have someone like in between the university and the students is not good with this one. He doesn't do anything for us. (10)”		
“Oh my God. It was completely bizarre. Bizarre. Because what happened was, first of all, they lied. (8)”	Experience with agency was bizarre (8)	
“So just to person my dream and coming to here and then what's next is that the consultancy you were in I applied to doesn't have that much experience yet in assisting the applicants in going here they doesn't know how yet yeah because their expertise is in going to Australia. (11)”	The consultancy didn’t have that much of experience assisting with the application (11)	
“With regards to my journey in going to Finland because my experience was really that not good. (11)”	Recruitment process coming to Finland was not that good experience (11)	
“They are their customer service is not good. (19)”	The agencys customer service is not good (19)	
“I have flight and they told me that it's OK because you're gonna get job there and your all your studies and everything will be credited. So you don't have to study for a whole 3 1/2 years. You just have to study for 1 1/2 years. So it's OK and I have that in my mind. And then when I came here, it was completely different. I didn't get a new job. Everybody says finish, finish, finish, finish, finish and then it was. (8)”	Agency said I will get a job but I didn’t (8)	Recruitment company promised about employment which didn’t happen
“Like work just you will study for one year and two months and there's a language. It's very easy. You will find it's very easy. He promise us like that. But when we came here we found the language is actually not that much easy. It's difficult language here and we can find work easily when we will come with €1200 just will come and just we came here we didn't find job easily. It was very, very difficult to find job here, especially if you don't speak Finnish. That, yeah, that things. (20)”	Recruitment company promised that we will find work easily but we didn’t find it easily because we don’t know the language (20)	
“Actually, they promised us that during when they told us that	The consultancy	

during studies we have, we will have a job. So we stick to that. And then when we came here, it's really actually for me, it's really a bad experience. Actually, I've been always asking ***. Is Finland really for me? Yeah, because when I came, I am late, so it's already February 1 and then comes down the what's that the. Staff from *** and then they interviewed us. So from the interview everybody the next day, most of the people, most of my classmates got an e-mail that they were hired except me. Yeah. So again, I cried a lot. (11)"	promised that we will have a job during studies but I didn't get hired (11)	
"They already booked us an apartment at what was that? I forget the company. It's kind of expensive apartment. It was around €600 for two people, but just one room. So I have a roommate with me before. And then after six months, we moved to our own apartment. (4)"	Employer rented expensive apartment (4)	Recruitment company arranged expensive housing
"I think it's not that it's not that cheap. And I think because it goes from agent to agent, so we paid a little bit higher than the usual market and the me, I totally understand that because that's how the business goes and moves. (21)"	I think we paid higher rent than the market for the agency from the housing (21)	
"The only thing that I'm not sure if this is an unethical or professional, but the only thing that I doesn't want or I did not want or maybe. Yeah, a decent one that they did us to, to us, is that the rent is so high in our apartment. I'm not sure if maybe they have got or they have also portion or percentage with our apartment. But it's really high because we are living in the countryside and we are expecting that our apartment will be much lower rather than living in cities. Or in some parts that is yeah, that that is near in Helsinki. Something like that. So the rent is so high I think they doubled it. We they doubled it with our apartment. So yeah, that's the, I think an ethical aside because I know they have percentage but. But they should not have it doubled yeah, doubled the price. Maybe a percentage of 10% maybe is good enough to compensate what they done to us, but to double it is just so much too much. Yeah, too much. (19)"	I think the rent in the apartment what agency arranged for us was higher because they took high percentage of it (19)	
"I can't help like I should do social	Agency	Recruitment

media for them and marketing for nothing. They didn't do anything. So this was an issue and the for me and also you know they want to know social media like for me I'm not social media person. (10)"	wanted to use us for their social media (10)	company wanted to benefit about applicants for their social media
"Yeah, actually I contacted the manager of the consultancy where in I applied to and then because they I saw in the post and Facebook that they were encouraging applicants, actually they also used my picture to encourage students to encourage nurses in going here. So I asked I told them not to include the promises they put in the advertisement. (11)"	Consultancy used my picture for their Facebook ad for encouraging more nurses to apply to Finland (11)	
"Yeah, as I told you, it was not. It was not clear. It was not. They promised something, and they do another thing. So. Misleading. (10)"	Agency didn't keep their promises (10)	Recruitment company didn't keep their promises
"According to the contract with that website. He will provide a house and then some houses that we saw and leaving. Don't have enough on this household things, just like tables, chairs, bed. They didn't provide. We live into an apartment, which is bear you know nothing. Nothing. We bought our own chairs, our own bed. It is not, not exactly what is written the contract that they will provide it. (17)"	We didn't get the household items what agency contract promised to us (17)	
"Oh, because before we apply, they said that Finland, I'm not home anything because they said it's a very good health care system here. But when I come here now, it's like, I don't know. Because maybe when I was in Saudi Arabia working is like when I went to. It's a lot of clinic there that if I if I'm going to seek I have to just go there and then I have my choice. But here you have to call and then sometimes even you have to pay something for me so it's, I don't know. Yes, we cannot say, I don't know. Yeah, it's like they promise like that, but I'll wait again. Oh, and also. This the tax, I don't. (13)"	Agency promised that health care system in Finland is different than I realised it is (13)	
"It's around like. When we pay that in here, it's too much money in Nepal like it's double the amount in Nepal. So our parents cannot afford that every six months. And because we were promised about scholarships and everything and we didn't got it and the language was the very biggest problem. (8)"	Agency promised that we will get scholarship even though we didn't get (8)	

"First of all, they lied and they also quite didn't have like correct information about these things because I don't wanna quite take the name, but the agency was connected with other agency in Finland. (8)"	Agency lied (8)	Recruitment company was lying
"Oh my God. It was completely bizarre. Bizarre. Because what happened was, first of all, they lied. (8)"	Agency lied (8)	
"And they lied to us, saying that this is alleged programme, like a joint application. When we give the entrance examination, but it was actually not the joint application and then we researched about it and everything was like same, almost the same because at that time was COVID. So we had an online exam and everything. So that happened. (8)"	Agency lied that it's a joint application but it was not (8)	
"But we started this from March, so we were so unable to pay the fees and they told us so many lies that we will get all the like thing that a regular student gets like free lunch. We even didn't used to get free lunch in our first year. We had to pay the whole amount that those teachers pay. (8)"	Agency lied that we will get free lunch at school even though we didn't (8)	
"Yeah. For me, I think it's useless. Yeah, like, but this was the way. Because this is, I think here. Because the universities should allow the students to apply directly. I think they build things, but for the three years and have programme but for the top up programme. We have like someone like in between the university and the students is not good with this one. He doesn't do anything for us. (10)"	Agency is just between us and the school (10)	Recruitment company being between us and the school
"Well, the recruitment process as a whole is well, I have expected it to be long because the communication process is between me there there's like a middle person. So the middle person is the agency and what I have experience is between me and the agency, but I have not experienced anything between the agency and between the school. So I have it. What I have experience is that it's quite bit long. (7)"	Agency being the middle person between me and school (7)	
"Emm no I was really surprised. And I think also, there was also lack of communication, like how they were handling us. The communication was between the	Lack of communication from agency (2)	Recruitment company had problems with communication

housing company and I think between the employer. Emm I was just so surprised when we came here, it was like half of April, no we started April 22 but then the first salary was June, I had to pay the three months. So I had to pay full month of April, May and June. (2)"		
"Was answering my questions but not all of my questions for I was satisfied with the answer, but it was partially answered. I'm here so I'm still thinking. (16)"	Recruitment company was not answering to all of my questions (16)	
"Is that sometimes they have delays or they didn't? somehow update us in the recruitment process or and if also we have questions. They do the answer or they did not answer our questions. They just keep us on hanging or they just they are. (19)"	Sometimes agency didn't answer to our questions (19)	
"But when they have this important thing or important documents that they are they need then that's that. I'm the only contacted us and after that they don't have any communication at all with us again. (19)"	When agency needed something they contacted us but other than that they don't have any communicatio n (19)	
"So if we have questions, we will wait for them for how many weeks? Or maybe sometimes months before the we got a response for the agency here in Finland. (19)"	If we had questions to the agency we sometimes waited for the response for weeks or months (19)	
"The only ethical, I think or unprofessional I think that I observed with the Philippine agency that we have is that they are not that responsive. So yeah, they are not that responsive. (19)"	The Philippine agency was not that responsive (19)	
"I think the misunderstanding or lack of getting the appropriate information like what we have done because we computed what I have told you before that we should be. And then when we come here, we are very surprised that what we have paid in the Philippines is just only half. That's that is the misinformation. And yeah, I think they need to, they need to be specific on that. They told us that they are very sure that that's only the tuition fee that we need to pay. Yeah, I think they don't know the exact information, so you know most of us people going here up on	Agency didn't know the exact information regarding tuition fees (12)	Recruitment company didn't give correct information about tuition fees

arranging our document upon applying here, most of us sell parcella sell the you know the animals that they have. Yeah, that's the pain for a minute. The pain, yeah. So and eventually that misinformation that we got from them is a big surprise for us. So it's been very difficult, yes. (12)"		
"So we, we already have music and we all know that, OK, you need to pay for this only for this, including your tuition fee. This is the whole amount of the tuition fee and then when we got here, nothing long. We are very surprised that we just only paid half of the tuition fee. So it's been, yeah, it's been quite huge amount of tuition fee that we have been paid here so. I spend lots of my urgings, my savings in the Philippines, so I brought just only small amount of money and don't mess up that in case that I could not find a job. At once, or at least I have. At least I have enough money to, you know, to use in order for me to live here in Finland. (12)"	We were not informed that we still need to pay half of the tuition fee after arriving (12)	
"Like work just you will study for one year and two months and there's a language. It's very easy. You will find it's very easy. He promise us like that. But when we came here we found the language is actually not that much easy. It's difficult language here and we can find work easily when we will come with €1200 just will come and just we came here we didn't find job easily. It was very, very difficult to find job here, especially if you don't speak Finnish. That, yeah, that things. (20)"	Recruitment company promised that the language is easy but it's very difficult language (20)	Recruitment company didn't provide correct information regarding language
"I have flight and they told me that it's OK because you're gonna get job there and your all your studies and everything will be credited. So you don't have to study for a whole 3 1/2 years. You just have to study for 1 1/2 years. So it's OK and I have that in my mind. And then when I came here, it was completely different. I didn't get a new job. Everybody says finish, finish, finish, finish, finish and then it was. (8)"	Agency not informing that I need to know Finnish in order to get a job (8)	
"Like you don't need. The other one is language is not necessary. I told them I told them it is very necessary to learn the language first	Consultancy is advertising that language is not	

before you come to Finland because that's the number one thing that has been a problem. That has been my problem. When I during my yeah, the entire process. That's why they listen to me and that's why they deleted it in the advertisement. And then secondly, is there the promise that we can get a job while studying. So we have to delete that also, you don't have to promise which are not really reliable. Yeah. (11)"	necessary but I told them it's very necessary (11)	
"And yeah, the main point there is that the information is not 100%. Accurate in terms of the way how life goes here in Finland, the way how the process flows in terms of work and in terms of finding work. So. Yeah, I. yeah, you got the main point there. Unless you have another question relating to my answer. (7)"	Agency's information about life in Finland is not fully accurate (7)	Recruitment company's information was not fully accurate
"And yeah, the main point there is that the information is not 100%. Accurate in terms of the way how life goes here in Finland, the way how the process flows in terms of work and in terms of finding work. So. Yeah, I. yeah, you got the main point there. Unless you have another question relating to my answer. (7)"	Agency's information about flow of the work in Finland is not fully accurate (7)	
"And yeah, the main point there is that the information is not 100%. Accurate in terms of the way how life goes here in Finland, the way how the process flows in terms of work and in terms of finding work. So. Yeah, I. yeah, you got the main point there. Unless you have another question relating to my answer. (7)"	Agencys information about finding a job in Finland is not fully accurate (7)	
"Is that sometimes they have delays or they didn't? somehow update us in the recruitment process or and if also we have questions. They do the answer or they did not answer our questions. They just keep us on hanging or they just they are. (19)"	Sometimes agency didn't answer to our questions (19)	Recruitment company didn't give information about delays
"No. They just have to wait. They just have to wait. And so we were like hanging at the time because I already resigned from my work during the March. (4)"	Agency not informing about delays (4)	
"Feel I think like they did not provide the exact like truth like. There, first of all, they talk it's only a one to two months of process and it's took more than a six month,	I think the agency didn't provide the exact truth about length	Recruitment company didn't have correct information

yeah. Well, I give entrance exam on October and I go to resident permit on June 15th and it's more than one time. There's the missing of information needs also. (18)"	of recruitment process (18)	about recruitment process
"First of all, they lied and they also quite didn't have like correct information about these things because I don't wanna quite take the name, but the agency was connected with other agency in Finland. (8)"	Agency didn't have correct information (8)	
"Feel I think like they did not provide the exact like truth like there, first of all, they talk it's only a one to two months of process and it took more than a six month, yeah. Well, I give entrance exam on October and I go to resident permit on June 15th and it's more than one time. There's the missing of information needs also. (18)"	Agency missing of information (18)	Recruitment company had issues with providing information
"Yeah, that's thing. Yeah. And another thing is as we came through the tailor-made programme and we have to study for the four to six month for a language course and then if we pass the language then we have to you know, we have to go the like master in nursing course. Yeah. And during this period, like consultancy did not tell about that. We did not get a student discount until we did not join the bachelor. They did not tell us and housing like student housing we have the problems to apply the student housing until we did not get to the bachelors in course, yeah, that's also ethical points I think, yeah, yeah. (18)"	Agency didn't tell us that we were not eligible for student housing before going to the bachelors course (18)	
"They are just telling me that we just need to wait for the next process and there is there is no timeline like for example after three months you will have your visa processing and after eight months you will be deployed. There is no such thing as a time frame with them because they what they are, keep on they keep on telling us before is that. (19)"	Agency kept on telling us that there is no timeframe for the process (19)	
"How much you might be spending for the like housing and then the food. And so we're expecting it. But we were not able we didn't expect that it's going to be hard for us to go to work because we don't have bike and you have to walk like 30 minutes time and we were I was	We didn't know that it will be hard to travel to work (15)	

shocked because in the Philippines. (15)”			
“Yeah, that's thing. Yeah. And another thing is as we came through the tailor-made programme and we have to study for the four to six month for a language course and then if we pass the language then we have to you know, we have to go the like master in nursing course. Yeah. And during this period, like consultancy did not tell about that. We did not get a student discount until we did not join the bachelor. They did not tell us and housing like student housing we have the problems to apply the student housing until we did not get to the bachelors in course, yeah, that's also ethical points I think, yeah, yeah. (18)”	Agency didn’t tell us that we are not getting a student discount (18)”		
“I think it also help that we have a group chat because aside from agency, I think the process is easier because we were able to really get information regarding the experience of each other, because if we will rely only to the consultancy, it's like a very a very brief information. But the experiences of one another is. (3)”	Having brief information when relying on consultancy only (3)		
“They didn't actually, they didn't even send that.<br>Also, yeah, but we didn't know that. It's like, oh, the more you work here, the more taxes they got from you, so that's the thing. But yeah. And also like because in my work we have this free accommodation. And here you're the one to there is no free here. I mean, yeah, you have to pay. You have to pay the apartment. Everything you have to do it so. (13)”	Agency didn’t inform us that we need to pay taxes in Finland (13)	Recruitment company was not informing about the obligation to pay taxes in Finland	
“When we got here, however, it was, it is only the taxes that we were, they were not able to explain to us well. So we were shocked about the taxes here. (14)”	Agency was not able to explain to us well about taxes before coming to Finland (14)		
“Experience. There was no promise at all. Promise that we will give you work and this is the amount of salary you will receive and no. So there was no promises of like that. (5)”	No promises made from agency (5)	Recruitment company didn’t make promises	Promises made by Recruitment company
“There , as far as I know in my case they did not show me or they did not they did not promise me	No promises made from consultancy		

anything. (3)”	(3)	
“In our time, there is a promise for the salary and we got it the same, but actually it's higher here. The one that have been promised to us is like 1800 because the work is like hoiva-avustaja for the first time and then for the housing. No, but I've heard from the newcomers that there they were, promised of houses and blah, blah blah, they got it. But then with an extra charge. But it's not. (9)”	No promises for housing (9)	
“They didn't, they didn't like guarantee that you can find work or we will help you find work or stuff like that. So. (5)”	Agency didn’t guarantee that we will find work (5)	Recruitment company didn’t promise about employment
“So there are no promises regarding to work, so it's just mainly about school, the accommodation and the like the span on how long it will take for me to study. (7)”	No promises regards to work (7)	
“Promises. Well, they promise that when we arrived here. We will get like we will get support from them like when we like during the. Like our early period here, they will assist us with our documents like for applying for DVV like that and they will, they have they have. (21)”	Agency promised that they will assist us with our documents (21)	Recruitment company promised about other things
“In my, in my experience, the only information they said was that because our study schedule is flexible and that it's going to be a hybrid of, you know, face to face and distance learning. And this is because it's fresh out of COVID and the pandemic. So the school has adapted hybrid learning model so they have informed us ahead of time that it is not like a standard school where you come 8:00 AM and end at 5:00 PM like the whole day from Monday to Friday because in the Philippines usually that is how our universities are. So they set up expectation that we will have some. (5)”	Agency said that study schedule is flexible (5)	
“It's just that don't. Yeah, don't use like any of drugs like that. Yeah, of course. That's very, very. Yeah. But no, no, they they I think they don't have restrictions. Something like that. Yeah. But they said that we can. Yeah, we can get our family if you want. I mean, for example, I have my own family then. (13)”	Agency said we can bring our family to Finland (13)	Recruitment company promised about being able to bring family to Finland
“Sad. Because we had this special, cause I’m here in Kainuu, Sotkamo,	We were not allowed to	

and this region opened up this family tie thing in the agency, and we can supposedly bring our families, but the agency then didn't allow us. I think that was the part where it was unfair for me. The company was handling only filipinos, another company was handling people coming from other countries like Singapore, Middle east, everywhere. We were handled by different company. And when we all arrived here from different companies, others could bring their families but we didn't. That's why Im still trying to process for my husband. That's why its unfair for me. (2)"	bring our families even though agency promised that (2)			
"It was very systematic and the people who manage the agent was really like, very supportive and approachable, and it isn't really like they didn't require a lot of like a lot of money. (21)"	Recruitment process was systematic (21)	Positive experience about the recruitment process	Positive experience with the recruitment process	Experience of the recruitment process
"But overall it's OK. (1)"	Recruitment process was overall ok (1)			
"The recruitment process in my experience. It just goes well. It's been like very smooth process and. Yeah, the communication was good. It was smooth and. Yeah, it's been like really, it just goes like. Follows all the steps well and I have like nothing. Really negative to comment about it. And yeah, I was, like, very satisfied and I am really grateful by the way it runs. And yeah, that's it. (21)"	I think the recruitment process followed all the steps (21)			
"Yeah, quite satisfied. Yes. In terms of the flow. (7)"	Being satisfied with the flow of the process (7)			
"In my opinion, Finland's recruitment process excels in ethical and responsible practices. It ensures fairness, transparency, and respect, managing costs efficiently. Promises about salary, housing, benefits, and work environment are clearly documented and honored. Contracts align with candidates' education and qualifications, boosting job satisfaction, and include reasonable restrictions like non-compete clauses. (22)"	Finland's recruitment process ensures transparency (22)			
"It was really good because that time there's no pandemic yet, so I was able to have my language training face to face, so it's really	Recruitment process was smooth (9)	Recruitment process was smooth		

good and the process is very smooth even with a visa and all. So everything went well. (9)”		
“So with our I can compare it with my agency where even though I had a lot of documents it was really smooth sailing and there was nothing lacking, nothing missing. Yeah. (5)”	Smooth sailing with agency (5)	
“It went smoothly on our part because. (1)”	Recruitment process was smooth (1)	
“The recruitment process in my experience it just goes well. It's been like very smooth process and yeah, the communication was good. It was smooth and yeah, it's been like really, it just goes like follows all the steps well and I have like nothing really negative to comment about it. And yeah, I was, like, very satisfied and I am really grateful by the way it runs. And yeah, that's it. (21)”	The recruitment process in my experience was smooth (21)	
“Yeah, like they did a lot of work for us through the applying. So we have to pay some amount to the agency. Yeah. Like, this is the fees are applying fees. Yeah, we have to pay some amount that's OK like that not much amount that we have paid that's little amount, but nowadays many of them and some of them talking about nowadays agency taking a more amount to applying the Finland. Yeah, but I'm not paid that much high. I think that's enough to pay because they help us a lot. So yeah. Yeah. (18)”	Ithink the amount was enough to pay for the agency’s help (18)	Feeling satisfied about the recruitment process fee
“Uh for the financial point of view, it's mainly. In between ourselves so in terms of submitting the finances to the agency, we don't have any problem at all. (7)”	No problems with issuing the fees to the agency (7)	
“Like I personally I would rather pay a big amount and have a sense of security. (5)”	Feeling ok to pay a bigger amount for agency to have a sense of security (5)	
“You know there is a fee that we needed to pay for their services. Overall I am happy to pay for the fee because I feel like it was good services that they offered to us and I can compare it from my other classmates who have gone through the same journey but have used different agencies because it's not	Feeling happy to pay for the fee (5)	

just one agency offering the service there are multiple other agency. (5)"			
"You know there is a fee that we needed to pay for their services. Overall I am happy to pay for the fee because I feel like it was good services that they offered to us and I can compare it from my other classmates who have gone through the same journey but have used different agencies because it's not just one agency offering the service there are multiple other agency. (5)"	Feeling happy to pay for agency's services (5)		
"Sad. Because we had this special, cause I'm here in Kainuu, Sotkamo, and this region opened up this family tie thing in the agency, and we can supposedly bring our families, but the agency then didn't allow us. I think that was the part where it was unfair for me. The company was handling only filipinos, another company was handling people coming from other countries like Singapore, Middle east, everywhere. We were handled by different company. And when we all arrived here from different companies, others could bring their families but we didn't. That's why I'm still trying to process for my husband. That's why its unfair for me. (2)"	Sad experience about recruitment process (2)	Bad experience about recruitment process	Challenges during recruitment process
"Yes, yes, it's actually. There's it's not very fine. There's a rough pages that we underwent. I can say that. How can I say it? How can I start it? It's all started in the money. Yeah, I can say that I spend lots of money. In order for them to help me, I paid them. I need to pay them corresponding to the amount that they are going to ask for the client. (12)"	Recruitment process is rough (12)		
"Talking about the process and yeah, it's little bit hard, I feel it. It's little bit, not hard, but it's difficult. Yeah, like the agency is not completely out about this. The tailor-made programme and the tailor-made programme with nursing is a first ever batch that we process from our country. Yeah. And the batch is always like the testing or the like experiment. (18)"	Recruitment process was difficult because we were the first batch from our country (18)		
"Actually, everything went badly there in Egypt because this guy. He doesn't know anything because we are. We were the first group to come through this friend. (20)"	Recruitment process went badly (20)		

"Do you understand? Yeah, I'm in the process. I did everything by myself, like in all the process, you know, for application of the visa and everything. It's say useless for me, like it doesn't add value. (10)"	I did all the process myself (10)	Doing everything by yourself during recruitment process
"We're the ones who did everything. They just guided us. (3)"	Doing everything for the process ourselves but consultancy guiding (3)	
"No, no. Nothing. Yeah. Actually we're a bit disappointed because after the information that we got, then we have to do everything on our own. So, so. (3)"	Being disappointed for doing everything on your own (3)	
"OK. And I quite waited a long time for my visa and when the intern's examination was over. (8)"	I waited a long time for the visa (8)	Waiting to get the visa
"Training we have like 8 months language or no six months, but from our five months we already have a visa processing because we have we got already have our employers. So five months we wait for the processing of visa and then it took like it's all in all it's like 8 months. (13)"	We wait 5 months for the visa (13)	
"Yeah, I am. I mean, of course it's not perfect. And I think the waiting game for the visa is the one that is the most anxious part of the. It's where you have the most anxiety in the whole process, so. Like because you know, but it's out agency's hands and it's, you know, it's out of both agency's hands. And it's just in the processing of migri and these times, migri processing have been taking, like, have been ****** and taking long because of the influx, because there is really a lot. That's what they said that there's a lot of foreigners coming in for various reasons for various visas from various countries. (5)"	Waiting game for visa is anxious (5)	
"Yeah, so the consultancy or agency is not aware much more knowledge about the process thing like the whole how much time to take the process like this and yeah things like we have to wait almost four month to go to this season. Yeah. It's a long and later on my a lot of friend came through the agency and they only took only a two weeks to get a resident for me yeah and we are we need to wait four months yeah. (18)"	Waiting four months to get the visa (18)	

"I don't know. Ten months for me to fly into Finland because I have to learn the language and it took time for the visa to arrive, so it took time. (16)"	It took time for the visa to arrive (16)	
"Oh, yeah, yes, they forgot to order the nursing in their system, and then our agency booked me a ticket already to come here in Finland around February 20. But then my visa arrive late. That's why I re book again my ticket. (1)"	Rebooking flight ticket due to visa delays (1)	Delays with visa processing
"I cannot come on the first day yet because my visa has not been approved yet because of the consultants see and then that's why I beg from to the school, to give me more time to secure the visa. And then that comes when *** says no, we will refund your tuition fee. You cannot do it. So I cried a lot and it was so terrible. I get depressed. So I came to the other nation. I contacted the other nation to help me out of it. But then the other nation said. (11)"	I almost lost my student place because of the visa delays (11)	
"So that's why maybe they are new with the process in sending students and going to Finland, that's why I got the difficulty in getting the visa. So when January 16 comes with that was last year when we started the class. (11)"	The consultancy is new with the process to Finland so that's why I had difficulties with getting the visa (11)	
"Maybe if they can at least. What they call this one, at least two to three months late. I think it's OK, but if more than three months, I think it was not it's not good anymore. Oh yes, and some of them also it's like they're hopeless because maybe. (1)"	Feeling hopeless due to delays in recruitment process (1)	Delays in recruitment process
"It's like there was a delayed too much delayed. Actually, I think they took some of them, took three to four months because before they got the employer and then the agency is, like, always promising that just wait for a while like this and like that, but not of not all of them can wait because they have family to feed also. And some of them resigned to their previous work. (1)"	Financial issues due to delays of recruitment process (1)	
"Is that sometimes there have delays or they they didn't somehow update us in the recruitment process or and if also we have questions.	Sometimes agency had delays (19)	

They do the answer or they did not answer our questions. They just keep us on hanging or they just they are. (19)”				
“And it really helps because at when I'm here, yeah, we have a seven month delay. So I really did not study finnish at that time because I was really frustrated by we were already late and then when we came here. (4)”	Feeling frustrated due to delays in the process (4)			
“Is that sometimes they have delays or they didn't somehow update us in the recruitment process or and if also we have questions. They do the answer or they did not answer our questions. They just keep us on hanging or they just they are. (19)”	Sometimes agency didn’t answer to our questions (19)			
“Well, the recruitment process as a whole is well, I have expected it to be long because the communication process is between me there's like a middle person. So the middle person is the agency and what I have experience is between me and the agency, but I have not experienced anything between the agency and between the school. So I have it. What I have experience is that it's quite bit long. (7)”	Recruitment process is long (7)	Recruitment process takes time	Time consumption in the recruitment process	
“So it really consumes your not only your money, but also your time. You have to invest some time. (15)”	Rrecruitment process consumes your time (15)			
“Ten months for me to fly into Finland because I have to learn the language and it took time for the visa to arrive, so it took time. (16)”	Recruitment process took 10 months (16)	Recruitment process took months		
“Training we have like 8 months language or no six months, but from from our five months we have we already have a visa processing because we have we got already have our employers. So five months we wait for the processing of visa and then it took like it's all in all it's like 8 months. (13)”	All in all recruitment process was 8 months (13)			
“What's this consultancy? There is no age limit required. There is no limit as long as you have the money to cover the expenses. Yeah, because it's a little bit. Yeah. High. Yeah. Expensive. Especially the tuition fees. (11)”	Tuition fees were expensive (11)	Tuition fees were expensive	Tuition fees	Costs and fees associated with recruitment
“It's around like when we pay that in here, it's too much money in Nepal like it's double the amount in Nepal. So our parents cannot afford that every six months. And because we were promised about	Tuition fee is expensive (8)			

scholarships and everything and we didn't got it and the language was the very biggest problem. (8)”			
“So we, we already have music and we all know that, OK, you need to pay for this only for this, including your tuition fee. This is the whole amount of the tuition fee and then when we got here, nothing long. We are very surprised that we just only paid half of the tuition fee. So it's been, yeah, it's been quite huge amount of tuition fee that we have been paid here so. I spend lots of my urgings, my savings in the Philippines, so I brought just only small amount of money and don't mess up that in case that I could not find a job. At once, or at least I have. At least I have enough money to, you know, to use in order for me to live here in Finland. (12)”	Tuition fee was huge amount (12)		
“So the processing fee is 100,000. So I got to struggle also and producing that amount and then comes next is the paying of the tuition fees which is €4500 per semester. (11)”	After paying for the processing fee we have to pay for tuition fee (11)	Having to pay for tuition fee	
“Deny our like the agency that we pay the tuition fees. And what if *** took us so we can pay them and we can be the regular student we offer them this concept and they were like very happy to accept us like that. And now that every agency is like voice and we are just. (8)”	School accepted that we will pay directly for them (8)		
“We I have an experience of paying my tuition fee directly to the school which went, which went smoothly. So it's a bank to bank transaction so. (7)”	Paying the tuition fee directly to the school (7)		
“I mean, the visa application is covered by the employers, so we only pay for our plane tickets and our medical. (9)”	Needing to pay for our medicals (9)	Having to pay for own medicals in recruitment process	Costs associated with the recruitment process
“I think yes, because we have this free visa, but only our ticket and our medical we pay for it. But for me it's OK because when we arrive here in Finland they feature us in the airport. They give us free card and then they took us to our city and also in our apartment. So it's I think it's good.<br>Yeah. Our equipment agency, yeah. (13)”	We need to pay for our own medicals (13)		
“For the flight ticket, it is in our own cost, including the medical everything that we do on our side	We paid for our medical fees (21)		

like medical, medical fees and flight tickets. We shouldered it by ourselves. (21)”		
“Yes, we need to pay for the agency like. And of course our ticket as well. And then for our medical our ticket and then the vaccines. (14)”	We need to pay for our medical (14)	
“You have to undergo some laboratory procedures and those are expensive and they have to go to another place which is very far from our place like they always travel from ours, from where I stay from where I live. So it really consumes your not only your time, but. (15)”	I had to spend money for laboratory procedures (15)	
“And then the plane ticket as well. I paid by my own self through my own pocket. (17)”	I paid for my own plane ticket (17)	Having to pay for own flight tickets in recruitment process
“I mean, the visa application is covered by the employers, so we only pay for our plane tickets and our medical. (9)”	Needing to pay for plane tickets (9)	
“I think yes, because we have this free visa, but only our ticket and our medical we pay for it. But for me it's OK because when we arrive here in Finland they feature us in the airport. They give us free card and then they took us to our city and also in our apartment. So it's I think it's good.<br>Yeah. Our equipment agency, yeah. (13)”	We need to pay for our own flight ticket (13)	
“For the flight ticket, it is in our own cost, including the medical everything that we do on our side like medical, medical fees and flight tickets. We shouldered it by ourselves. (21)”	We paid for our flight tickets (21)	
“We had to pay for our visa and for our plane tickets. (2)”	I had to pay for flight tickets (2)	
“Yes, for the ticket, but for all the language and for the other process, no. (1)”	Having to pay only for flight ticket (1)	
“Yes, we need to pay for the agency like. And of course our ticket as well. And then for our medical our ticket and then the vaccines. (14)”	We need to pay for our flight ticket (14)	
“We have to buy the ticket for our own. So the one way ticket it came out from my pocket, but this the visa was provided it came from Finland. The actual working permit. So yeah. (16)”	We had to pay for our own flight ticket (16)	
“Yes. So we had to pay for our own flight tickets. We had to pay for our apartment. So they partnered us the the Finnish agency was the one who	Having to pay for own flight tickets (5)	

introduced us to the local company here in ***. Who will, you know, where we can search apartments, even though we're we are still in the Philippines? So that was sevas. So once we found an apartment and, you know, we work directly with the with the apartment agencies. So when we found the apartment, we paid directly to sevas. (5)"		
"In total, I think we pay almost €1200 if we converted it in euros, so all in all. Yeah for the agency, but we not only for the agency, but it's the overall expense that we have. The aeroplane ticket. The sending of documents here in Finland in the embassy. It is included there, so also our expenses in going to the visa processing area and our expenses in processing our documents in our, in our, in our self. So yeah, we pay this certain amount to be to process our application. (19)"	We pay total of 1200 euros for the recruitment process and plane tickets (19)	
"And then we also paid for the English language training. Not English. Sorry. Finnish language training. (5)"	We paid for Finnish language training (5)	Having to pay for things related to recruitment process
"We had to pay for our visa and for our plane tickets. (2)"	I had to pay for visa (2)	
"The before the application we have to pay the processing fee in the in the consultancy which is 100,000 pesos. (11)"	Before application we have to pay the processing fee (11)	Having to pay for the recruitment process
"No, there's a minimal fee, but the thing is, when you have to submit like diploma, you have to request it from your school. And so you have to pay and then they spend time and money in requesting for those documents so sometimes. (15)"	The fee for the agency is minimal (15)	
"For the actual, for example, the ***, our agency here in Finland, but they have this partnership in our in our, in my country. I mean, so even they even the *** is, was paying for it. But the agency their partner agency in my country we also pay for them a little bit but they have additional like that so we still pay. We still think for it. Yeah, it's really expensive to come here. But I think I already what's this return for me for while I'm working here? I think my cost is already returned to me, so it's yeah. (13)"	We still have to pay little bit for the agency in Philippines (13)	
"You know all the payments and I	We only pay	

think one thing that really stood out. For that agency that we used in the Philippines is that they don't accept payment for other processing so. The pay the money that you hand to the agency is only for their service fee. So like when the when the time comes that you need to pay for migri or you need to pay for the school, then you have to pay it directly. So that is one big difference because other agencies they are, yeah, other agencies they accept the money in bulk like all the money that you need to pay. And they say that they are the ones who are going to pay migri and. (5)"	service fee for the agency (5)
"You know there is a fee that we needed to pay for their services. Overall I am happy to pay for the fee because I feel like it was good services that they offered to us and I can compare it from my other classmates who have gone through the same journey but have used different agencies because it's not just one agency offering the service there are multiple other agency. (5)"	Having to pay a fee for agency (5)
"Yes, yes, it's actually. There's it's not very fine. There's a rough pages that we underwent. I can say that. How can I say it? How can I start it? It's all started in the money. Yeah, I can say that I spend lots of money. In order for them to help me, I paid them. I need to pay them corresponding to the amount that they are going to ask for the client. (12)"	In order to get agency's help I paid for them (12)
"In total, I think we pay almost €1200 if we converted it in euros, so all in all. Yeah for the agency, but we not only for the agency, but it's the overall expense that we have. The aeroplane ticket. The sending of documents here in Finland in the embassy. It is included there, so also our expenses in going to the visa processing area and our expenses in processing our documents in our, in our, in our self. So yeah, we pay this certain amount to be to process our application. (19)"	We pay total of 1200 euros for the recruitment process and plane tickets (19)
"Yes, we need to pay for the agency like. And of course our ticket as well. And then for our medical our ticket and then the vaccines. (14)"	We need to pay for agency (14)
"I pay for my agency the	I pay for the

recruitment process maybe it's all about 80,000 pesos, something like that. (17)”	agency for the recruitment process about 80000 pesos (17)		
“It was very systematic and the people who manage the agent was really like, very supportive and approachable, and it isn't really like they didn't require a lot of like a lot of money. So basically we didn't like pay any huge amount to the agency that pains us and gives us the finish lesson. (21)”	Agency didn’t require lot of money for recruitment (21)		
“I think because of capitalism, of course, if you will give information, I think you have to pay for that information. If you want the software, you will pay for the price of the software. So I think the fee that they collected is only for the information that they will give, but after that they didn't do anything. (3)”	Needing to pay for the consultancy to get information (3)		
“Everything went OK. Just we we did interview in Egypt with the the teacher of the university and. They accepted us, we did this one. We did this interview in January. They sent this one acceptance letter in March or April, I think in March, last of March then. We paid money to agency. And then we came here in October, I think 2022 years. (20)”	We paid money to the agency (20)		
“Yeah, like they did a lot of work for us through the applying. So we have to pay some amount to the agency. Yeah. Like, this is the fees are applying fees. Yeah, we have to pay some amount that's OK like that not much amount that we have paid that's little amount, but nowadays many of them and some of them talking about nowadays agency taking a more amount to applying the Finland. Yeah, but I'm not paid that much high. I think that's enough to pay because they help us a lot. So yeah. Yeah. (18)”	I think the amount was enough to pay for the agency’s help (18)		
“Yeah. Yeah. Oh, yes, we there's what they call a processing fee. I am not clearly sure what is covered with that processing fee, but since the opportunities there and the urge and we really like to go. So we didn't really think about the processing we just. (7)”	Having to pay for processing fee (7)		
“There are also other requirements that we have to pay, like the	I had to spend money for the	Having to pay for own	

vaccines. They are expensive in the Philippines. (15)”	vaccinations (15)	vaccines during recruitment process	
“Yes, we need to pay for the agency like. And of course our ticket as well. And then for our medical our ticket and then the vaccines. (14)”	We need to pay for our vaccines (14)		
“Yeah. Yeah. Oh, yes, we there's what they call a processing fee. I am not clearly sure what is covered with that processing fee, but since the opportunities there and the urge and we really like to go. So we didn't really think about the processing we just. (7)”	Not knowing what is included to the processing fee (7)	Not knowing what is included to the recruitment process fee	Challenges related to recruitment process fees
“So he told us OK, come and he took from us €8,500. I don't know till now we don't know how much he has paid to university and how much he put in his pocket. You know we don't know till now but yeah it's little bit expensive it's other but at least we came and already we have received our work already and everything is going good now no problem. (20)”	Recruitment company made us pay 8500 euros but we don’t know how much he took for himself and how much he paid for school (20)		
“For the financial. Yeah, for me it's like expensive because even they even they paid for the visa, we have this medical and also the ticket and the ticket it was really expensive. It's just really expensive but also not that fast. Yeah, we also come it's like a for example I'm from island now and I have to come in the capital city of the Philippines. So I have to take aeroplane. It's also my own cost the cost of the flight ticket. So yeah, it's really expensive for my currency in my country. (13)”	Recruitment process was expensive for me cause I have to pay for my medicals (13)	Recruitment process was expensive for having to pay for medicals	
“You have to undergo some laboratory procedures and those are expensive and they have to go to another place which is very far from our place like they always travel from ours, from where I stay from where I live. So it really consumes your not only your time, but. (15)”	I had to spend money for traveling to laboratory procedures (15)		
“Before we are leaving Philippines, we need to pay. But we shouldn't be all the travel expenses like plane ticket like taxes at the airport and in preparation for the accommodation here in Finland, we already paid for it before going here. (12)”	We shouldn’t have to pay for everything (12)	Feeling of not having to pay for everything during recruitment process	
“I hope the I hope that the next batch of. People who's gonna come here? Should not pay their tickets anymore because it's it's a big	Recruitment company should improve in a		

money and not all nurses in the Philippines can, you know, can pay for it. (16)”	way that others don’t need to pay for their own flight tickets (16)	
“If if let's say for me, because I had an experience with the the, the, the UK thing that they're gonna shoulder everything like. The ticket, the visa, they're everything. But I was questioning their agency at first that why am I buying my ticket? It's supposed to be shouldered by you guys because I I'm the one who's gonna do the the skills there. So why am I showing so they explain. Blah blah blah. The only thing that they're gonna show there is this and that. OK, then. So then it's settled. So but I think it's. (16)”	Employer should pay the ticket (16)	
“Another unreasonable is that when we came here to Finland, we have to buy our things. We have to buy our bed, we have to buy our utensils. But it was, I think, reimbursed at some point. So we have to pay this at a due date but not really upfront because we spent all of all of our money already for the tickets, so. (16)”	It’s unreasonable that we have to buy our own things for the apartment (16)	
“I do lots of, you know, the remedies that I can do, I call friends you know families. Yeah, I can say family, but my family don't know what's my situation. She didn't real because I don't want my mom and my brothers and sister to talk about me. But my, you know, my family. That's a friend there in the Philippines, so I contact them. My family here in Finland. Also like I consider him of family friend, a close friend. I contact them. Please help me. And then eventually that is good. They lend me money. Yeah. And then they told they told me that, OK, you don't need to worry for the interest because we didn't give an interest in that. Just for you to continue your study. I will lend you money. OK. Just pay us if you have enough money. If you got the job and then just return your stairs and then that's good, I mean. That is the rough time, but you know, life is not smooth. (12)”	Borrowing money from friends to pay for the tuition fee (12)	Having to borrow money for coming to Finland
“Like instantly you're independent, so it's up to you on how you manage your finances. And yeah, I	Many people borrow money to go abroad	

when I said that if a Filipino comes abroad would be like, hey, I have to borrow this big of amount of money because I will start a new life abroad. So yeah, mostly we do borrow big monies just to come here abroad specially and. (2)"	(2)	
"Sell our flat in inner province, but it doesn't come through so I beg to my families, to my relatives, to lend me some money to cover up the expenses I need. (11)"	I had to lend money from relatives to pay for the process (11)	
"I really struggled a lot, especially most especially when applying because it doesn't meet along the what's number one is the expenses, the financial matters, so. (11)"	I struggled with the expenses during recruitment process (11)	Challenges with sponsoring the recruitment process fee
"Sell our flat in inner province, but it doesn't come through so I beg to my families, to my relatives, to lend me some money to cover up the expenses I need. (11)"	I had to sell my properties to pay for the process (11)	
"The second option is that money that I brought here is that if I can find a job as early as you know, when you come here, the money that I save, I can use it to my tuition fee. But eventually we haven't had as early as what I'm thinking, so I'll spend all that. All that money that I wrote here. So the half of the tuition fee, that's the biggest problem that I've encountered here. Yeah. So I asked the help of the teachers, but eventually they told us that we need to follow the school protocol because if not, if we can, if we will give you, you know the special treatment. (12)"	I had an option to use my savings for the other half of the tuition fee (12)	
"Yes, yes, it's actually. There's it's not very fine. There's a rough pages that we underwent. I can say that. How can I say it? How can I start it? It's all started in the money. Yeah, I can say that I spend lots of money. In order for them to help me, I paid them. I need to pay them corresponding to the amount that they are going to ask for the client. (12)"	I spend lot of money for the agency (12)	Having to spend lot of money for the recruitment process
"For the financial. Yeah, for me it's like expensive because even they even they paid for the visa, we have this medical and also the ticket and the ticket it was really expensive. It's just really expensive but also not that fast. Yeah, we also come it's like a for example I'm from island now and I have to come in the	Recruitment process was expensive for me cause i have to pay for my flight ticket (13)	

capital city of the Philippines. So I have to take aeroplane. It's also my own cost the cost of the flight ticket. So yeah, it's really expensive for my currency in my country. (13)”			
“I'm getting documents I need to spend also lots of money yeah, because I need to I need to go for authentication. I need to go to the Department of Foreign Affairs. They have to do the make it, you know, authentic. And then I need also to pay for that. And. Umm, they're the one that the agency are the one who gave us an option, but which is cool, we are going to enrol but eventually. (12)”	For getting the documents to the agency I need to pay lot of money (12)		
“So he told us OK, come and he took from us €8,500. I don't know till now we don't know how much he has paid to university and how much he put in his pocket. You know we don't know till now but yeah it's little bit expensive it's other but at least we came and already we have received our work already and everything is going good now no problem. (20)”	Recruitment process is expensive (20)		
“Not really, because although at first because I was expecting I will, I will not be, I don't have to invest too much money, but honestly we put in a lot of money in our application process. So all my savings while working were I totally used it all just for the application. (15)”	I put in a lot of money for application process (15)		
“And also the medical fee is free also and they offer us a they can sponsor also the plane ticket. (4)”	Medical fee is free (4)	Recruitment company provided things related to recruitment process	Recruitment company sponsored fees
“And also the medical fee is free also and they offer us a they can sponsor also the plane ticket. (4)”	Agency offered to sponsor flight ticket (4)		
“And yeah, and they provide the all the materials that we need. Well, not really like everything we need, but those that are necessary for our study and they provided it for free so. Yeah, I think that's one of the things that we appreciate from them (21)”	Agency provided all materials necessary for our study (21)		
“And then they gave us the chance to pay. Yeah, because when we arrive here like they provide our basic materials like beds and everything and kitchen like tools and beddings and everything and they paid for it ahead of time and	Agency provided all household items for us when we arrived and we could pay	Recruitment company provided household items	

then for them, like they have like given us time to pay it slowly and cut it in portion until we can able to pay them back. When we are, when we were, when we will be financially stable. So yeah, I think for me that helps us a lot. Yeah. And then they didn't really like force us to like pay for this period. They gave us a due date they gave us a due date and we tried to, like, settle it within that period. (21)"	them back after being financially stable (21)		
"Another unreasonable is that when we came here to Finland, we have to buy our things. We have to buy our bed, we have to buy our utensils. But it was, I think, reimbursed at some point. So we have to pay this at a due date but not really upfront because we spent all of all of our money already for the tickets, so. (16)"	Paying for the things to our apartment was reimbursed (16)		
"Yeah, yeah, this this apartment, the one that we're staying right now, they furnish everything about the beds, about the washing machine like this and like this. So we didn't buy when we came here. That's it. (1)"	No need pay for any furniture when arriving (1)		
"So when he came back, when came back from September, that's the time that they bought us the the tickets. (4)"	Employer bought tickets (4)	Employee provided other things	Arrangements related to recruitment process
"No, because. The government kind of pays for the school, yeah. (2)"	Employer is paying for the school (2)		
"I saw it in actually in Facebook. It's this ***. So I thought it was just as commerce, but it was it's true. I tried to apply it and they make interview. They made us a free language training but it takes a time before we got in here. But yeah, we have exams for language Finnish language also. So that's why we that's how we came here. (13)"	Agency gave us free language training (13)	Language training was provided	
"It's more on the language. They've given us a Finnish classes every Monday. (3)"	Employer giving language classes regularly (3)		
"For the housing. It is mainly between me and the school already, so there are no problems there. (7)"	Housing arranged from the school (7)	Housing was arranged in Finland	
"I I think it's reasonable because when I came here, there's already already apartment, but yeah, I have. I have to pay for it also every month. So I have to work immediately so there. (16)"	Employer arranged apartment for me (16)		

"And then when we asked the agency about our accommodation, they told us that our employer set this thing up. So we don't have any problem about it. (1)"	Employer took care of the housing (1)			
"We have to buy the ticket for our own. So the one way ticket it came out from my pocket, but this the visa was provided it came from Finland. The actual working permit. So yeah. (16)"	Visa was provided by Finland (16)	Visa was provided		
"I think yes, because we have this free visa, but only our ticket and our medical we pay for it. But for me it's OK because when we arrive here in Finland they feature us in the airport. They give us free card and then they took us to our city and also in our apartment. So it's I think it's good.<br>Yeah. Our equipment agency, yeah. (13)"	Process was ethical cause we have free visa (13)			
"No. We pay for our own visa application. I mean, the visa application is covered by the by the employers, so we only pay for our plane tickets and our medical. (9)"	Visa fee was provided by employer (9)			
"We the for us, the company, I mean our employer, the company that we were working paid for our visa and then. (21)"	Our employer provided the visa (21)			
"Then my visa employer shouldered. (17)"	Employer paid for my visa (17)			
"Yes, I actually we have we have a language training separate from our nursing course from the with the nursing pathway course we have, it's it's of course, the language was also and another one from so there are two language classes. (15)"	I have language lessons from school and from work (15)	Having training for Finnish language	Studying and learning the language	Language learning journey
"I saw it in actually in Facebook. It's this ***. So I thought it was just as commerce, but it was it's true. I tried to apply it and they make interview. They made us a free language training but it takes a time before we got in here. But yeah, we have exams for language Finnish language also. So that's why we that's how we came here. (13)"	They made us free language training (13)			
"It was really good because that time there's no pandemic yet, so I was able to have my language training face to face, so it's really good (9)"	I was able to do my language training face to face (9)			
"And then I applied through top make and then I waited for an interview and after the interview I passed the interview. I gradually	After I passed the interview with agency I went through			

went suomen kieli course. Or the finnish course for how many months so well. (19)”	the Finnish language course for many months (19)		
“So. While we were still in the Philippines and going through the process together. There was like a chat group like on WhatsApp created for us where we could ask each other share our share our journey and we also go through Finnish language training together before coming to Finland so. (5)”	Having a group chat with others coming to Finland for language training (5)		
“Because now I'm focused in my language. (4)”	Focusing more on to language training (4)		
“And because of their providing for the studies, the language as well, I think that the most difficult part is the language because if you're going to train here for a week and we're going to use the English language, I can do the job. (2)”	Employer is providing language studies (2)		
“Especially coming to a short course because we came to a pretend nursing course, so we had we we really struggled with the language because. The learning was very fast track. We needed to learn very fast track when we arrived here in Finland, so I feel like if they gave us more professional language training like, you know, words that you really use in the work setting or in the school setting. (5)”	In Finland we received language training (5)		
“Yeah. And I think also this hospital also they are going to start with me suomen kieli course related to this one sydän- ja keuhkokeskus. But now they are in vacation as I told you so they will start I think by end of August. So maybe is this course will help me too much also. (20)”	My employer is starting a Finnish language course related to my work for me (20)		
“Because I want to focus more on learning the language, because that's really the biggest hurdle personally for me. So. (5)”	I want to focus on learning the language (5)	Focusing on learning Finnish language	
“I think it's more on the language because we don't actually have a lot of training because for the skills I think. I don't. It's not being proud but I did not need to train for basic nursing care procedures. Yeah, because I already know it. It's more on the language that they focus. Yeah. (3)”	Focusing on language training (3)		
“I think the only hard thing that I really, really struggle until now is the language, and that's what I've	Only thing I struggle with is the language	Struggling with learning Finnish	

been like trying to develop. And yeah, and the communication skills. I think that's our like weapon as a nurse and right now I am still like struggling with it because I wanted, I wanted to communicate to them and interview them as much as I could because I think it helped us, like, develop a connection with them. Yeah. So that's what I'm still like working on until now the language. Yeah. (21)"	(21)	language
"Especially coming to a short course because we came to a pretend nursing course, so we had we really struggled with the language because the learning was very fast track. We needed to learn very fast track when we arrived here in Finland, so I feel like if they gave us more professional language training like, you know, words that you really use in the work setting or in the school setting. (5)"	Struggling with language when coming to Finland (5)	
"I saw this advertisement in Facebook. I saw an agency. And then I read there that they are hiring assistant nurses in Finland and then we are going to undergo an apprenticeship course for nursing. But before that we need to study for almost one year of Finnish language before we will become accepted. So far I passed that exam for me to come qualified, that's why I'm here now. (17)"	Having to study Finnish language for one year before coming (17)	Having to study language
"Because that's the only thing that really wasn't like given. Like it, it really wasn't presented in a more professional way because like I mean, I'm professional in a sense of the language level, not the professionality of the service, but the language level because we learned just the basic A1 before to Finland and it was very basic like, you know, like trees and animals and numbers. And it's also it's like very like super basic language. (5)"	We studied A1 level of language (5)	
"Well, it was the corona time, so I was working as a private guide in nurse while I was studying Suomi kielikurssi like the A0 to A1 part of the Finnish language then. (16)"	Studying A1 level before coming to Finland (16)	
"So far I have a little bit of struggle in studying the Finnish language, because to be honest I'm still working that time the Philippines as a nurse in a port and I'm just only studying at night after I arrive in	Having to study Finnish language (17)	

my home after my long day of work. So tiring I didn't know. How did I pass that? Because I still had a lot of things that need to prioritise, just like my kids. I need to bring them to their school. In the morning and then I still need to pick up them then after that. instead of resting at night, I'm still need to study this language so far are you able to conquer it? That's why I'm here now. (17)”		
“The offer before is we have to take the Finnish language for seven months in the Philippines and it has a 3 exams and every time the prefast an exam we were paid. (4)”	Needing to study Finnish for seven months (4)	
“Oh yeah, I found out about the work from another colleague and then she said that we start as this there's no examinations, there's no ielts anymore that you need to repeat and everything, but you have to learn the language. So OK, let's give it a try. And then here we go. Let's fly.(16)”	Having to learn the language (16)	
“They told me that before I can come here in Finland I should study the language first. (1)”	Having to study Finnish before coming (1)	
“Oh, and they told us that we need to study also the sign language at the same time the Finnish language when we arrive here. (1)”	Having to study Finnish language (1)	
“I don't know. Ten months for me to fly into Finland because I have to learn the language and it took time for the visa to arrive, so it took time. (16)”	Having to learn Finnish language (16)	
“Oh, and they told us that we need to study also the sign language at the same time the Finnish language when we arrive here. (1)”	Having to study sign language (1)	
“Oh OK, so the recruitment it was the first thing we studied the language and then it was for 8, 8 months and then we need to take up the A2 exam. (14)”	During the recruitment process first we need to study the language (14)	
“Also we have to pass the language exam, which is really difficult. I didn't know it's really difficult. It's a difficult language, honestly, and sometimes it gets frustrated because you don't I can't easily learn fast. I can't. I don't know if I am the problem or just the language is really difficult. Yeah. So sometimes. Oh, I'm already four months here, but I'm not fluent. I'm	I get frustrated because I can’t learn fast and easy the language (15)	Feeling frustrated about learning Finnish language

not yet fluent. I usually don't understand what they say. Just try to if they. But I can understand some few words. And that's my like, I can get an idea from there. (15)”			
“Especially if that if, we don't talk with the real Finnish people, if we are just only studying and especially right now they are only studying online. (9)”	It’s not easy to learn the language when we dont talk with Finnish people (9)	Learning Finnish by studying it online is not easy	
“Especially if that if, we don't talk with the real Finnish people, if we are just only studying and especially right now they are only studying online. (9)”	It’s not easy to learn the language by only studying online (9)		
“Yeah, yeah. Really, like 80% in my work, especially the language because, you know, in the books or in the art language training, OK, it's. But I find that I'm more I learn more in my workplace the way they spoke to me speak to me in Finnish so much more. I learn from the from there than in the book and also the I what I'm doing there it's more better to learn. I learned from there more like 80% of is yes in my place. It's good thing. (13)”	I learn more Finnish by speaking at work than reading from the books (13)	Good experience about learning Finnish language	
“That's what my patient, they are little bit confused and on the first one to two months of my work because that's a difficulty of the communication. Just like what I said a while ago. But so far, day by day. Uh. We can understand each other because I'm studying step by step, little by little. (17)”	We can understand each other with my patients day by day because I’m learning the language little by little (17)		
“That's what. My patient, they are little bit confused. And on the first one to two months of my work because that's a difficulty of the communication. Just like what I said a while ago. But so far, day by day. Uh. We can understand each other because I'm studying step by step, little by little. (17)”	My patients are a bit confused at first because of the difficulty of communicatio n (17)	Challenges with patients due to language barrier	Challenges with the Finnish language
“Umm, there were times that, well, we have a few residents who sometimes isn't comfortable when I ask or speak in English because yeah, I think they also don't understand and I oh, and I understand it totally. And I feel very sorry and. (21)”	Some residents are not always comfortable with me speaking in English (21)		
“Umm, there were times that, well, we have a few residents who	Some residents can’t		

sometimes isn't comfortable when I ask or speak in English because yeah, I think they also don't understand and I oh, and I understand it totally. And I feel very sorry and. (21)”	understand English (21)	
“So yeah, and then at the beginning I think, yeah, we have like residents who doesn't like foreigners because we don't like they said that I don't understand what she is asking. (21)”	In the beginning our residents didn’t understand us (21)	
“So after the organisation, when we came to uh to meet to meet our boss and then she only speaks Finnish so I really didn't understand the single thing at that time. (4)”	Not understanding what boss is saying (4)	Having difficulties working in the team due to language barrier
“Yes, maybe not. Yeah, but it went well, but only the I think the difficult part only is the language and also the sign language, especially if they have a new residence and then we're not familiar with the routines like this and like that. I think that's the only problem at first. But later on we can get used to. That's it. (1)”	Not being familiar with routines due to language issues (1)	
“Enable for us to be able to become fluent with the language, but it's only a year and I was being forced to become a sairaanhoitaja, I wish very it's it has I have too much pressure right now because I don't want to drop, right. I take care of the patient because I didn't have that fluency yet. I'm not yet confident, but I have to. So I think I am if there will be a choice. (3)”	Not feeling confident about taking care of patients because of not being fluent in Finnish (3)	Feeling insecure to work due to language barrier
“Mulla ehkä se puhelimen käyttö oli, ei sen. Eniten mitä mua jännitti, että näin näin keskustelu onnistui silloin kun aloitin työt, mutta sitten puhelin puhelimeen vastaaminen on tosi vaikea kun. (6)”	For example talking in the telephone was difficult at first because of the language barrier (6)	
“That the thing that I could understand here if you don't really speak well they have kind of thinking that you don't know anything. (4)”	Feeling that people think of you don’t know anything because you don’t speak the language (4)	Feeling of not being involved at work due to language barrier
“Maybe it's on me because sometimes you feel like you're out of place because when they talking together and you cannot understand what they're saying, it's like, OK, I tend not to mingle with them when	Feeling out of place due to language barrier (1)	

they are in the kanslia during the because I don't understand what you talking. (1)”		
“Gave us the opportunity to learn more specifically in the Finnish because they gave us a free Finnish language course. Yeah, yeah. That's a big that's a big help for us. (12)”	Employer should give more opportunity to learn Finnish (12)	Employer should consider more about language
“Yeah. For example, the speaking skills like they my almost like it's a must the nurses should speak to us with English and then finish or other way around finish English. So they should always translate it to English so that we understand. And then when it comes to. Oh to the actual thing. Yeah, this. It's like a routine. So yeah, so I'm. I am now used to it already. (14)”	Our colleagues should translate Finnish to us in English (14)	
“So like people should be very much cooperative and if you have immigrant nurse, then there should be one at least one person who can speak like very fluent Finnish, very fluent English, even the Finnish. So it will it won't be much of a hassle for who does not speak. (8)”	There should be at least one English and Finnish speaking nurse at work (8)	
“Yeah. Yes, the language, the language. Umm, how they think this like everyone wanted to like for you to learn the language so fast, but sometimes it is really. It's really hard because there are so many factors like you have to live here. You have to consider the weather. You have to consider the your the demand of your patients, clients and then the your work needs also and also. (4)”	Improving the orientation by considering more language (4)	
“Mulla ehkä se puhelimen käyttö oli, ei sen. Eniten mitä mua jännitti, että näin näin keskustelu on onnistui silloin kun aloitin työt, mutta sitten puhelin puhelimeen vastaaminen on tosi vaikea kun. (6)”	Employer should consider the language more in the orientation (6)	
“It's OK. I mean, do you want think that I'm finish teachers, opettaja. Some of them are arrogant, they are not respecting the other students like that. They are forcing the students to pass immediately. This kind of task. Yeah. (17)”	Finnish language teachers are forcing students to pass immediately (17)	Negative experience about studying Finnish language
“Especially coming to a short course because we came to a pretend nursing course, so we had we really struggled with the language because the learning was	Language training in Finland was fast track (5)	

very fast track. We needed to learn very fast track when we arrived here in Finland, so I feel like if they gave us more professional language training like, you know, words that you really use in the work setting or in the school setting. (5)"		
"Yeah. And then when they are talking in our different kind of activities and assignment. Our some students don't able to manage properly to speak fluently the Finnish language, but should be accepted by these teachers because. We are still student, you know. Yeah, that's why. That's why we are studying. But the mentality of these teachers, they're thinking that we already know these things, which is very difficult, you know. (17)"	Finnish language teachers thinking that we already know these things (17)	
"Learning in school and doing the training in the hospital and eventually looking for a job and you know, sending out your resume and going through interviews. So it was I think that could have been improved a lot and like focused more on if they're recruiting nurses, then there should be nursing language even before you leave your home country. (5)"	Agency should offer nursing language training before coming to Finland (5)	Difficulties of not knowing the Finnish language
"Like my opinion to have professional nurses in Finland since the Finland shortage in nursing, we should learn the language before we come. Like if the if England have shortage really and Finland want to hire more experienced nurse. (10)"	Agency should require Finnish language first before coming to Finland (10)	
"From all over the world. It should be. More organised, I mean I should learn the language first. Language is everything. Like I know the job I cannot work, I cannot. (10)"	I should learn the language first before coming (10)	
"And what I think is like if you if you are bringing nurses like that, first of all, make them capable of speaking in Finnish and everybody should cooperate. Cooperate with the immigrants because I don't know about. (8)"	Finland should require language skills (8)	Finnish language exam is not easy
"Also we have to pass the language exam, which is really difficult. I didn't know it's how the it's really difficult. It's a difficult language, honestly, and sometimes it gets frustrated because you don't. I can't easily learn fast. I can't. I don't know if I am the problem or just the language is really difficult. Yeah. So sometimes. Oh, I'm already four	Finnish language exam is really difficult (15)	

months here, but I'm not fluent. I'm not yet fluent. I I usually don't understand what they say. Just try to if they. But I can understand some few words. And that's my like, I can get an idea from there. (15)”		
“Yes, everything with them well, not because you know the reason the language. Yes, work is nothing. I mean work is just normal for me because. What I am where I am working now is just like what I have done in the Philippines also. Yeah. So it is very familiar for me but I am having a difficulty in the language because you know. Finnish language is very difficult. (12)”	Working as a nurse is familiar for me the only challenge is the language (12)	Finnish language is the challenging part of work
“Allowed and uncomfortable to talk to these family members and give them updates about their about their about the patient or the resident. So I feel like, yeah, I'm leaning more into I'm slowly learning the role of sairaanhoitaja, which is good, but still the Finnish language is still the most challenging part everything. (5)”	Finnish language is the most challenging part at work (5)	
“See about, the orientation is OK, but the main problem is the language you know. So I feel work here is nothing related to my work in Kuwait City for 12 years. Work here is nothing. Consider nothing at all. It's very, very easy and there is no shortage in nursing as they are getting here. But I feel with nothing because of language, still not that much strong with me. Though because I came to Finland maybe one year and a half, I can feel and and throw this one year and a half. I was studying nursing in English and Savonia and during this through period I was working kotihoito. So what I was talking there was asukas it was it is different now when I'm talking with potilas here in yeah. So. (20)”	Work here is easy because of my experience but learning Finnish is difficult (20)	
“I'm trying my best to improve my Finnish language skills, because that's only the key to communicate with them. You cannot do anything if you didn't know how to speak Finnish properly. Uh, I didn't see that and so fluent, but so far. I can say something to them even that's a basic language only. (17)”	I’m trying my best to improve my Finnish language skills (17)	
“I think it's more on the language	No need to	

because we don't actually have a lot of training because for the skills I think. I don't it's not being proud. But I did not need to train for basic nursing care procedures. Yeah, because I already know it. It's more on the language that they focus. Yeah. (3)”	train for basic nursing skills (3)
“The system? Yeah, they still mess it in the. Oh, they want me to talk more so that they can push my finish skills more my speaking skills more because it's really important because I've been here for three years only, and sometimes I commit mistakes in speaking and listening. So they push me more to speak, to speak, to practise, speak with your coworker. Yeah. So that you will learn more. OK, then I said yes. I think here in Finland the most important thing that I need to learn is to master the the speaking skills and you know. (16)”	Most important thing is to learn the language (16)
“It's my problem, not their problem. I am not speaking like in the harjoittelu I did quote and they gave me like excellent feedback. But the language the right, the it could be an excellent colleague for the language, but after improving the language so the language is everything because you know the people here in Finland, they don't speak only Finnish and Swedish. (10)”	Language is everything in Finland cause here they only speak Finnish and Swedish (10)
“Regularly I mean when it comes to meetings, it's like every week and like I'll say, there's a demonstration from our PT. Like that therapist, yes, and yeah. And because of their providing for the studies, the language as well, I think that the most difficult part is the language because if you're going to train here for a week and we're going to use the English language, I can do the job. (2)”	Finnish language is the most difficult part (2)
“For the routine job yeah, it's OK because it's always a routine and we have rest. Permanent residents like 15 of them and the same routine every day. So it's easy for us and it's not difficult with because we will only assist them on changing clothes, like changing diapers and feeding like this. And like that. It's not like in the hospital you have that there is a lot of tasks to do. Yes. It's OK for us. The only the I think	Communication being the hardest thing at work (1)

the hard part only is the communication. (1)”		
“Challenging part. Yeah, that's not one thing that they want me to develop, but overall I can insert things. I can do this. I can do that, they know it. But yeah, just the language more practise. (16)”	Codes\\Finnish language is the most challenging part at work (16)	
“Yes, everything with them well because you know the reason the language. Yes, work is nothing. I mean work is just normal for me because what I am where I am working now is just like what I have done in the Philippines also. Yeah. So it is very familiar for me but I am having a difficulty in the language because you know Finnish language is very difficult. (12)”	Finnish language is difficult (12)	Finnish language is not easy
“For when it came here, it was like of course because of I mean. It's very new for me. It's like it's really, I think is the first thing for me that makes me shock that we did a study for Finnish language but in reality when they speak it's really very fast. It's not because in our studies like a very like allocates. OK, I can't do it now. But when I in reality, it's very like, you know, they speak a lot and then it's very. (13)”	Even though we studied Finnish language before coming its really different in reality (13)	
“Oh yes, because you know, it's for, of course, it's hard language and sometimes the teachers there are, even if it's free language but sometimes the teachers there are, they interview one-on-one and then by finishing language and then we have some difficulties, of course, because I work there while working I have to study at night in language training. So that's just like that but. (13)”	Finnish language is hard (13)	
“Yeah, it was really tough because they like us to speak in Finnish. We know that Finnish is a very difficult language. Yeah. So I'm just so I still speak not good. Not that good, because it's, you know, it's really hard to learn the language. So during the interview I was hard because there are four who are there. (11)”	The job interview was hard cause it’s hard to learn the Finnish language (11)	
“Sometimes I would my colleagues and I would talk about things like that, and there are some police that felt that they were so low that they would cry because they really tried to, you know. Try to learn and try to	Finnish langage is hard cause it’s not our language (4)	

speak and try to understand, but it's really hard because it's well, it's not our language. (4)"				
"Then yeah, then I'm working now, but my language is not that good. I can manage at work, but not professional of course. (10)"	My Finnish language is not good (10)			
"Also we have to pass the language exam, which is really difficult. I didn't know it's how the it's really difficult. It's a difficult language, honestly, and sometimes it gets frustrated because you don't. I can't easily learn fast. I can't. I don't know if I am the problem or just the language is really difficult. Yeah. So sometimes. Oh, I'm already four months here, but I'm not fluent. I'm not yet fluent. I I usually don't understand what they say. Just try to if they. But I can understand some few words. And that's my like, I can get an idea from there. (15)"	Finnish language is difficult (15)			
"And like those things happen. So it was not quite like a nice experience for me, like other student might have. And then I was all alone. (8)"	Coming to Finland was not a nice experience (8)	Negative experience about coming to Finland	Negative experiences as a foreigner in Finland	Cultural and workplace integration
"Actually, emm, I think because umm, the country is a bit, how do I say this, more open to foreigners, and I think when I heard their culture is quite, they are happy people. And also the people are kind and they umm they think of us as equal. Cause I have experience for two years and as you can see they are like rich people and for someone like us, like who provide services for them, they treat us like we pay for your services so you should do your job. But here its different. The culture is what I was looking forward to. But sometimes it can be quite sad here. The tendency is because if you do not or you stop to this kind of place where it's like really remote. (2)"	Sometimes it's sad in Finland because it's quiet (2)			
"Then it was such a horrible experience for me at the 1st and then coming here like. (8)"	It was a horrible experience (8)			
"I am already getting adapted to the country to the people here, to the society, to the what's that. Though there are more there are also finns who are racist. We cannot, we cannot fight death. Yeah. And then there are also fee and that that are bullish. We cannot do anything. It's really it's really hard to. Yeah, it is	We cannot do anything for the racism (11)	Experiencing racism in Finland		

all over the world now. So but then. I'm already enjoying here in Finland. Actually I'm about to get my husband already and my poika, yeah. (11)”				
“Ohh OK. Actually, because this one. I don't want to be negative. But uh, you know, some people are very nice there. There's racist people also. You know, there is percentage. Yeah, I have to be clear with this one that there's people they don't like. (10)”	There is racist people also (10)			
“Others, but especially people, are very racist in Finland. I'm so sorry. (8)”	People in Finland are racist (8)			
“And what I think is like if you if you are bringing nurses like that, first of all, make them capable of speaking in Finnish and everybody should cooperate. Cooperate with the immigrants because I don't know about. (8)”	Finnish people should be more cooperative with immigrants (8)	Finnish people should have better attitude to foreigners		
“Not everyone should go to Helsinki. The shortage in nursing in all Finland, not in Helsinki. The cultural diversity should be improved, I mean should be. The people should accept the other cultures. (10)”	People should accept other cultures in Finland (10)			
“Oon käynyt koulutuksissa ja tehnyt töitä niin kyllä sitten tuntee siellä. Mä oon oppinut tosi paljon kaikkia ja sitten siirryin nyt keskussairaalan kuntoutusosastolla 3 vuotta sitten niin. (6)”	Employer has educated me (6)	Receiving nursing related training at work from employer	Employer-organized training and orientation at work	Employer-Provided support and resources
“And you like, you have your own login and there's some required courses that you need to pass. And then there's other, like refresh, like refreshment courses if you want, like a refresher on maybe like or if you want a refresher on, you know, some other things. But then there's a specific course for, like, the basic information about. (5)”	Getting refreshment courses (5)			
“Yeah. And also communicating with residents, relatives. Yeah. And what else? Yeah. And yeah, my supervisor has been also like training me to how to handle my own residents to be, to be like the omahoitaja like that. (21)”	Employer has trained me to handle my own residents as omahoitaja (21)			
“The hygiene policy. Lääkeluvat. That's all. That's all, because I'm only new. (17)”	I have received training for hygiene policy (17)			
“Colour tooth, even in online and in personal right, so they help us to	Employer is arranging			

develop our skills by doing the koulutus and at the same time we do it on our own with the patients. We apply it like we are hands on in everything so. (9)”	trainings (9)	
“Aside from the new trainings I have received for the sairaanhoitaja role. (5)”	I have received training at work for my nursing role (5)	
“Yeah, the first fire safety training. And yeah, fire safety training. Aside from the medicine training. (21)”	I received fire safety training (21)	Receiving fire safety training at work from employer
“We have this fire training and like our first aid training. Yeah, we have done it like that in my work base, yeah. (13)”	I have received fire training at work (13)	
“Fire training. So like someone came from the fire department and the fire company to like inform us about, you know, the standard procedures. And we also did some actual training of putting out the fire, like, stuff like that. (5)”	Received fire training at work (5)	
“Training, you know I attend like 3 training sessions regarding like manual handling and ergonomics and also one day for the basic life support. And then also about the you know the medication licence. (10)”	Training for medication license (10)	Receiving medication training at work from employer
“The hygiene policy. Lääkeluvat. That's all. That's all, because I'm only new. (17)”	I have received training for medication license (17)	
“And before passing the medication exam, I didn't, you know, I didn't handle medicines and I didn't handle like communications with family members and stuff like that. So it's really more just day-to-day bedside care of the resident or the patient. But now that I you know I'm moving up to a slightly higher role of sairaanhoitaja so I have been trained in all the medications and how to give the medications and office work like reading the. (5)”	Been trained at work for medications (5)	
“Yeah, I think how to manage or do the medicine managing medicines here. Because yeah, it's like different from the way we used to before, like here we need to, like, take the medicine exam. And there were different kinds. There were lääkelupa. And then there's also, like, for injections and doing the	Employer has arranged medicine management training for us (21)	

insulin you have to do different like demonstrations. So you could have this permission to give this medicine, and I think apparently that's the that's the period where we we've been trained we were being trained. (21)"		
"Training, you know I attend like 3 training sessions regarding like manual handling and ergonomics and also one day for the basic life support. And then also about the you know the medication licence. (10)"	Training for ergonomics (10)	Receiving ergonomics training at work from employer
"Provided us a training for ergonomia I say it is a proper positioning for patients moving patients. Which is also beneficial to us as assistant nurses, so it will not be will not hurt our backs. And then it would be easy for us to lift them or move them the patients. So Friday give they have given us a training for that. (15)"	I have received ergonomia training from work to help with our nurse assistant job (15)	
"And they give me refreshment? IV cannulation you do refreshment. What else? IV training, nestehoito, I forgot it in English it I'm trying to let go of English now. (16)"	Work is training me for nursing skills (16)	Receiving skills training at work from employer
"Yeah, I think how to manage or do the medicine managing medicines here. Because yeah, it's like different from the way we used to before, like here we need to, like, take the medicine exam. And there were different kinds. There were lääkelupa. And then there's also, like, for injections and doing the insulin you have to do different like demonstrations. So you could have this permission to give this medicine, and I think apparently that's the that's the period where we we've been trained we were being trained. (21)"	Employer had trained us for giving injections (21)	
"For us to help you suction. Mounted suction and the device that you can bring to other rooms. (7)"	Training for suction devices (7)	
"Blood tests haemoglobin how to check your own haemoglobin and the patient's haemoglobin. (7)"	Training for how to take blood tests (7)	
"Test how to take a PLV, how to. Actually this simulations are all in one day and it happened a few months ago where there is an old warehouse that have converted it into a training facility. So there are different rooms where we need to enter as a group. So yeah, rooms	Training how to take PLV (7)	

are divided into different modules like what I've said they have CPR and the AED they have a section. (7)”		
“We have trainings in lab skills like there are certain modules like CPR. (7)”	Training in lab skills (7)	
“We have trainings in lab skills like there are certain modules like CPR. (7)”	CPR training (7)	Receiving first aid training at work from employer
“Test how to take a PLV, how to. Actually this simulations are all in one day and it happened a few months ago where there is an old warehouse that have converted it into a training facility. So there are different rooms where we need to enter as a group. So yeah, rooms are divided into different modules like what I've said they have CPR and the AED they have a section. (7)”	AED training (7)	
“Training, you know I attend like 3 training sessions regarding like manual handling and ergonomics and also one day for the basic life support. And then also about the you know the medication licence. (10)”	Training for basic life support (10)	
“Test how to take a PLV, how to. Actually this simulations are all in one day and it happened a few months ago where there is an old warehouse that have converted it into a training facility. So there are different rooms where we need to enter as a group. So yeah, rooms are divided into different modules like what I've said they have CPR and the AED they have a section. (7)”	CPR training (7)	
“We have this fire training and like our first aid training. Yeah, we have done it like that in my work base, yeah. (13)”	I have received first aid training at work (13)	
“Hmm. CPR training and I think it's more on the language because we don't actually have a lot of training because for the skills I think I don't. It's not being proud. But I did not need to train for. basic nursing care procedures. Yeah, because I already know it. It's more on the language that they focus. Yeah. (3)”	CPR training (3)	
“If they have, if the Apollo have some updates that we need to look into, and if we need to document more so. (7)”	Training for patient documentation (7)	Receiving patient documentation training at work from employer
“And then there is another one, which is the Apotti system because	Work place is training me for	

in the *** but it's a whole it's a differen. So they had they schooled me there. They they trained me how to use this. There's an actual Apotti manager from the hospital who came in face to face with me. While I'm yeah and then oh, you do this, you do that, you do this, you do that. OK, now I'm learning and then you click this, you click it. OK. (16)"	patient documentation system (16)	
"And then when we came to work my ohjaaja she instructed me in English and my boss said one step at a time and then. It's quite OK for me. (4)"	Personal perceptor instructing in English (4)	Receiving orientation in English
"It was ohjaaja gave the orientation to me. Yeah, and he asked she assigned me every day to a nurse who will be my own ohjaaja who can speak, you know writing my like English and finish so we can understand each other. She can explain to me. (12)"	I was assigned to another nurse who can speak English to orient me (12)	
"It was ohjaaja gave the orientation to me. Yeah, and he asked. She assigned me every day to a nurse who will be my own. Yeah, yeah. Who can speak, you know writing my like english and finish so we can understand each other. She can explain to me. Yeah, she's the one who assigning patient to me. (12)"	My perceptor gave the orientation for me (12)	Receiving orientation at work from perceptor
"Yeah, sure. Now I am the orientation period, actually it was for official organisation for two weeks. I was with ohjaaja. Then after that I started to take patients responsible for those patients from A-Z. I'm just writing also for them everything and yeah orientation is doing going now. (20)"	My orientation was for two weeks with my preceptor (20)	
"My supervisor and ohjaaja we came here. We have this. Ohjaaja. So she's the one but because our schedule sometimes we are not the same. So usually if there's like working finish there so you you can just come to them and then yeah, that's it. But mostly my ohjaaja, we have ohjaaja in my workplace. (13)"	Mostly my perceptor gave the orientation (13)	
"On my first day, it was our boss. The manager or how you call the. You're the head of the company. And on the, on the on my second day, I was endorsed to my ohjaaja. So since then I have worked with her with my ohjaaja until the end of my school. Until the end of my	I received orientation from my perceptor (21)	

school. Yeah. (21)”		
“Like she overlooks everything and she instructs my ohjaaja. So I have ohjaaja. Aside from her. And he takes he he makes all the decisions decisions and also decides what steps should I take next? After a day or after a week after orientation? Actually the orientation lasted just a week. (7)”	Having a personal preceptor for orientation(7)	
“It's my pomo and my ohjaaja get down to there's one person that is assigned for me. It's my ohjaaja. Everyday I'm together with her before I do something that's I need to. Ask her first. I have that question I need to ask her. Then she she's there to supervise me. If I didn't know something, I'm free to ask question to her. (17)”	Orientation was given by my preceptor (17)	
“Our the first day our boss gave me the basic the very general orientation and then after that I've had my ohjaaja. (9)”	I had my own perceptor giving me orientation (9)	
“Orientation lasts for three months (1)”	Orientation lasted for 3 months (1)	Orientation at work was long
“Yeah I think I was for I was like, I was trained for three months by a practical nurse, and yeah, practical nurses. And when they ask what what's your job in the Philippines? And I was like, yeah, I'm a nurse in the Philippines. They're going to ask for how long. When I said that for almost 10 or 12 years, they're like. (2)”	Orientation lasted for three months (2)	
“Is about like the more than almost one hour and the first day I have been giving, I have been taking the all the orientations as but the actual formal orientations is half to one hour. Yeah. From one department. Yeah. (18)”	The actual formal orientation was half to one hour (18)	Orientation at work was short
“I think it was very difficult or I think it was very different from the keikkari orientation to the full time position orientation. Because when I started as a keikkari 10 months ago, there was really no there was like, very just one day of training and then the next day you are are alone on your own and there's. I barely no training at all. (5)”	As a part timer I only had one day of orientation (5)	
“For the routine job. Yeah, it's OK because it's always a routine and we have rest. Permanent residents like 15 of them and the same routine every day. So it's easy for us and it's not difficult with because we will	Orientation being sufficient for routine job (1)	Orientation at work was sufficient

only assist them on changing clothes, like changing diapers and feeding like this. (1)”		
“Yes, it is. Yes, sufficient. (14)”	Orientation is sufficient (14)	
“Yeah, I think so. They've given enough time because I think for the whole year it's more on, sorry, more on practising the. (3)”	Orientation being sufficient (3)	
“So I think the yeah, it's very sufficient. (5)”	Daily exercise was sufficient (5)	
“Yeah. It's especially since I'm already. Do you understand? I already learned about the skills before, so it's like a refresher for me. So it's not, it's not new anymore. (7)”	Orientation was sufficient (7)	
“Yeah, I think so. It's sufficient enough that I'll I can learn something for my every day and, but sometimes it's not. Sometimes I just do lähihoitaja, I work sometimes I do sairaanhoitaja. I work. It's OK. I learned the things that some perish. So I learn from them. Everybody with their I can learn. I can. I know. (16)”	Daily exercise is sufficient (16)	
“In my current job. Yes, in my current job. Yeah. Yes. I think it was been enough. (21)”	Daily training was enough for my job (21)	
“That. Oh yeah, for the. Oh, at first, yes, it's not enough, but for I'm staying almost all now I become one year. I think it's enough for me. My training, I think because I mean, I knew how to give medicines like that for them. That's yeah. (13)”	At first my orientation was not enough for me (13)	
“Ihan normaali. (6)”	Orientation was normal (6)	Satisfied with orientation at work
“I've had a very good orientation, but the time is so fast like. (9)”	Orientation was fast (9)	
“I've had a very good orientation, but the time is so fast like. (9)”	Orientation was good (9)	
“Like the part now if I work as the Home Care nurse.That's good. Not enough. But that's good. Yeah. Experience training is not enough. I was, but that's a good yeah. (18)”	Experience training at work was good (18)	
“The orientation is really helpful. (15)”	The orientation at work was helpful (15)	
“See about, the orientation is OK, but the main problem is the language you know. So I feel work here is nothing related to my work	The orientation is ok but the main problem	

in Kuwait City for 12 years. Work here is nothing. Consider nothing at all. It's very, very easy and there is no shortage in nursing as they are getting here but I feel with nothing because of language, still not that much strong with me. Though because I came to Finland maybe one year and a half, I can feel and and throw this one year and a half. I was studying nursing in English in *** and during this through period I was working kotihoito. So what I was talking there was asukas it was it is different now when I'm talking with potilas here in Yeah. So. (20)"	is the language (20)	
"Like she overlooks everything and she instructs my ohjaaja. So I have ohjaaja. Aside from her. And he takes he he makes all the decisions decisions and also decides what steps should I take next? After a day or after a week after orientation? Actually the orientation lasted just a week. (7)"	Orientation lasted for a week (7)	Orientation at work was medium length
"Yeah, sure. Now I am the orientation period, actually it was for official organisation for two weeks. I was with ohjaaja. Then after that I started to take patients responsible for those patients from A-Z. I'm just writing also for them everything and yeah orientation is doing going now. (20)"	My orientation was for two weeks with my preceptor (20)	
"So the sairaanhoitaja I had there gave me the orientation. (8)"	The nurse gave me orientation (8)	Receiving orientation at work from another nurse
"At work, it was the first one the orientation, it was the sairaanhoitaja yeah. Because the our woman was not there yet. Yeah. (14)"	Nurse in our workplace gave the orientation for us (14)	
"How do I call the actually nurses in ***. But they are the ones who are in charge of us. Like if we have problems, we have concerns in our workplace and then they coordinate everything to the supervisors in our office, in the facility or the kotihoito. But they are there they were the ones who gave us the orientation and visit us all that sometimes in the workplace to get to know how we are. Yeah, OK. In the workplace, how are we doing something like that? (15)"	Colleagues oriented me at work (15)	
"And then afterwards you are partnered with a nurse and it's like you shadow the responsibility. So like an actual nurse working day.	Shadowing with another nurse during orientation (5)	

And like you shadow. (5)”		
“Yeah I think I was for I was like, I was trained for three months by a practical nurse, and yeah, practical nurses. And when they ask what what's your job in the Philippines? And I was like, yeah, I'm a nurse in the Philippines. They're going to ask for how long. When I said that for almost 10 or 12 years, they're like. (2)”	I received orientation from practical nurse (2)	
“At my workplace and the department boss gave me an orientation and another senior staff gave me the yeah orientation, yeah. (18)”	My senior nurse gave me orientation (18)	
“Our the first day our boss gave me the basic the very general orientation and then after that I've had my ohjaaja, no, it's not actually. Oh, yeah, I had better ohjaaja. (9)”	Supervisor gave basic orientation for one day (9)	Receiving orientation at work from supervisor
“It's my pomo and my ohjaaja get down to there's one person that is assigned for me. It's my ohjaaja. Everyday I'm together with her before I do something that's I need to. Ask her first. I have that question I need to ask her. Then she she's there to supervise me. If I didn't know something, I'm free to ask question to her. (17)”	Orientation was given by my supervisor (17)	
“No, it was actually our supervisor. (2)”	Supervisor gave orientation (2)	
“The orientation at work, it's the government in ***. So they have given us the summit orientation also. (15)”	Employer giving orientation (15)	
“Orientation. I think it's apulaisosastonhoitaja. Yeah, she gave the orientation for my first day. I think we're like 30 and our batch started. Yeah, on that orientation. So yeah it's like a classroom. (16)”	Assistant supervisor giving the orientation (16)	
“When I had my trainings, the school prepared really well and also the supervisors supported me a lot. Orientations were given by the school and supervisors, they also help me with the career plan, I improved everyday. At that time I was in a quite big place so I had never seen the employer. (22)”	Orientation was given by school and supervisors (22)	
“My supervisor and ohjaaja we came here. We have this. Ohjaaja. So she's the one but because our schedule sometimes we are not the same. So usually if there's like working finish there so you you can	My supervisor gave the orientation (13)	

just come to them and then yeah, that's it. But mostly my ohjaaja, we have ohjaaja in my workplace. (13)”			
“Ah yes so it's the supervisor or that. So there's just one supervised in the house. So she gives like, the orientation on the first day and then. (5)”	Supervisor giving the orientation for one day (5)		
“There that day, she was on her vapaa, but the osastonhoitaja was the so yeah, she was the one who gave the orientation. (8)”	Supervisor giving orientation (8)		
“On my first day, it was our boss. The manager or how you call the. You're the head of the company. And on the, on the on my second day, I was endorsed to my ohjaaja. So since then I have worked with her with my ohjaaja until the end of my school. Until the end of my school. Yeah. (21)”	On my first day I received orientation from my boss (21)		
“At my workplace and the department boss gave me an orientation and another senior staff gave me the yeah orientation, yeah. (18)”	My department boss gave me orientation (18)		
“Like an employee I only feel about the I have a language barriers, so they are providing me or interests to the language. Yeah. And there is little difficult to understand me but later like after a day or week day also provide me or orientations like English. So that's a little bit difficult for a few days. And yeah, that's when but the I have to improve my Finnish language. I cannot always stay with my English too, so that's my weak point too, yeah. (18)”	I have language barrier during orientation (18)	Challenges during orientation due to language	Negative experiences with workplace orientation
“This is maybe I'm not prepared my body was not prepared but information and some and the training also. Just that. This my how do I call this? It's not, is it? Ohjaaja. It's supervisor I think. There's a little concern because. Sometimes you speak fast and I was just new to the I don't know how things goes and I can't understand. I'm so confused. What are we doing? What is the flow of the ward? So at first I was really like I was really overwhelmed and I don't know what. (15)”	During orientation I was confused cause my perceptor was speaking for fast so I can’t understand (15)		
“During the orientation it's difficult to say because you're really your orientation. We conducted it in a Finnish language and I barely understand much, I think it's only	In orientation I was learning by observing because of the language		

like the very basic and yeah, I struggle during that time because everyone just like, I mean, not everyone, but most just doesn't speak like or explain the whole thing to me in English. So yeah, it's like I mostly used observation and then. If, like I could sneak, sometimes I use the Google Translate to so I could understand. (21)”	barrier (21)	
“Maybe I'm confused orientation in the work place. I think it should be first thought in English and then afterwards. Because we're new, sometimes we don't understand. Although she speaks English all the time, but there are times when she thought that maybe I understand it already. Well, and then she would speak Finnish but really I get confused. I got confused. So I think I suggest that at first they should speak they should teach us in english first. (15)”	Orientation was hard because it was in finnish (15)	
“Yeah, if I little very little, I am getting adapted to so but and discovering different things, yeah, by myself and then maybe, maybe tomorrow I will be starting as lääkeluvat. So I still don't know. What to do? I still don't know what are the dos and donts? I still don't know what are the responsibilities because as I said, there is no orientation, but maybe I will just orient myself tomorrow. (11)”	I don’t know what are the responsabilities at my work place because I didn’t have orientation (11)	Not receiving orientation at work
“It was a shock for me at that time because there was really no support and I guess the reasoning for it is because you are nolla contract, then you can choose to not come back anytime or whatever. So it it's really like they don't invest in training for those who are in nolla contract and I guess for me personally because I needed the job or I needed the income so I get all the shifts that I can even as a nolla contract, so I was coming back a lot more as compared to other. (5)”	It was a shock for me of not having any training at the beginning (5)	
“Actually I am still asking and I am still waiting for orientation. Yes, because in the in the workplace where I have been through yeah, everywhere before you start the on your first day you will be oriented, so of course they will have to tour you. This is the like this and this is the like that and then this is the	I am still asking and waiting for my orientation (11)	

activities and this is all the protocols we have here. But in there you have to orient yourself. (11)”				
“You mean osastonhoitaja? She's not there, but she waved. She waved like that. (16)”	Supervisor not included in the orientation (16)	Supervisor was not included in the orientation at work		
“Ei sillä lailla esihenkilö ei ollut mukana, mutta aina sai sitten mennä juttelemaan osastonhoitajahuoneeseen, jos on jotain. (6)”	Supervisor was not included in the orientation (6)			
“So I don't think that is sufficient. You won't even know where is the varasto and where are the stuffs. (8)”	Orientation is not sufficient (8)	Not satisfied with orientation at work		
“Like the part now if I work as the Home Care nurse.That's good. Not enough. But that's good. Yeah. Experience training is not enough. I was, but that's a good yeah. (18)”	Experience training at work was not enough (18)			
“Silloin ihan se TK:N kun tuossa osasto oli ensimmäinen työpaikka niin siellä ei ehkä ollut kovin riittävän pitkän, että tuntee, että yhtäkkiä on vähän niinku. (6)”	In my first work place I didn’t get enough orientation (6)			
“We cannot say that it is sufficient, but I think that it is enough for the time being that I am there so. (9)”	Daily exercise was not sufficient (9)			
“Be honest is I think it's not enough. Because some of them some of my colleagues think they didn't care all about you, some because some are accurate. Some are like that is, you know what I mean and not forcing them to teach me. It's up to them if they want, it's OK, but if they don't want, it's only OK for me. I just only work and do the best that I can to finish my work. That's all. (17)”	I think the orientation was not enough (17)			
“Yes, but yeah, everything is coming clear every day. Maybe now because of summer also most of the staff are on vacation now on the holidays, so not that much. Also not accurate orientation, but I'm depending on myself. Also I'm just opening the computer and I'm trying to search what is this? What is this? And always I'm asking also. What is this? What is this? How do you are doing that that like that? Why finish so, but yeah, opetushoitaja. She is very nice with me also. She's. Yeah. Everything is going good here. Thanks God. (20)”	Also orientation was not accurate (20)			
“My colleagues they are trying to speak to me in English, some of them who know how to speak	Colleagues trying to talk in English	Colleagues are speaking English	Positive interactions with	Colleagues and workplace

English. But I am forcing them to communicate with me in Finnish so that. I can be I can learn fast in a few time. (7)"	instead of Finnish (7)		colleagues	environment
"Maybe I'm just lucky also with my with my workmates and with my ohjaaja because she speaks really good English. (4)"	Personal perceptor speaking good English (4)			
"Yeah, like it's a good experience. I feel as far as now. Yeah, it's good like. As I already go to 1st placement in Home Care Services. Yeah. And from there, yeah. I have to go to really good experience and the staff is very cooperative and helpful because my language was not and is not good. Yeah. Until now so. They are helping me, like with speak the English and teaching me a Finnish as well, yeah. (18)"	My colleagues are speaking English to me (18)			
"And yes, and they're they were very helpful and they explained to us on like what is this for? Or do you have any questions? (14)"	My colleagues are very helpful (14)	Colleagues at work are helpful		
"Yeah, like it's a good experience. I feel as far as now. Yeah, it's good like. As I already go to 1st placement in Home Care Services. Yeah. And from there, yeah. I have to go to really good experience and the staff is very cooperative and helpful because my language was not and is not good. Yeah. Until now so. They are helping me, like with speak the English and teaching me a Finnish as well, yeah. (18)"	My colleagues are very cooperative (18)			
"But yeah, when I worked with the finish with locals and then I could they could like explain sometimes better to the residents and then they have they introduced us to them like and I think it's easier. (21)"	My Finnish colleagues are introducing me to my residents (21)"			
"And of course, most of the time both of us have are they are the one picking the right patients for me, yeah. (12)"	Colleagues are picking the right patients for me to work with (12)			
"Also good and bad experiences, as I can get help from people around, also people around me can improve their English. Although I think not just me, but many nurses, especially in the gerontology field would have bad feelings with people's talking, some time from the clients and sometimes the attitude of the colleagues. (22)"	I can get help from my colleagues (22)			
"And then they're really are trying hard to to, you know, to translate everything if we can. (14)"	My colleagues are trying hard to translate			

	everything to us (14)	
“My colleague also is helpful (1)”	Colleagues being helpful (1)	
“But yeah, when I worked with. With. The with the finish with locals and then I could they could like explain sometimes better to the residents and then they have they introduced us to them like and I think it's easier. (21)”	My Finnish colleagues are translating me to my residents (21)	
“And they're guiding us also. And if we don't, we will not know something we will ask them what's the meaning of this. What they want is like that. (1)”	Colleagues giving guidance (1)	
“And for some in our work, they have been like also supportive like. Because for me, I always ask questions like when I don't understand certain thing and if there's something that confuses me, I ask whoever is available and yeah, and then I think. Everyone has. Well, some has been able to like explain to me the process and the whole thing that's been going on (21)”	Some of my coworkers are supportive (21)	
“How do I call the actually nurses in ***. But they are the ones who are in charge of us. Like if we have problems, we have concerns in our workplace and then they coordinate everything to the supervisors in our office, in the facility or the kotihoito. But they are there they were the ones who gave us the orientation and visit us all that sometimes in the workplace to get to know how we are. Yeah, OK. In the workplace, how are we doing something like that? (15)”	Colleagues coordinate all our concerns to the supervisor (15)	
“It's they are helping me to develop my skills. In now they are teaching to give medicine and if I didn't know anything, he told me to just ask question if I'm if I didn't know something. (17)”	Colleagues are couraging me to ask if I don’t know something (17)	
“Yeah, like it's a good experience. I feel as far as now. Yeah, it's good like. As I I already go to 1st placement in Home Care Services. Yeah. And from there, yeah. I have to go to really good experience and the staff is very cooperative and helpful because my language was not and is not good. Yeah. Until now so. They are helping me, like with speak the English and teaching	My colleagues are teaching me Finnish (18)	Colleagues at work are helpful with finnish language

me a Finnish as well, yeah. (18)”			
“When we came here I think all my workmates are well informed that we are still learning the language, that's why they're all very lenient, teaching us as well, and also they are they are trying to use the much the much easier to words and language for us that would suit us, yeah. (2)”	Coworkers are teaching us (2)		
“We can understand each other and then so far my colleagues and my former, they are helping me to improve and develop. To throw the best of they can. (17)”	My colleagues are helping me to improve and develop my language skills (17)		
“Well, for I'm lucky that most of them are really helping me to understand the language. They're very helpful in terms of work, in terms of the language it's. (15)”	Codes\\most of my colleagues are helping me to understand the language (15)	Colleagues are helpful with Finnish language	
“It's easier for me to learn the language when I speak it all the time, so they try to communicate and help me with that and they're very nice and lovely. Good colleagues. (15)”	My colleagues are trying to communicate with me in Finnish (15)		
“When we came here I think all my workmates are well informed that we are still learning the language, that's why they're all very lenient, teaching us as well, and also they are they are trying to use the much the much easier to words and language for us that would suit us, yeah. (2)”	Coworkers are using easier words and language to us (2)		
“Yeah, like it's a good experience. I feel as far as now. Yeah, it's good like. As I already go to 1st placement in Home Care Services. Yeah. And from there, yeah. I have to go to really good experience and the staff is very cooperative and helpful because my language was not and is not good. Yeah. Until now so. They are helping me, like with speak the English and teaching me a Finnish as well, yeah. (18)”	My colleagues are very cooperative (18)		
“And especially in the countryside, which is my place now, came in, they really don't speak in English so. They really want us to speak in Finnish and also to understand them, but in our first few months. Like what I said, we are just like a statue waiting for them to approach us. And also we don't want that we just go beside them because. Maybe they will be they will be felt. I	Some of our colleagues don’t speak English (19)	Colleagues are not speaking English	Challenges with colleagues at work

know you have as a finish. I know I learned also the Finnish culture that you have this what do you call this this? The distance between the two. (19)”		
“Except that for others who don't speak English, I think they're shy to speak with me because. I may not be able to understand them. That's why sometimes we just stay quiet. We don't talk some, but most of them they try to talk to me, even if sometimes my answers are wrong because. (15)”	Some colleagues who don’t speak English are not speaking with me (15)	
“Also good and bad experiences, as I can get help from people around, also people around me can improve there English. Although I think not just me, but many nurses, especially in the gerontology field would have bad feelings with people’s talking, some time from the clients and sometimes the attitude of the colleagues. (22)”	I think my colleagues could improve their English (22)	
“Oh my colleague choose because they are. Yeah, they are finish. So I did sometimes they are not like for my, for example, if I am new and they will tell this OK, this is like this that I mean they will teach you, OK how to when I came here, it's just that I'm just observing because maybe because they can they can they won't speak English. (13)”	At first I just need to observe at work my colleagues cause they don’t speak English (13)	
“Yeah, I'm mostly like English speaking country where I will be very much appreciated for my own skills rather than intimidated in the workplace and even, you know, sometimes when I am with a coworker who does not speak, like not even a bit of English. And when I speak Finnish I lack of word. (8)”	Sometimes I’m working with people who doesn’t speak English at all (8)	
“During the orientation it's difficult to say because you're really your orientation we conducted it in a Finnish language and I barely understand much, I think it's only like the very basic and yeah, I struggle during that time because everyone just like, I mean, not everyone, but most just doesn't speak like or explain the whole thing to me in English. So yeah, it's like I mostly used observation and then if, like I could sneak, sometimes I use the Google Translate to so I could understand. (21)”	Most of the employees don’t explain things in English (21)	
“Yes so those observations that they	Colleagues	Colleagues are

get from me, they have reported to my supervisor and yeah, and my supervisor spoke to me and of course they are telling me what should I need to improve and what are the good things that I've done. The positive and negative thing. Yeah, that's not very good idea that I love here in my supervisor because she keeps on, you know, tracking me. OK, how was ***? How was how was doing? And yeah, so I we need to help him. So she is the one who is telling always that we need to understand that you know he's new here and been love. You can speak Finnish very well, so we'll need to guide him. We need to help him when it comes to communications with the patient. Yeah. (12)"	report to my supervisor about my orientation process (12)	reporting about me to our supervisor
"Maybe that's the that's the only thing I think. I think that's the only time that they have suggestions, but they are not telling to us, but they are telling it to our manager and then the manager will approach us that. That sometimes you will not do this and you will not do that. And sometimes if you what do you call this? So yeah, one comment that I remembered with them is that do not be in a hurry in doing the job. So do you want us to just break have a break. If our Finnish colleagues are also taking their breaks. So yeah, did I answer the question. (19)"	Our colleagues are not telling us if we did something wrong but they are telling to our manager (19)	
"And some sometimes our manager also have this meeting with us and that's the time that she also teaching us regarding the process in the nursing home and maybe some of our Finnish colleagues maybe reported the things that we need to learn and also the things that we did wrong and sometimes. (19)"	Some of our Finnish colleagues reported the things we did wrong to our manager (19)	
"To be honest, they are not teaching. you should do this you should do that you should not do this you should not do. They are not teaching that. So what we have to do is to just observe them so. And also we don't know if our if what we are doing is correct or not because no one is correcting us. Yeah. So I'm not sure if that is also part of your culture, if there is some somebody's doing a mistake, you will not, you will not. You will. You	Our collagues are not teaching us (19)	Colleagues are not helpful at work

will not confront that person, that they aren't doing the wrong thing. I'm not sure if that is your culture, but that's what I observed because. Oh, when we I think. Yeah. When we do something that is not. It is wrongfully done. We are just informed by our fellow Filipinos. That what we did is not a correct way so. (19)”			
“Oh well in our first day. We are just like statue in because maybe because we are this new and some of our colleagues are especially the Finnish colleagues. They are not still approaching us so when they need to do something. They are not asking us for, to, to go, then to observe and. They are not also teaching us to be honest, because maybe because of the language barrier because. Yeah. Here. (19)”	Some of our colleagues are not teaching us (19)		
“You have to observe for I mean you have to observe what they do. That's what I learned. I just observe because they're not always speaking to you or teaching you that the you will do this and this one, this one. No, I just learned by, oh, OK like that's I just yeah. That's the thing that I it's like. (13)”	I learned by observing my colleagues cause sometimes they don’t teach me (13)		
“To be honest, they are not teaching. you should do this you should do that you should not do this you should not do. They are not teaching that. So what we have to do is to just observe them so. And also we don't know if our if what we are doing is correct or not because no one is correcting us. Yeah. So I'm not sure if that is also part of your culture, if there is some somebody's doing a mistake, you will not, you will not. You will. You will not confront that person, that they aren't doing the wrong thing. I'm not sure if that is your culture, but that's what I observed because. Oh, when we I think. Yeah. When we do something that is not. It is wrongfully done. We are just informed by our fellow Filipinos. That what we did is not a correct way so. (19)”	None of our colleagues are correcting us (19)		
“Yeah, kipupumppu. That's it. But IV wise, I can give IV medicines, but the potassium not. (16)”	I have some limitations at work medication wise (16)	Limitations at work medication wise	Limitations at workplace
“Then I because I needed to be	Can’t give		

aside because I cannot give medications yet because of the licence. (3)”	medications yet because of not having the license (3)	
“Unlike in the Philippines, we have this licence to practise really the nursing profession. So in Philippines we are giving medications, oral IV medication and IV medications. We are allowed to give that medications. But here because we are assistant nurse here in the Finland, they are not allowing us to give medications even though it's an oral medications so. (19)”	Here because we are only assistant nurse they dont allow us to give any medications (19)	
“I am not allowed to give medication such as insulin, but you know for me it's quite it's very basic also and yeah also with the medications we're very limited. Yeah, and some some rules and stuff. Also very limited, but. Yeah, it's also, I mean, it's just I respect that because it's there. (2)”	Not allowed to give medications at work (2)	
“If I feel myself, I am capable of giving the medication, then I ask them to provide me a chance to give the law way. But I don't think with my limited finish I am capable of doing that even though I was a registered nurse in Nepal who was who I was working in ICU and Operation Theatre. So I know every medication, how it works. How do we do everything but. (8)”	I’m not capable of doing medication license because I can’t speak Finnish (8)	
“And before passing the medication exam, I didn't, you know, I didn't handle medicines and I didn't handle like communications with family members and stuff like that. So it's really more just day-to-day bedside care of the resident or the patient. But now that I you know I'm moving up to a slightly higher role of sairaanhoitaja. So I have been trained in all the medications and how to give the medications and office work like reading the. (5)”	Limitations at work first when working as practical nurse (5)	Limitations at work as nurse
“So actually and also the approach in the Philippines is very different from the approach here, especially that protocols, it is very difficult a different I should say so in here your skills it is controlled. So you cannot freely do your skills. Our job is only limited because there are scope of limitations that are that we have to follow. So I think I'm not	In Finland your skills as a nurse are controlled (11)	

that satisfied to give my full care which I am used to do. (11)”			
“No, they're actually enough because they, my manager, always and we always had this meeting like this small talk with the supervisor. So like, how are you? How are the things going? How is your language proficiency? So he's very supportive. (14)”	Our manager is very supportive (14)	Supervisor at work is supportive	Receiving support in educational and work settings
“And then there's also after you do the courses, you schedule like a one-on-one like a coffee time with your supervisor. So like if you have any questions or if you have any suggestions like you want to change or you feel like it's not useful or whatever. So you can have that one-on-one time with a supervisor. (5)”	Scheduled one on one coffee time with supervisor (5)		
“My boss is very supportive so far in my programme now. (4)”	Boss being supportive for language studying (4)		
“It's like there were there were we had a meeting and then they introduced that she will be your ohjaaja. Yeah. And then our manager told us that we should speak. I mean, if ever, I mean, if they will speak to us, it should be English and then finish or finish English so that we understand. (14)”	Our supervisor informed our workplace that our colleagues need to speak English to us (14)		
“No, still actually it was supposed to be last week last week but pomo she was in, she was in vacation, but she will come for she will come tomorrow. She will start and maybe I am elaborate tomorrow. So maybe I will meet her tomorrow or it will be this week. Just will be orientation. How are you now? How do you feel now? Because she asked me in the beginning. I told her I don't know. I feel very strange here. Nobody's talking with me. Nobody is supporting me. But I think it will be this week and I hope I hope everything will be OK. (20)”	My supervisor asked me in the beginning how am I and how do I feel (20)		
“Like days by day. OK, now I learned that it's this is the one I should do but my boss said if you want to ask something you can ask because they the good thing for them is when you ask, they will really tell you. But it's just that it's not every day that I'm not always like for me, I'm not always asking because I feel that my I get. I don't know how to ask it there in in	My boss said if I want to ask I can ask (13)		

properly in pinch. So sometimes I just do search, so that's what I learned, yeah. (13)”		
“They are she is supporting me and she's advising also. Yeah. And everybody they are telling in that, you know, support ***. He's one year and half only in Finland. I have also long experience in this field but. So it will take time and they are considering this point. I feel they are considering this one and I always are telling me take your time in learning the language and everything is being going good. (20)”	My supervisor is supporting me (20)	
“It's like there were there were we had a meeting and then they introduced that she will be your. Yeah. And then our manager told us that we should speak. I mean, if ever, I mean, if they will speak to us, it should be english and then finish or finish English so that we understand. (14)”	Our supervisor had a meeting with us when we arrived (14)	
“They are telling me what should I need to improve and what are the good things that I've done. The positive and negative thing. Yeah, that's not very good idea that I love here in my supervisor because she keeps on, you know, tracking me. OK, how was *** How was how was doing? And yeah, so I we need to help him. So she is the one who is telling always that we need to understand that you know he's new here and been. You can speak Finnish very well, so we'll need to guide him. We need to help him when it comes to communications with the patient. Yeah. (12)”	Supervisor is telling my colleagues that they need to support me to learn (12)	
“And in cooperative. Language environment and then, but my boss is like very, very, very nice. But the coworkers are like. (8)”	My boss is very nice (8)	
“And our manager is good also, they're always asking us if we were OK, how's work? Is there any like hardship or good? (1)”	Supervisor making sure if everything is ok (1)	
“My supervisor also at work and he's very supportive actually. I have to no, there's very good, very, very good. There's some people you know, supporting me and understand. (10)”	My supervisor is very supportive (10)	
“Yes so those observations that they get from me, they have reported to my supervisor and yeah, and my supervisor spoke to me and of	Colleagues are giving me feedback about my	Receiving feedback at work

course they are telling me what should I need to improve and what are the good things that I've done. The positive and negative thing. Yeah, that's not very good idea that I love here in my supervisor because she keeps on, you know, tracking me. OK, how was ***? How was how was doing? And yeah, so I we need to help him. So she is the one who is telling always that we need to understand that you know he's new here and been love. You can speak Finnish very well, so we'll need to guide him. We need to help him when it comes to communications with the patient. Yeah. (12)"	work (12)	
"Because we have actually in every, I think that maybe three months we have this. It's like a evaluation sheet and then we answer it if we have this problem. So I think my employer. (13)"	Every three months we have evaluation at work (13)	
"Something's like very much nice and they are like supporting their students, even the tutors teachers and the degree heads everyone is like our students cannot suffer like this. So they are, like, always supporting us. (8)"	Teachers are supporting us (8)	Receiving support from school
"And then, but our school, our teachers, our like faculty members, whoever we're connected with the what do you say agency. So then they always like backs up and they supported us like they used to. (8)"	Our school was supporting us (8)	
"They're having one-on-one interview, like how? How is it going in your workplace? Do you have any problem with that every four weeks during our first months last year and then? (3)"	Head of education having one on one interviews regularly (3)	Receiving support from the head at work
"Hmm. No, but they just telling me everything that you understand that language is very difficult, but I am pretty sure that you can handle this. Who knows? Yeah, they told me that. Who knows, as time goes by that you can speak very well, you can communicate very well with the patients. And then. Yeah, we don't know what will gonna happen next. (12)"	Employer is supportive about learning the language (12)	
"The path from the *** government which handles us they came here and they were like hey, are you like do you like to stay here? Like are you like do you think you would stay here for long? Do you like the	Employer is asking are we getting used to the place (2)	

place? Are you getting used to it? (2)”				
“And then it's your employer that will help you umm with taking that medical exam so. (5)”	Employer helping with the medical exam (5)			
“Visit us all that sometimes in the workplace to get to know how we are. Yeah, OK. In the workplace, how are we doing something like that. (15)”	Employer is making sure of how we are doing at work (15)			
“The ylihoitaja. Some things she was the one who hired me, you know? So yeah, she was there checking us out and then that she left. (16)”	The head nurse was checking us out after we started (16)			
“They are very supportive to us and with regards. (14)”	My colleagues are very supportive (14)	Colleagues at work are supportive		
“They're very nice and lovely. Good colleagues. (15)”	I have good colleagues (15)			
“Yeah, but also it's very difficult to have friends or something to practise that language even. So deep. But they are supportive. Most of them I can say 70% are very good supportive but you have to ask if you want to help. If you want to. If you want to understand something, if you want to know anything. They will not come to you until your new will teach you. You have to ask. (10)”	Most of the colleagues are supportive (10)			
“They try to cheer us if everything we feel if we feel bad sometimes like this and like that. (1)”	Colleagues being cheerful (1)			
“I don't understand much of what they say, but they try to because they say they told me that you have to speak all the time the language so you'll be able to learn them. (15)”	My colleagues are encouraging me to speak Finnish with them so I will be able to learn (15)			
“Well, yeah I think when it comes to like caring for our residents, because I was in an elderly care home, yeah. How we assess the clients like because, yeah, before like it's been like very assessment is our basic like a skill that's practised from every day and yeah. We have the I have been able to use that like when our residents like doesn't look well during the day and then or. Also, when we have residents who have wounds or special ulcers due to like lying in the bed for a long	My previous skills as a nurse has been take into account in the basic care of our residents (21)	Previous skills have been taken into account at work	Recognition of prior nursing experience	Recognition and validation of prior nursing competence

time. (21)”		
“Yes, actually yes, 100% since I have felt that they trusted me with what I'm doing and from the day that I started, like a day, a day after or a day few days after they have left me alone, since they have also explained that you already had the skills. You just need to practise the language. So by letting you go you just need to to tell us what to do or what you need to do so yeah. The the trust was there, so I think they really believe that my skills are. (7)”	Previous skills has been taken into account in current task (7)	
“It's for them I think the education is like for the formality that I have, I go because I needed to perform my duties but for them when they hear that I'm that I'm a nurse, the patients or the nurses hear that I was a nurse in the Philippines. They don't hesitate to have them being cared for by me. (2)”	When my colleagues hear that I’m a nurse in the Philippines they don’t hesitate to ask me to do more things at work (2)	
“Just the basic nursing care that I do, even as a lawyer. Yeah, that is taking account. (8)”	They have taken account my basic nursing skills what I have (8)	
“I think it's more on the language because we don't actually have a lot of training because for the skills I think. I don't. It's not being proud but I did not need to train for basic nursing care procedures. Yeah, because I already know it. It's more on the language that they focus. Yeah. (3)”	I don’t have to train for the basic nursing procedures (3)	Already having the nursing skills for the work
“I have felt that they trusted me with what I'm doing. And from the day that I started, like a day, a day after or a day few days after they have left me alone, since they have also explained that you already had the skills. You just need to practise the language. So by letting you go you just need to tell us what to do or what you need to do so yeah. The trust was there, so I think they really believe that my skills are. (7)”	I have already the skills for the work (7)	
“Yeah, I'm working now, so I'm in this one cardiac, cardiac and pneumonia ward. And they consider this. I have 12 years’ experience in Kuwait City, so they consider this year of experience in my work and in my salary also. They consider	They consider my previous work experience in my salary (20)	Employer considering previous work experience in salary

this one yes. (20)”		
“And in our employer right now, we were given work experience bonus because the aside from the basic salary, we were given additional because we had experience. (3)”	Getting work experience bonus due to previous experience (3)	
“Before I was in special area, but this is OK as long as I stepped in the in the hospital, I was really happy already because I saw a ward I'm in good to get a, but it's internal medicine so it's almost the same as what I'm doing in, you know, in the Philippines like we admit people with discharge people we take care of people. (16)”	I’m working in the same speciality where I have my work experience in the Philippines (16)	Employment contract matching with background
“Contracts align with candidates' education and qualifications, boosting job satisfaction, and include reasonable restrictions like non-compete clauses. (22)”	Contracts align with education and qualifications (22)	
“Yes, thank you. So, yeah, I'm so yeah now. So it worked well and yes, to answer the question I my work is in line with my studies. (5)”	Now my work is in line with my studies (5)	
“Yes. Now it's OK because already we are staff nurses there. I am staff nurse in Egypt already. So now in Finland also sairaanhoitaja. Yeah. Now contract is OK now. But before this contract the previous contract it was lähihoitaja. I only because of language but now. By blessing of God, language is little bit OK, so now I'm working at sairaanhoitaja now yes. (20)	My current contract is as registered nurse (20)	
We're working as a sairaanhoitaja, yeah. (3)”	Working as a nurse with guidance (3)	
“Keskussairaalan kuntoutusosastolla sairaanhoitajana. (6)”	I work currently as a nurse in hospital (6)	
“Yeah, I'm working sairaanhoitaja in ***. (3)”	Working as a nurse in Finland (3)”	
“Finished on 31 May 2023. This may last May. Then yeah, then I working now, but my language is not that good. I can manage at work, but not professional of course as. (10)”	I’m working as nurse in Finland (10)	
“Yeah, that's a good questions and that my previous educations, it's helped me a lot to use in there. Yeah, actually I'm I want to study like the counselling nurse or some, you know, psychiatric view of nurse. Yeah, but the main things. Is	My previous education helps me a lot in my current job (18)	

maybe I will continue this dream in a futures as a master or but now yeah my previous educations and working experience help me a lot in my nowadays job and the practise placement also. Yeah, that's. (18)"			
"Yeah, I think so, because it is in the healthcare, but not as a nurse because you know that we need to study here. Yeah. And then after that. (9)"	Employment contract matching with education (9)		
"Yes, the employment contract with the where are you work? Yeah, yeah, it match. (21)"	Employment contract matches with my education (21)		
"Because I don't know because I was. Yeah. Even I'm a registered nurse there I was giving medication like that. But yeah, when I came, when I come here, I feel like I'm yeah, I'm assistant. But it's like a caregiver. All that. Yeah. Doing really a basic care, I mean but even I miss that. But we are now we are on process for a student, but maybe when we graduate I hope I can change my. I want to, you know, like sairaanhoitaja. I mean like that. So I have a lot of to take this before I will be sairaanhoitaja. (13)"	I think my employment contract doesn't match with my education (13)	Employment contract is not matching with background	Lack of recognition for previous nursing experience
"It's not at all it's not my area of experience. You know, you have to. It's different setting. Like I'm not in the right position, but I cannot say I like whatever I'm doing because otherwise I will be, you know, at home. (10)"	I feel I'm not in the right position (10)		
"Yeah, yeah, I am now working on a *** sairaala as practical nurse. (7)"	Now working as a practical nurse (7)		
"Right now I am a lähihoitaja. (8)"	I'm working now as a practical nurse (8)		
"Right now I'm working in laakson sairaalas as sairaanhoitajaopiskelija (16)"	Right now I'm working as a nursing student (16)		
"Yeah. I think it's not even practical nurse. It's the assistant level of the. (2)"	Working as assistant nurse (2)		
"Well, I've never worked as a nurse assistant in the Philippines or for me, this is really a big change to me and it and of course, I really have to learn it because I was a community health nurse and a vaccinator in the Philippines. And although I have, I know how to do it, I can take care	Working as assistant nurse is not my experience (15)		

of the elderly and but my experience is not. (15)	
"Same. To be honest, the employment contract that we've signed here is for only a caregiver, for hoiva-avustaja style like that which is, which is I'm overqualified on that because I have so many licences in different countries. I'm a licenced nurse, Saudi Arabia, Singapore and Philippines. And then I came here and became caregiver just like that. But so far I'm studying now to become a practical nurse here. (17)"	I'm overqualified for my employment task (17)
"So far I'm very honest. I feel downgrade from what I'm doing now because from a professional nurse in other countries I'm working in operating room in emergency room. And then here I'm just a caregiver. (17)"	I feel downgraded for my task because I'm a professional nurse with work experience (17)
"In my opinion, I don't think that it matches because I did had, yes, a bachelor's degree in the third world country, but it's still the bachelors in in what I think but since I'm moving to a first world country, I guess I do understand that they have different standards here in I guess I have to like study a little bit I guess yeah, I think it's reasonable in some ways for me in my opinion. (16)"	Employment contract not matching with education (16)
"OK, so what are you feeling right now is that yeah, from a registered nurse in the Philippines to an assistant nurse here. So I feel like that I degraded my course. So from registered nurse to become an assistant nurse. So as assistant nurse here, we have limitations. (19)"	I have degraded my course because of working as assistant nurse even I'm registered nurse (19)
"Umm I think no, it's not because the work here is on a vocation level. And we graduated in a bachelor's degree level in the Philippines, there is no really practical nursing, so I can say that the level of it, it's like caregivers, I don't know if. (4)"	Employment contract not matching with education (4)
"It's not at all. It's it's, it's not my area of experience. You know, you have to. It's different setting. Like I'm not in the right position, but I cannot say I like whatever I'm doing because otherwise I will be, you know, at home. (10)"	Employment contract is not matching with my background (10)

"Sometimes you know you miss your skills, what you have done before like. IV inserting the NGP inserting or the catheterization, everything like that. Everything you do in ICU, I don't do here now. Yeah sometimes. (4)"	Feeling of missing previous skills (4)	Not being able to use previous skills at work	
"No, actually because it was working as an emergency nurse for how many years? And then here it's like only I'm assisting more on assisting the patient. It maybe if I'm gonna work in the hospital, yes, I can use my previous skills. But as of now, no. (1)"	Not being able to use previous skills at work (1)		
"Previous experience in the Philippines as a nurse, I had an experience also in the hospital but just what I've said. Because the kind of job I have right now is a job for the nurse assistant in the Philippines and we were not able to experience it really at first hand because we already are licenced nurses so. Those honestly, it's not my I don't have the skills as an assistant nurse, but I know how to prepare beddings. I know that it's really different when you have experience before than. So we learned that, but fortunately we're able to learn how it goes. Like changing the diapers, the best way and the techniques and so you don't hurt your back. Something like that. (15)"	My job is now different than what I have skills for (15)		
"Yeah, I'm just portraying assistant nurse, so skills in terms of skills, I think I'm not honing my nursing skills as I'm doing way back in the Philippines so. Skills like inserting IV medications, inserting IV. Yeah, having an IV line, inserting IV line before and also assessing the patient, somehow assessing the patient, I'm not practising it here because when patient have complaints, we need to ask assistant for our sairaanhoitaja or the nurses. So, yeah, somehow we in yeah, somehow I'm. My skills is I'm. I'm not improving (19)"	I'm not honing my nursing skills because I'm working as assistant nurse (19)		
"Yeah, yeah. Now Valvira licence. Since you're on well, I think. (10)"	I'm licensed as a nurse in finland (10)	Being able to get a registered nurse license in Finland	Recognition of previous nursing degree
"Joo. (6)"	Being licensed as a nurse in Finland (6)		
"OK. Yeah. Oh OK, so I am. I am a licenced. (12)"	I'm licensed as a nurse in		

	Finland (12)	
"So and then you have to pass that exam with a 75% passing rate before you can get your Philippine licence, and here in Finland we just had to pass our university course and then after that send an application to Valvira online and then pay the fee Valvira and then that's it. And then you know, you can already work as a registered nurse. So it's relatively easy and very quick, like less than a month and then depends on where you are working you. (5)"	Licensing process being fast (5)	Nurse licensing process was fast
"I think it's very. Very quick because with after graduation, we just the day of graduation, we already submitted our degree certificate and then we waited for it. While there's the decision and. (3)"	Licensing process was quick (3)	
"Very easy, just I graduated in December. Last December I applied in maybe in the middle of December this graduation this day. So I think by the end of December I have got my Valvira. I have. Everything was ready. Yeah. (20)"	Getting the nursing license easy and fast after graduation (20)	
"Quite clearly to make and fast. (22)"	Licensing process was clear and fast (22)	
"Actually, when we applied in Valvira. Oh, it's just a short period of time that they send us the to this it's just been a short period of time, yeah. (12)"	The licensing process was only short period of time (12)	
Joo. En muista ollenkaan, mutta ei ihan. Ei varmaan mitenkään kovin pitkään. (6)	Licensing process didn't take a long time (6)	
"Yeah, yeah, yeah. One year is like less due to we already there's some code in home country. So yeah, that's a good, yeah. (18)"	I have to study one year less in finland because i studied in my home country (18)	Having to study less amount of time due to nursing degree from home
"1 1/2 year I think because we started this June and it will end next year December. (2)"	Needing to study 1,5 years of nursing (2)	
"Oh, very, very, very big because it was a top up nursing course. First in our education we didn't. We did, we did not need to study for 3.5 years only 1 1/2 years because we already had education. (3)"	Having to study only for 1,5 years (3)	
"Oh, very, very, very big because it was a top up nursing course. First in	Previous degree taken	

in our education we didn't we did not need to study for 3.5 years. Only 1 1/2 years because we already had education. (3)”	into account in studies (3)	
“Yes, yes, it's like that. And we started the classes around February and we and it last year on December, December 15th at our graduation, yes. (1)”	Studying 10 months for practical nurse (1)	
“Due to the safety issues there, I didn't prefer to go to US and I searched in Google what is the best country in the world. And I find that Finland is happiest country and everything is good. Then I like to travel and visit new cultures and new places so. I searched how to come here then I found company that like coordinate this top up programme for nurses. That we have already bachelor degree in other countries. Then I applied to ***. Then accepted. Then I started for one year and a half. (10)”	Having to study nursing for one and half year (10)	
“If you have your registered nurse licence from the Philippines, the study course is shortened to become sairaanhoitaja. So this was appealing to me because of my age. (5)”	Having to study only short period of time due to having nursing degree at home (5)	
“Tai sain hyväksi luettua jonkun verran, mutta mä vaan opiskelin niinku en normaalisti. (6)”	They recognised some studies from my degree in Japan (6)	
“Maybe less than a month. (9)”	Licensing process took less than one month (9)	Nurse licensing process took less than one month
“It took me about only two weeks maybe. Yeah. (11)”	It took me about two weeks to get the license (11)	
“It takes almost two weeks after i graduated from the top of nursing. (10)”	I got the nursing license after two weeks of my graduation (10)	
“I think it's very very quick because with after graduation, we just the day of graduation, we already submitted our degree certificate and then we waited for it. While there's the decision and maybe like 2 weeks. (3)”	Getting a nursing license in two weeks (3)	
“As a practical nurse, yeah. It isn't	Licensing	

that long. Two weeks for the Valvira processing for processing the Valvira. Yeah, I think not even like 2 weeks. (21)”	process for practical nurse was two weeks (21)		
“Yeah, not yet. I'm still I'm a practical nurse yeah. (13)”	I don’t have my nursing license yet (13)	Not being able to get a registered nursing license in Finland	Non-recognition of previous nursing degree
“No, no, not yet. I am here like the student. But in the Philippines, I am a registered nurse. (14)”	I’m not licensed as a nurse in Finland because I’m still a student (14)		
“Not yet, because we have to undergo studies. (2)”	Not licensed as a registered nurse in Finland due to need to study more (2)		
“No. (8)”	Not yet licensed as a nurse in Finland (8)		
“Yeah. In the Valvira, yeah. (3)”	Being licensed as a nurse in Finland (3)		
“Not yet, but I'm in I'm doing the nursing programme here in the. (4)”	Not yet licensed as a nurse in Finland (4)		
“Not yet, but we are studying in as we're studying nursing here because our two diplomas are not accredited here. Degrees are not accredited here (15)”	Not licensed as a nurse cause our diplomas are not accredited here (15)		
“As a nurse? Not yet. Yeah. Maybe. Practical nurse. Yeah, I just had my Valvira last month. Yeah. (21)”	I have been licensed as a practical nurse in Finland (21)		
“Yes. Lastly, what I am. (1)”	Being licensed as a practical nurse in Finland (1)		
“As a practical nurse only. (22)”	Licensed as practical nurse in Finland (22)		
“Yeah, I think so, because it is in the healthcare, but not as a nurse because you know that we need to study here. Yeah. And then after that. (9)”	Needing to study more to be a nurse (9)	Having to study nursing degree in Finland from the beginning	
“No mä aloitin ihan alusta, että kun ei ollut ainakaan tai ei ole ennen ollut vastaavaa *** ainakaan, että olisi japanissa valmistunut sairaanhoitajaksi ja suomeen. Niin mä vaan aloitin ihan alusta että mä	I started from the beginning by applying to school to study nursing (6)		

hain kouluun ja sitten opiskelin. (6)”				
“For that, as lähihoitaja Yeah, at that time, which is in line with my studies, because I couldn't get sairaanhoitaja yet because at that time I was still a student. (5)”	I worked first as practical nurse cause I was still studying nursing (5)			
“Except the healthcare field, I don't have quite any experience in any other field to be honest, and I enjoy this field so much. But in Nepal I was a sairaanhoitaja, I had a registered nurse, but here my degree doesn't comply with that. So I have to start from the very first and as soon as I completed my enough credit, I was not getting a job. (8)”	I came to Finland to study nursing (8)			
“Yeah. First, I need to study all of the bachelor's degree here in Finland before I can apply it for a job. So and after that. (12)”	First I need to study bachelor’s degree in Finland before I can work here (12)			
“From my patients, it's really good. I've never experienced any discrimination because like I'm a I'm a foreigner, so the patient and the residents have all been so kind and so patient. (5)”	All patients are kind (5)	Good experience with patients at work	Positive experiences as an international nurse in Finland	Overall experience as an international nurse in Finland
“That's the reason why I stayed in this job in this specific job for long and that I applied for the permanent position because, like I really connected with the residents now, and because I've also experienced working part time in the hospital. (5)”	I have been able to connect with patients (5)			
“Well, I don't have really adult patients right now. I have this grandpa's and grandmas at work and they're easy to work with sometimes. Of course they get cranky and angry, but the most the most is they're not complaining adults. That's the good thing about elderly people. But yeah, sometimes you argue with them, but. (16)”	It’s easy to work with patients (16)			
“Well, they are so patient in teaching me every time I ask something every time I show my interest in helping them because I want to learn. They actually accept me and even explain me more. Well, I think that it depends upon you as an international student, how you how you. (9)”	My patients accept me as international nurse (9)			

"And also I'm lucky and I'm very thankful that some of my patients, they're the ones who teach me to speak Finnish and they say because I've I told them that I know I'm studying. I'm studying Finnish language and they say that they will teach me so as of as I, so if I don't know what it's it is in suomeksi they will tell. And I learned from them that pusero and paita is the same. (15)"	Some of patients teach me to speak Finnish (15)	
"Patients there are patients that can speak English as well. It it's actually easier for us. And then yeah, they know that we are from the Philippines. And then if we can if like if we do have this, if we mispronounce the word the word then they correct us. (14)"	I have patients who can speak also English (14)	
"Yeah, that's good they are really nice. Except for the one. But most of them, they're really nice that they, they know that you are you cannot too much speak in there. So they're very nice actually. I'm lucky because I don't know if the others like but for my workplace it's good that they are really nice too. Even my patients here. Yeah, I absolutely loved it and they love you too. That like we are, they're understanding that sometimes we didn't know this and then they will. It's not they're not like from my work with that there so you should be inactive but here no you should just take slowly like that so. (13)"	My patients are understanding (13)	
"And then the more this then the more that I have been exposed to them through time, they have been more acceptable of me. Yeah, and yeah, until I think for me it just takes some time. For them to adjust and also for me to adjust. But yeah, yeah. (21)"	The more I have been exposed to my resident the more they have accepted me (21)	
"It's for them I think the education is like for the formality that I have, I go. Because I needed to perform my duties but for them when they hear that I'm that I'm a nurse, the patients or the residents hear that I was a nurse in the Philippines. They don't hesitate to have them being cared for by me. (2)"	When patients hear that I was a nurse in the Philippines they don't mind having me taking care of them (2)	
"Mutta aika ei suurin osa on hyvin ottanut vastaan. (6)"	Most of the colleagues have received me well (6)	Good experience with colleagues at work
"So that's a really positive thing that	Some	

I experienced for the employee or coworker side. I guess it you can say it's 5050. There are a lot of coworkers who are very helpful and are very patient with the language side and they, you know, assist and try to make the workplace like. Like equal and they don't discriminate. (5)”	coworkers try to assist us at work (5)
“So far there is no like racism (4)”	So far there is no racism (4)
“So that's a really positive thing that I experienced for the employee or coworker side. I guess it you can say it's 5050. There are a lot of coworkers who are very helpful and are very patient with the language side and they, you know, assist and try to make the workplace like. Like equal and they don't discriminate. (5)”	Some of the coworkers are helpful (5)
“Yes, but yeah, everything is coming clear every day. Maybe now because of summer also most of the staff are on vacation now on the holidays, so not that much. Also not accurate orientation, but I'm depending on myself. Also I'm just opening the computer and I'm trying to search what is this? What is this? And always I'm asking also. What is this? What is this? How do you are doing that that like that? Why finish so, but yeah, opetushoitaja. She is very nice with me also. She's. Yeah. Everything is going good here. Thanks God. (20)”	Our head of education is nice to me (20)
“Yeah. So it, for example, the division of work instead of other ratio is one is to six. I will be given only like 3. So the ratio will be one is to 7 and I'm I had three so. (3)”	Having less patients at work as an international nurse than others (3)
“Muuten sitä ei mä kuvittelin, että olisi enemmän sellaisia ihmisiä jotka ei halua ulkomaalaista taustaa ulkomaalaistausta hoitaja, mutta yllättävän vähän niitä oli, mutta sitten. (6)”	There is surprisingly less of people who didn’t want to work with foreigners (6)
“Ohh OK. They're they're good. Yeah, they are kind to us. (14)”	My colleagues are good to me (14)
“Ohh OK. Actually, because this one. I don't want to be negative. But uh, you know, some people are very nice there. There's racist people also. You know, there is percentage. Yeah, I have to be clear with this one that there's people they don't	Some people are nice to me (10)

like. (10)”		
“And then we yeah, especially for a lot when it's like a place where it's a lot of international nurses and we help each other because some have been here longer than others. (5)”	Helping each other with language in international team (5)	
“So that's a really positive thing that I experienced for the employee or coworker side. I guess it you can say it's 5050. There are a lot of coworkers who are very helpful and are very patient with the language side and they, you know, assist and try to make the workplace like. Like equal and they don't discriminate. (5)”	Some coworkers don’t discriminate (5)	
“Watch or supervise you I think they do respect my experience as a nurse and there for them it's just the formality that I had to undergo the education. (2)”	Colleagues respect my experience as a nurse (2)	Feeling respected at work
“I have never being Asian I mean being Asian, I have never been respected like this and I have never been looked up equal. (2)”	In Finland I feel respected as Asian (2)	
“This is quite sad at first, but I'm getting used to it because I have never been to a place where I have experienced the kind of respect and also. (2)”	I have never been a place where I received this kind of respect (2)	
“I think slowly they are they are giving me more and more responsibility. So like I can see because previously as the as in lähihoitaja position. (5)”	Getting more responsability at work (5)	Getting responsibility at work
“And from the day that I started, like a day, a day after or a day few days after they have left me alone, since they have also explained that you you already had the skills. (7)”	Being able to work independently (7)	
“Lääkärinkierto and you know I'm transcribing. Yeah, the communications of the doctors to, you know, orders in the pharmacy. And if there is. (5)”	Communicatin g with doctors at work (5)	
“Lääkärinkierto and you know I'm transcribing. Yeah, the communications of the doctors to, you know, orders in the pharmacy. And if there is. (5)”	Doing orders to the pharmacy at work (5)	
“Allowed and uncomfortable to talk to these family members and give them updates about their about their about the patient or the resident. So I feel like, yeah, I'm leaning more into I'm slowly learning the role of sairaanhoitaja, which is good, but still the Finnish language is still the most challenging part everything.	Being allowed to communicate with patients family members (5)	

(5)"		
"And yeah, and I have applied like my a lot of knowledge like I already been working in a hospital as emergency department in my home country. Yeah, and I thought much more knowledge. Yeah. And I have been using that little knowledge also in a Home Care Services like I have done dressing, lood sugar test, blood pressure measurement. Like independently. And they are. Wow. You can do it. Yeah. You have lot of knowledge and practise. So yeah. They appreciate my. (18)"	I have been able to apply my knowledge at work independently (18)	
"And before passing the medication exam, I didn't, you know, I didn't handle medicines and I didn't handle like communications with family members and stuff like that. So it's really more just day-to-day bedside care of the resident or the patient? But now that I you know I'm moving up to a slightly higher role of sairaanhoitaja. So I have been trained in all the medications and how to give the medications and office work like reading the. (5)"	Getting more responsability at work as a nurse (5)	
"Oh no, they don't have restriction. I think they have restriction when we sign the contract but we don't have this. (13)"	No restrictions in the contract (13)	Recruitment contract didn't have any restrictions
"Restrictions. I don't think so. (9)"	No restrictions in the contract (9)	
"It was not restricting us. They were not restricting us from any ways, but it's like you know, like when they when do you have a deadline for the payments and when we ask them to do the thing about extending the date because we are students and only working for 30 hours, we even don't get that 30 hours to work because we are nurses. (8)"	No restrictions from the agency (8)	
"I think there was no restriction about thing I guess. But I mean we need to stay in ***. For three years. (14)"	I think there is no restrictions in the contract (14)	
"I don't remember, but I don't think there was in in my memory like they. (5)"	No restrictions from consultancy (5)	
"Loaded in the no specific but of course our contract is under ***, so for that we are contract is under *** is 2 years so we can't of course we	My contract doesn't include any restrictions I	

can apply then in other come in other employee employers so. That's it. We know that we can apply it to other companies or employers then other than I know. (15)"	can apply somewhere else (15)		
"No, no. Nothing. Yeah. Actually we're we're a bit disappointed because after the information that we got, then we have to do everything on our own. So, so. (3)"	No restrictions from consultancy (3)		
"Umm. I know. No, I think if you umm would not want to pursue you can end it immediately like that. (4)"	No restrictions in the contract (4)		
"When I when I was lähihoitajaopiskelija. Yeah, I was tied up in a contract for good for two years or three years, I think, but when I graduated and we had a new boss in the work site, it was replaced, but that it was replaced. That you can leave whenever you want. You can do keikkari as long as. (16)"	After graduating as practical nurse I was able to relocate job wise (16)	Being able to relocate	
"I mean two years contract, so we can just still want to continue to relocate. As you know, I think. (2)"	I'm able to relocate after two years (2)		
"Yes, it says there that within the period of while I was like in the. What was the while I was in that? Practise like probationary period. We cannot like go or work from for other companies or other we cannot take any like part time jobs. But after that when because we already finished the practical nursing course? Yeah, we have been like allowed to take part time jobs from other to companies. Yeah. (21)"	After finishing practical nurse school we are allowed to take part time jobs from other companies (21)		
"Can Transfer two different national facilities here to *** it depends on the man power needed to your workplace. You can request into your pomo if you can transfer just like now I'm asking my permit to transfer me into the city. (17)"	You can request a transfer from your supervisor (17)		
"Ask you're a nurse, right and when I said yes, I'm a nurse. Yeah. And can you, like, do clean this one. Yeah. There's no detail. I do it all the time in the Philippines. And she's like, yeah, because, you know, I don't do it I don't like doing it. She said can you do it also do you do injections? Yeah, I do it a lot. Yeah. You can do it. I can just. (2)"	Colleagues let me do nursing things when they hear I'm a nurse in Philippines (2)	Feeling trusted at work	
"The trust was there, so I think they, they, they they really believe that my skills are. (7)"	Colleagues trust regarding my skills (7)		

"I have felt that they trusted me with what I'm doing. And from the day that I started, like a day, a day after or a day few days after they have left me alone, since they have also explained that you already had the skills. You just need to practise the language. So by letting you go you just need to tell us what to do or what you need to do so yeah. The trust was there, so I think they really believe that my skills are. (7)"	Feeling trusted of what I'm doing at work (7)		
"Also good and bad experiences, as I can get help from people around, also people around me can improve there English. Although I think not just me, but many nurses, especially in the gerontology field would have bad feelings with people's talking, some time from the clients and sometimes the attitude of the colleagues. (22)"	Bad feeling about clients talking about you (22)	Negative experience as an international nurse at work due to attitude of clients	Challenges as an international nurse in Finland
"So yeah, and then at the beginning I think, yeah, we have like residents who doesn't like foreigners because we don't like they said that I don't understand what she is asking. (21)"	In the beginning our residents didn't like foreigners (21)		
"Vaikka jos mä oon käytävällä ja olisi ja sitten tulee joku vaikka potilas tai muualta osastoilta hoitaja tai joku omainen, niin ensimmäisenä yleensä ihmiset sitten hakeutuu suomalaisiin hoitajiin, että ei katso edes mua silmiin, niin se on ehkä sellainen. (6)"	Sometimes patients and nurses will talk first with Finnish looking nurses (6)		
"So yeah, it's a mix of positive and negatives, yeah. Actually the colleagues, they know that I'm a foreigner. The patients, some thought I have already lived there for long since the way I speak to them they thought that I already know how to speak. Some say that oh, you just arrived here and you speak you went finnish already like that. I'm just saying that I only know basic words and I'm still learning and in terms of my colleagues. They are trying to speak to me in English, some of them who know how to speak English but I am forcing them to communicate with me in Finnish so that. (7)"	Experiences as an international nurse regards to patients is a mix of positive and negative (7)		
"Disstress when they are in hospital, so they're not, they're not always considering your situation, that you cannot speak. Sometimes	Patients are angry due to not speaking the language		

they're angry. (3)”	(3)	
“And umm, in the hospital they are feeling more sick, I guess because you know, the reason that they go to the hospital is that they are really feeling something bad or so about themselves and they're not healthy. So I guess they have, they don't really like to talk a lot and they don't, you know like to get to know you a lot. (5)”	In hospital setting patients don’t like to get to know you (5)	
“Yeah, she's the one who assigning patient to me who is you know, there is you patient. And because I always encountered some of the patients who are not so much don't like foreigners. So, but also is experiencing like that kind of yeah. So might be the same they are. (12)”	Some patients don’t like foreigners (12)	
“And if I leave the school, then I will be deported on some kind of things. They told me those things and everything. So it was really. (8)”	If I leave school I will be deported (8)	Having restrictions due to residence permit
“Really, like at some point I went on depression because of all those things because I was like so much pressurised and I couldn't even leave the school because even migri give us a visa according to the tailor-made programme. (8)”	I couldn’t stop the school because of my student visa (8)	
“Through our contract, we are not able to change our courses and you know we cannot get any discounts. So yeah, we cannot any discounts like throughout the year if we got, if we pay the fee earlier like that. Yeah. That's. And like we have to pay the college fee yearly. Like we cannot able we cannot pay the college fee like the half like 3 months. Want and that's a contract, yeah. (18)”	Because of the contract we cant pay the tuition fee dividedly (18)	Having restrictions due to contract with recruitment company
“Include reasonable restrictions like non-compete clauses. (22)”	Contract includes restriction about non-compete clause (22)	
“Yeah, like we have a contract. Contract agency and us like. As we came through the tailor made programme and like we can not able to exchange the outputs like when we arrive the Finland and when we study at the some half of our course, then if I don't want to continue the nursing course then we cannot able to change our course.	We can’t change courses at school because of the contract we made (18)	

Yeah, that's the things they. (18)”		
“But of course I at times I feel like I'm burdened. (3)”	Sometimes feeling like a burden at work place (3)	Feeling that colleagues are tired of giving guidance at work
“Of course, maybe I do understand in some part that people are also tired to, to explain to, to tell, and yeah, maybe we're all just tired, that's why. (4)”	Feeling that others are tired to explain things (4)	
“To be honest, they are not teaching you should do this you should do that you should not do this. You should not do. They are not teaching that. So what we have to do is to just observe them so. And also we don't know if our if what we are doing is correct or not because no one is correcting us. Yeah. So I'm not sure if that is also part of your culture, if there is some somebody's doing a mistake, you will not confront that person, that they aren't doing the wrong thing. I'm not sure if that is your culture, but that's what I observed because. Oh, when we I think. Yeah. When we do something that is not it is wrongfully done we are just informed by our Philippe fellow Filipinos that what we did is not a correct way so. (19)”	We are just being corrected by our fellow Philipino colleagues not by Finnish colleagues (19)	
“But I was curious about the things because this is a whole new different environment for me to work. And then sometimes I feel like asking, but the looks that they give me and the tone that they use for me, it's like completely different. So I prefer not to ask them anything. (8)”	I want to ask something at work but I don’t because of the looks others give to me (8)	
“But I I feel like it's giving. I'm giving more work because I needed special. (3)”	Feeling like giving more work as an international nurse at work place (3)	
“Then. So tell me. No, you have to go only with your or here. Like she doesn't accept me to be with her, you know, to help her. Yeah, you can feel it. You know you this things. You can feel it from the. (10)”	My perceptor didn’t accept me to be with her (10)	
“And things to do, but like very small things like, you know, taking out the trash and like cleaning the floors and stuff like that. But even if that's an equal task that everyone should do, it's like we notice that.	Others boss around foreigners at work (5)	Negative experience as an international nurse at work due to attitude

They boss around foreigners, you know, to do these kinds of tasks for them. (5)"		of colleagues
"So yeah, it's a mix of positive and negatives, yeah. Actually the colleagues, they know that I'm a foreigner. The patients, some thought I have already lived there for long since the way I speak to them they thought that I already know how to speak. Some say that oh, you just arrived here and you speak you went finnish already like that. I'm just saying that I only know basic words and I'm still learning and in terms of my colleagues. They are trying to speak to me in English, some of them who know how to speak English but I am forcing them to communicate with me in Finnish so that. (7)"	Experiences as an international nurse regards to colleagues is a mix of positive and negative (7)	
"Also good and bad experiences, as I can get help from people around, also people around me can improve there English. Although I think not just me, but many nurses, especially in the gerontology field would have bad feelings with people's talking, some time from the clients and sometimes the attitude of the colleagues. (22)"	Bad experience about the bad attitude of the colleagues (22)	
"Oh well. In our first day we are just like statue in because maybe because we are this new and some of our colleagues are especially the Finnish colleagues they are not still approaching us so. When they need to do something they are not asking us for, to, to go, then to observe and. They are not also teaching us to be honest, because maybe because of the language barrier because. Yeah. (19)"	Some of our colleagues are not approaching us to help them (19)	
"Yeah, as usual there, there are some staff here they are even not telling good morning or hi or something. Not supportive at all. And there are some nurses are supporting but I am thinking that Finnish people are. Maybe feel shame maybe feel worry about the international people so. I am considering all of this, but I am taking what I want to take. I will I am taking it politely, and I'm never mind about those people who are not polite or not supportive to me. I'm not never mind about all. I'm just focusing on myself. I am taking as I told you, I'm taking what I	Some colleagues are not greeting me (20)	

want. And by the way, which I want to take it politely and I'm not taking care of those people who are not supporting me. They maybe they are a shame.<br>Yeah. So you know, you know, Finnish culture. (20)”		
“Well, my first two weeks in laakso was really hard for me. I felt that I I'm I've been. I was not welcome because I'm a new face. (16)”	First two weeks at work I felt I’m not welcomed (16)	
“They think that I don't really understand what they're talking about. So they're talking there and they're talking about me and they look at me as if I don't know what they're talking, but I just let it go. It's just that kind of bullying. But, you know, it will pass as long as you stay there and just do the work thing. It's part of the, I don't know if it's part, but it really happens. I know and I understand. (16)”	Bullying at work from colleagues (16)	
“He or she might be the very he, or she might be the best worker, or the best person to work there. But someone is getting intimidated by them, and that won't result in a good way in the workplace. So maybe not putting them together will be the best idea. (8)”	Feeling intimidated from some people at work (8)	
"Yeah, social distance I know you have this one, this, this kind of culture. So if we go near beside you, may be you may feel and I called uncomfortable. Yeah, you felt uncomfortable. So we are just waiting for Finnish colleagues to approach us and to ask us to go with them. That's the time when we go beside them. So in our. To wrap it up, yes, in our first few months. There are there are there are separating their themselves with us but. (19)”	In our first few months our colleagues are separating themselves from us (19)	
“So they will, but I when I came Helsinki here I am just home and hey, I was just when you are asking them something 'cause they are not answering you politely they are. Oh, what is this? I don't know. What is this? Actually, I have maybe I am I, but I don't know. But I'm respecting this culture. They're culture like that. It's OK. So that's as I told you, I'm focusing on myself. I am never I'm not taking care of those people. Yes. (20)”	Sometimes colleagues are not answering to me politely if I ask something (20)	
“And the way that I have	Some	Negative

experienced that is. They don't do it like, you know, face to face. It's not like to face discrimination that I don't like you or something like that. But it more comes out like the discrimination comes out in the work side. So, you know, they give you more responsibilities and they boss you around, even if it is there, you know that it's their job to do something. But you know, they just sit down and tell you to do it. And yeah, so stuff like that. So more like passive aggressive when it comes to job. (5)”	coworkers dicriminate by giving their work for you (5)	experience as an international nurse at work due to discrimination	
“Changing shifts. So like if it's a hard shift because depending on the schedule like for example Mondays usually have are more busy compared to weekend shift, so like they put the foreigners in the busy days and you know some things prefer the weekends and stuff like that so. (5)”	Coworkers discriminate by changing shifts (5)		
“So that's why when I take the LOVE it's really different. Yeah, the practise and the protocol is really different, yeah. (11)”	The practice and the protocol is really different (11)	Practise and protocol is different at work in Finland	Challenges and problems of working life
“We don't do these things at all. Like, we don't waste the time and preparing the medicine every shift or every day it's for me it's not a quality thing or not the way. Like you know, you study something and then the actual life it's not the same study this automated medication, car and power code system. Yeah, actually it's not. It's not the same. (10)”	Studying is different than the practise (10)		
“But somehow I learned some new skills. Like this, taking care of patients. Elderly patients, because I did not take care before an elderly patients in the Philippines. So even though I'm not, I'm not learning a new skills. I I'm I what do you call it? I'm not learning nursing skills here but I'm also learning other skills. (19)”	I’m not learning nursing skills in Finland but I’m learning other skills (19)	Not being able to develop nursing skills at work	
“I think not only me, but no one else also. (8)”	I haven’t developed my personal skills (8)		
“But you know how? l so I will cover everything. How I will work and study and study the language. Now I'm thinking about this also I have to, you know, to make my work like this working hours. (10)”	I can’t work and study and study language same time (10)	Having to study while working	

"It for I think it's OK because I'm not in hurry to come here. It feels for me it's like OK, it's OK working and then after that language training at night. So for me it's OK. But the other some. Yeah, some maybe it's very hard to deal with it because they have also a night shift and then like that. So but for me because I'm morning shift so it's OK. I can go. Yeah. (13)"	For me it was ok to study at night cause I'm working on day time (13)	
"Yeah. So it's been very difficult to study and working at the same time, but. (12)"	It's been difficult to study and work same time (12)	
"So far I have a little bit of struggle in studying the Finnish language, because to be honest I'm still working that time the Philippines as a nurse in a port and I'm just only studying at night after I arrive in my home after my long day of work. So tiring I didn't know. How did I pass that? Because I still had a lot of things that need to prioritise, just like my kids. I need to bring them to their school. In the morning and then I still need to pick up them then after that. instead of resting at night, I'm still need to study this language so far are you able to conquer it? That's why I'm here now. (17)"	Struggling with studying finnish language same time as working (17)	
"In the middle? Oh yes, because you know, it's for, of course, it's hard language and sometimes the teachers there are, even if it's free language. But sometimes the teachers there are, they interview one-on-one and then by finishing language and then we have some difficulties, of course, because I work there while working I have to study at night in language training. So that's just like that but. (13)"	Having difficulties with managing the time for the studies cause we work same time (13)	
"When I when I was lähihoitajaopiskelija. Yeah, I was tied up in a contract for good for two years or three years, I think, but when I graduated and we had a new boss in the work site, it was replaced, but that it was replaced. That you can leave whenever you want. You can do keikkari as long as. (16)"	First i was tied up for a contract for two to three years (16)	Having restrictions due to contract with employer
"Yes, it says there that within the period of while I was like in the. What was the while I was in that? Practise like probationary period.	During probational period we were not	

We cannot like go or work from for other companies or other we cannot take any like part time jobs. But after that when because we already finished the practical nursing course? Yeah, we have been like allowed to take part time jobs from other to other companies. Yeah. (21)"	allowed to work for other companies as part time (21)	
"Yeah, as usual there, there are some staff here they are even not telling good morning or hi or something. Not supportive at all. And there are some nurses are supporting but I am thinking that Finnish people are. Maybe feel shame maybe feel worry about the international people so. I am considering all of this, but I am taking what I want to take. I will I am taking it politely, and I'm never mind about those people who are not polite or not supportive to me. I'm not never mind about all. I'm just focusing on myself. I am taking as I told you, I'm taking what I want. And by the way, which I I want to take it politely and I'm not taking care of those people who are not supporting me. They may be they are a shame. Yeah. So you know, you know, Finnish culture. (20)"	Some colleagues are supporting me (20)	Feeling of not having enough support at work
"Like hiring must be the priority but eventually, after a week or a month or two-month, you should have sit down with that new employee and ask about the environment. (8)"	Employer should have discussion with employee about how things are going (8)	
"Except the healthcare field, I don't have quite any experience in any other field to be honest, and I enjoy this field so much. But in Nepal I was a sairaanhoitaja, I had a registered nurse, but here my degree doesn't comply with that. So I have to start from the very first and as soon as I completed my enough credit, I was not getting a job. (8)"	I was not getting a job as a nurse in Finland after graduating (8)	Challenges with employment in Finland
"In the healthcare field, the opportunity of jobs is quite wise, otherwise, they prefer Finnish, so there are many job fields that I have never been touched. (22)"	In health care field in Finland many jobs prefer Finnish people (22)	
"So for me, I had to send out my application personally I had to look for. I had to network and I had to	I had struggles to find a job with Finnish	

see any job opportunities that I can do here in *** and because I was still very new, I had struggles with the language with Finnish language so. It really took several months before I could find a part time job here. (5)"	language (5)	
"For a few months and it I mean, I was left insane. OK, so it was difficult for me because I needed to apply for my job personally. So there's no there is no help from the school. (5)"	For few months it was hard cause I had to apply the job myself (5)	
"Given a job because of my Finnish language, so again I cried. I cried every night and I said, oh, actually I want to end my life already. I end up to that point where in I don't know how to, how can I go with the flow. How can I live here without a job without money, so paying my rent fee is not that it's very difficult where in your head nothing to get. Yeah. Nothing to support you. So my family send me again money to sustain my rental fees in ***. It's good that it's cheaper because we occupy the student loans. Yeah. So that, that, that was it's in ***. We live ***<br>yeah. So and then it's still two months I have no job. (11)"	Nobody gave me a job because I was not fluent in Finnish (11)	
"Yeah, talking about the job opportunity. I think like it's a little bit hard to get a job. I think due to the language barrier. Yeah. And I think if I have a quite well knowledge then it will be much easier to get. Yeah, I got a lot of feedback. When applying the job. Yeah. So yeah, the main thing is the on. And another thing is like the reference, the a lot of reference used in Finland. So yeah, if I have anyone of them who can give me a reference to applying the job and that is also a great idea, yeah. (18)"	It's a bit hard to get a job in finland (18)	
"Career plan. No, we we don't have because she's not always in our work. It's very seldom that we can that we will see each other. (17)"	We didnt' have career plan discussion (17)	Not having career planning discussion
"Yeah, you, you mean career? No, I think no. Only when we have this kehityskeskustelu. With the yeah, with the boss only during that period, yeah. (21)"	I didn't have career planning discussion (21)	
"No, still actually it was supposed to be last week last week but pomo she was in, she was in vacation, but	I will have my career plan discussion	

she will come for she will come tomorrow. She will start and maybe I am elaborate tomorrow. So maybe I will meet her tomorrow or it will be this week. Just will be orientation. How are you now? How do you feel now? Because she asked me in the beginning. I told her I don't know. I feel very strange here. Nobody's talking with me. Nobody is supporting me. But I think it will be this week and I hope I hope everything will be OK. (20)”	with my supervisor next week after her holidays (20)		
“No, absolutely not. (8)”	No career plan discussion (8)		
“Career plan. Not yet at the moment, but I wanted to after I studied the life I wanted to pursue as a sairaanhoitaja. (14)”	I didn’t have career planning discussion yet (14)		
“Hmm. No, but hey just telling me everything that you understand that language is very difficult, but I am pretty sure that you can handle this. Ask find rose by and. Who knows? Yeah, they told me that. Who knows, as time goes by that you can speak very well, you can communicate very well with the patients. And then. Yeah, we don't know what will gonna happen next. (12)”	No career plan discussion with me (12)		
“I don't have any any training yet (from my workplace). (12)”	I don’t have any training yet from my workplace (12)	Haven’t received training at work from employer	
“At work not at the moment. We don't have any training yet. (14)”	We don’t have any training yet at work (14)		
“Yeah, yeah, yeah, yeah. Very good. Even when we came here at first. So we, we have this gift from our employer. There at least they think they think they're their stuff. Also we have this like free dining free theatre. Our employer give us for that so. It was a training something like that. Like, that's the one I said. I'm waiting something like that. They think so. (13)”	Our employer took us to the theatre after arriving (13)	Employer giving a gift	Professional Successes and achievements
“Yeah, yeah, yeah, yeah. Very good. Even when we came here at first. So we, we have this gift from our employer. There at least they think they think they're their stuff. Also we have this like free dining free theatre. Our employer give us for that so. It was a training something	Our employer took us for dining (13)		

like that. Like, that's the one I said. I'm waiting something like that. They think so. (13)"		
"Niin niin osastonhoitaja on kysynyt jotain, että mitä tulevaisuudessa haluaisin tehdä. (6)"	I had career planning discussion with my supervisor (6)	Having career planning discussion at work
"Oh yeah, yes, they we have. I have a discussion with my manager about it. So actually. (7)"	Career planning discussion with manager (7)	
"Do you? Yeah, yeah, yeah. But actually in the interview, they ask me already about my my plan or career plans. How do you see yourself after five years and all of those? Yeah, of course. (9)"	I've had a career plan discussion at work (9)	
"When I had my trainings, the school prepared really well and also the supervisors supported me a lot. Orientations were given by the school and supervisors, they also help me with the career plan, I improved everyday. At that time I was in a quite big place so I had never seen the employer. (22)"	School and supervisor helped me with career plan (22)	
"Yes, one time in the coordinator final coordinator visited me because I asked since I am studying as a nurse here. I'll ask if there's a possibility that I will be will become a nurse here in *** also. And she said that they will try to find a position for me and usually there is, but if there's none, they say that they will find a nurse position for me, once I am a very registered nurse, a credited nurse. So that's how it is. (15)"	I had career planning discussion with my coordinator (15)	
"Yeah, you, you mean career? No, I think no. Only when we have this kehityskeskustelu. With the yeah, with the boss only during that period, yeah. (21)"	I have had kehityskeskustelu with my boss (21)	
"The path from the *** government which handles us they came here and they were like hey, are you like do you like to stay here? Like are you like do you think you would stay here for long? Do you like the place? Are you getting used to it? And then when I told them that honestly. (2)"	Employer is having career plan discussion (2)	
"And they're very also considerate, especially with the studies, the scheduling of the studies with the work schedule and the rest period. So it's quite nice because if you're	Employer is considerate about work and rest period (2)	Employer is considerate

in the Philippines, you cannot ask for a day off, you cannot ask for an extra time. (2)”		
“Because they have already considered us. Despite that, we are not very fluent enough to speak and to understand and to write so that when. That app of us being accepted is very. Like. I have no obligation or obstruction about that anymore. (7)”	Feeling that employee has been considerate enough already (7)	
“We're not fully independent because we don't have the language yet, so they give consider consideration. (3)”	Employer in considering that we are not fully independent at work (3)	
“And they're very also considerate, especially with the studies, the scheduling of the studies with the work schedule and the rest period. So it's quite nice because if you're in the Philippines, you cannot ask for a day off, you cannot ask for an extra time. (2)”	Employer is considerate about study schedule (2)	
And they're very also considerate, especially with the studies, the scheduling of the studies with the work schedule and the rest period. So it's quite nice because if you're in the Philippines, you cannot ask for a day off, you cannot ask for an extra time. (2)”	Employer is considerate about studies (2)	
“They make it like easy for us to like, do the study in a systematic way and in in our own pace like we can choose whether we go fast or slow. It depends on how we can manage our time and study because we were also working when we do the study and then the thing that. (21)”	Agency made studying systematic for us (21)	
“But we have an arrangement with our employer that that it's OK that during our day of is our school day and so that we can receive 100% of our salary because some of them it's like they can receive only 80% due to the classes also. (1)”	Not having to work as a part time due to studies (1)	
“Yeah, I'm working now, so I'm in this one cardiac, cardiac and pneumonia ward. And they consider this. I have 12 years’ experience in Kuwait City, so they consider this year of experience in my work and in my salary also. They consider this one yes. (20)”	They consider my previous work experience in my work (20)	
“It's they're they're very racist, even though like I work. Like we have a good team now. There are Filipino	I work in international team (8)	Working as part of international

nurses there. They have like Cambodian nurses and everyone. And if I don't because I don't speak Finnish very well, I cannot speak. I I really cannot speak. It's like a big no no for me. And then even though I try and I, what do we say there even I try and I manage with the osakas. But speaking with the native finish or. (8)”		team
“All support our workplace consists of a lot of international nurses from different countries.<br>So because of lähihoitajas and avustajas coming from Nigeria, Turkey, Ukraine, so it is common for us that. (5)”	International work team (5)	
“We also have every Friday we have like a team, a meeting with the team where we give each other feedback. If there's any feedback or any questions that we want to ask. (5)”	Weekly team meetings at work (5)	Having regular meetings at work
“No, they're actually enough because they, my manager, always and we always had this meeting like this small talk with the supervisor. So like, how are you? How are the things going? How is your language proficiency? So he's very supportive. (14)”	My manager has meetings with us askinh how are you (14)	
“Us my colleague, just because we have this thing that if you have, if you want to say something, we have also meeting in my workplace that every once a month and then if you have for example, if you have a problem or if you have something you can, you can say yes in the meetings. For example, we are in the meeting and then we have you. If you want to say something that is make you uncomfortable, you can say it to. (13)”	We have meetings at work every month when we can give feedback or ask something (13)	
“Yeah, and in the ward, they also update us every meeting. It's just a quick meeting, but it's an update about the system, the operating system. (7)”	Regular meetings at the ward (7)	
“Of the company, yeah. Yeah, there is always a actually it is a weekly basis. Yeah, the meetings, yeah. And then we, aside from the company, from the pomo, we also have our sairaanhoitaja focus. Yeah, we discuss everything. So actually I want to. What's that? Improve. Actually I want to improve the the things they are used to do which is not that is not within the standard.	We have meeting in our work place on weekly basis (11)	

(11)”		
“And some sometimes our manager also have this meeting with us and that's the time that she also teaching us regarding the process in the nursing home and maybe some of our Finnish colleagues maybe reported the things that we need to learn and also the things that we did wrong and sometimes. (19)”	Sometimes our manager is having meetings with us and teaching us regarding the process in nursing home (19)	
“I think yes because I still am learning, you know, but also. They do, we do have like regular meetings. (2)”	We have regular meetings at work (2)	
“In terms of like, oh, when there's a meeting with the lääkäri, they push me to attended so I can learn more with the process. (16)”	They develop my skills at work by cooperating with the doctor (16)	Being able to develop skills at work
“Everything is like high tech, everything is updated since I've come from a third world country like the Philippines, always relax one step behind the other countries so experiencing living and working in a highly advanced countries very. It's like an update for me. Everything is an update. (7)”	Working as a nurse in Finland is a personal update due to high technology (7)	
“Sufficient time it will be how long. I don't know. For how long, I don't know. But I think now I am one month now in this sydänosasto, I feel I know too many things I know now. Maybe after two months is 3 months is I will be more professional. But time. (20)”	Learning many things at work in one month (20)	
“Yeah. Yeah, it's a continue process to develop the skills. Yeah, we can enough to develop the skills as we yeah. So it's the continuous process. And yeah, I'm trying to be and I will try to develope the my skills continually. Yeah. (18)”	It’s a continuous process to develop my skills (18)	
“Colour tooth, even in online and in personal right, so they help us to develop our skills by doing the koulutus and at the same time we do it on our own with the patients. We apply it like we are hands on in everything so. (9)”	They help us to develop our skills (9)	
“How you take care of people who is dying, and especially with the special medications like strong painkillers using the kipupumppu and the all others, so it is a really good experience for me. (9)”	Getting good experience from work (9)	
“Yeah, I've I've improved all my my skills in nursing. So I think that.	Being able to improve all	

Yeah, it helped me much when I did my, my, my. (9)"	nursing skills (9)	
"So I was able to get a contract for lähihoitaja and I have been working with them as a keikkari for 10 months now. So from for that same company and it's it's been good. I have learned a lot and I've learned a lot of like language and nursing skills from there as well, so. (5)"	I have learned a lot about nursing skills at work (5)	
"So I was able to get a contract for lähihoitaja and I have been working with them as a keikkari for 10 months now. So from for that same company and it's it's been good. I have learned a lot and I've learned a lot of like language and nursing skills from there as well, so. (5)"	I have learned a lot at work on language wise (5)	
"But somehow I learned some new skills. Like this, taking care of patients. Elderly patients, because I did not take care before an elderly patients in the Philippines. So even though I'm not, I'm not learning a new skills. I I'm I what do you call it? I'm not learning nursing skills here but I'm also learning other skills. (19)"	I'm not learning nursing skills in Finland but I'm learning other skills (19)	
"I think everything has improved because the standard of work here is very high and it's the quality of work is very like top class so. (7)"	In terms of work everything is an improvement (7)	
"It's they are helping me to develop my skills.<br>In now they are teaching to give medicine and and if I didn't know anything, he told me to just ask question if I'm if I didn't know something. (17)"	My colleagues are helping me to develope my skills (17)	
"I think there was no restriction about thing I guess. But I mean we need to stay in *** for three years. (14)"	We have a contract with our employee for three years (14)	Medium-term employment contract
"Loaded in the no specific but of course our contract is under, so for that we are contract is under *** is 2 years so we can't of course we can apply the in other employee employers so. That's it. We know that we can apply it to other companies or employers then other than I know. (15)"	My contract is for 2 years (15)	
"I mean two years contract, so we can just still want to continue to relocate. As you know, I think. (2)"	Having a contract for two years (2)	
"Well, we have a contract for at least two years. That's our contract.	Having a contract for	

(1)"	two years (1)	
"We receive our E pass on that time and they told us we are regular already, that's it. (1)"	Receiving E pass (1)	Receiving benefits at work
"Baby, the company paid that and they also they were giving also laptops and books at my time. (4)"	Company paid for book and laptops (4)	
"And then when we came here in Finland, you know, we have an organisation of Helsinki for three days and then they give us some allowance. I think I had low 500 euro from them. They've given us for a start up and then. (4)"	Getting allowance (4)	
